# Biophysical modeling of anatomically realistic prenatal cortical folding development

**DOI:** 10.1038/s41467-026-75582-9

**Published:** 2026-07-16

**Authors:** Jixin Hou, Zhengwang Wu, Kun Jiang, Taotao Wu, Lu Zhang, Dajiang Zhu, Wei Gao, Mir Jalil Razavi, Tianming Liu, Ellen Kuhl, Gang Li, Xianqiao Wang

**Affiliations:** 1 School of ECAM, College of Engineering, University of Georgia, Athens, GA USA; 2 Department of Radiology and Biomedical Research Imaging Center, The University of North Carolina at Chapel Hill, Chapel Hill, NC USA; 3 School of Chemical, Materials, and Biomedical Engineering, College of Engineering, University of Georgia, Athens, GA USA; 4 Department of Computer Science, Indiana University-Indianapolis, Indianapolis, IN USA; 5 Department of Computer Science, University of Texas at Arlington, Arlington, TX USA; 6 Department of Biomedical Sciences and Imaging, Biomedical Imaging Research Institute, Cedars-Sinai Medical Center, Los Angeles, CA USA; 7 Department of Mechanical Engineering, Binghamton University, Binghamton, NY USA; 8 School of Computing, University of Georgia, Athens, GA USA; 9 Department of Mechanical Engineering, Stanford University, Stanford, CA USA

**Keywords:** Computational biophysics, Biophysical models, Biomedical engineering

## Abstract

Cortical folds encode the architecture of human cognition, yet the mechanisms that transform the smooth fetal cortex into its convoluted geometry remain elusive. Biophysical modeling enables mechanistic insight into cortical morphogenesis, but existing models often lack anatomical realism and fail to capture key hallmarks and morphometrics of dynamic cortical folding in the developing human brain. Here, we introduce a whole-brain developmental framework that integrates region-specific, data-driven growth laws with anatomically realistic cortical geometry to enable biologically interpretable modeling of cortical morphogenesis during gestation. Growth fields derived from large-scale prenatal magnetic resonance imaging data capture spatiotemporal variations in cortical expansion and thickness across parcellated regions. Incorporating heterogeneous growth yields folding patterns that match key anatomical landmarks and quantitative morphometrics from human imaging. Systematic perturbations of geometry and growth attributes delineate control parameters that produce realistic morphological variability and replicate clinically atypical brain phenotypes consistent with lissencephaly, pachygyria, and polymicrogyria. This framework provides a quantitative foundation for elucidating the mechanisms of typical and atypical fetal brain development.

## Abstract (web summary)

Using fetal MRI data and biophysical simulations, this study shows that region-specific cortical growth helps shape realistic brain folding and provides insight into normal development and congenital malformations.

## Introduction

The human brain undergoes remarkable morphological changes during gestation, giving rise to the highly convoluted folds of the cerebral cortex^1^. This folding process, which intensifies during the third trimester, coincides with rapid volumetric expansion and surface growth that foster neuronal proliferation and promote efficient information processing. Alongside the emergence of intricate folding morphology, cortical development exhibits pronounced spatiotemporal heterogeneity^2^. Cross-sectional neuroimaging analyses reveal distinct regional trajectories of cortical thickness and surface expansion across gestational ages^3,4^. This regionalization is further supported by transcriptomic and cellular evidence showing that spatially patterned gene expression regulates neural progenitors proliferate, differentiate, and form cortical layers, leading to divergent growth dynamics across cortical regions^5–7^. However, despite increasing evidence of heterogeneous growth signatures, how they physically translate into the complex folding patterns of the cortex remains poorly understood.

Cortical folding displays both consistent and variable features across individuals. Primary folds arise at stereotypical locations around mid-gestation, forming early landmarks such as the central sulcus and Sylvian fissure that are conserved across individuals. In contrast, secondary and tertiary folds emerge later, enriching local surface complexity and contributing to inter-individual variability^8,9^. This coexistence of regularity and variability points to a potential interaction between regional growth dynamics and geometric constraints^10^, yet their specific roles in shaping cortical folding patterns remain unclear. When normal developmental processes are disrupted, the resulting cortical malformations, characterized as atypical folding geometries, are clinically associated with neurodevelopmental disorders such as epilepsy, schizophrenia, and autism spectrum disorder^11–13^. Clarifying the developmental mechanisms underlying these malformations is therefore crucial for uncovering their pathological origins and for informing early diagnosis and risk assessment.

With advances in computational and biophysical techniques, in silico modeling has emerged as powerful approach to simulating the complex spatiotemporal development of cortical folding within a physically grounded framework. To this end, various hypotheses and theories have been proposed to incorporate distinct biological mechanisms^10,14–18^. Among them, the prevailing explanation is the differential tangential growth hypothesis, which posits that deformation mismatch between the expanding cortical plate and the underlying subcortical tissues drives cortical gyrification^19–23^. While this paradigm has yielded important insight into folding mechanics, current models often yield simplified or generic patterns that fail to capture the anatomical complexity observed in human fetal magnetic resonance imaging (MRI) data. This discrepancy largely arises from the use of conceptual geometrical models and oversimplified growth representations. To approximate brain geometry, many models adopt generalized shapes such as spheres or ellipsoids^24–27^, which capture the brain gross morphology but lack regional anatomical details. Although some studies have employed reconstructed brain surfaces as simulation domains, they generally apply globally uniform growth assumptions derived from bulk volume approximations, which fail to account for region-specific developmental dynamics^10,22,23^. This raises a key question of whether uniting anatomically realistic brain geometries with biologically grounded growth representations can more faithfully capture cortical morphogenesis.

In this study, we present an advanced whole-brain computational modeling framework that integrates heterogeneous developmental growth laws to simulate the intricate prenatal cortical development with anatomically realistic folding patterns. Built upon prenatal brain geometries at 22 gestational weeks (GWs), the framework incorporates atlas-based regional growth laws derived from large-scale imaging-based measurements of cortical expansion and thickness spanning 22-40 GWs. We show that heterogeneous growth generates folding patterns with high anatomical fidelity, closely matching both cortical landmark features and quantitative morphological measures. Systematic variation of geometric factors, including the initial cortical surface configuration and cortical thickness, together with growth factors such as trajectory and magnitude, further demonstrates how their interplay shapes distinct folding morphologies. Beyond reliably modeling cortical development, our framework provides a generative platform for generating numerous anatomically realistic synthetic fetal brain datasets, potentially mitigating the scarcity of longitudinal prenatal imaging. The framework also sheds insight into the mechanistic, evolutionary, and clinical aspects of brain morphogenesis.

## Results

### Growth trajectories are heterogeneous across brain regions

**Fig. 1:**
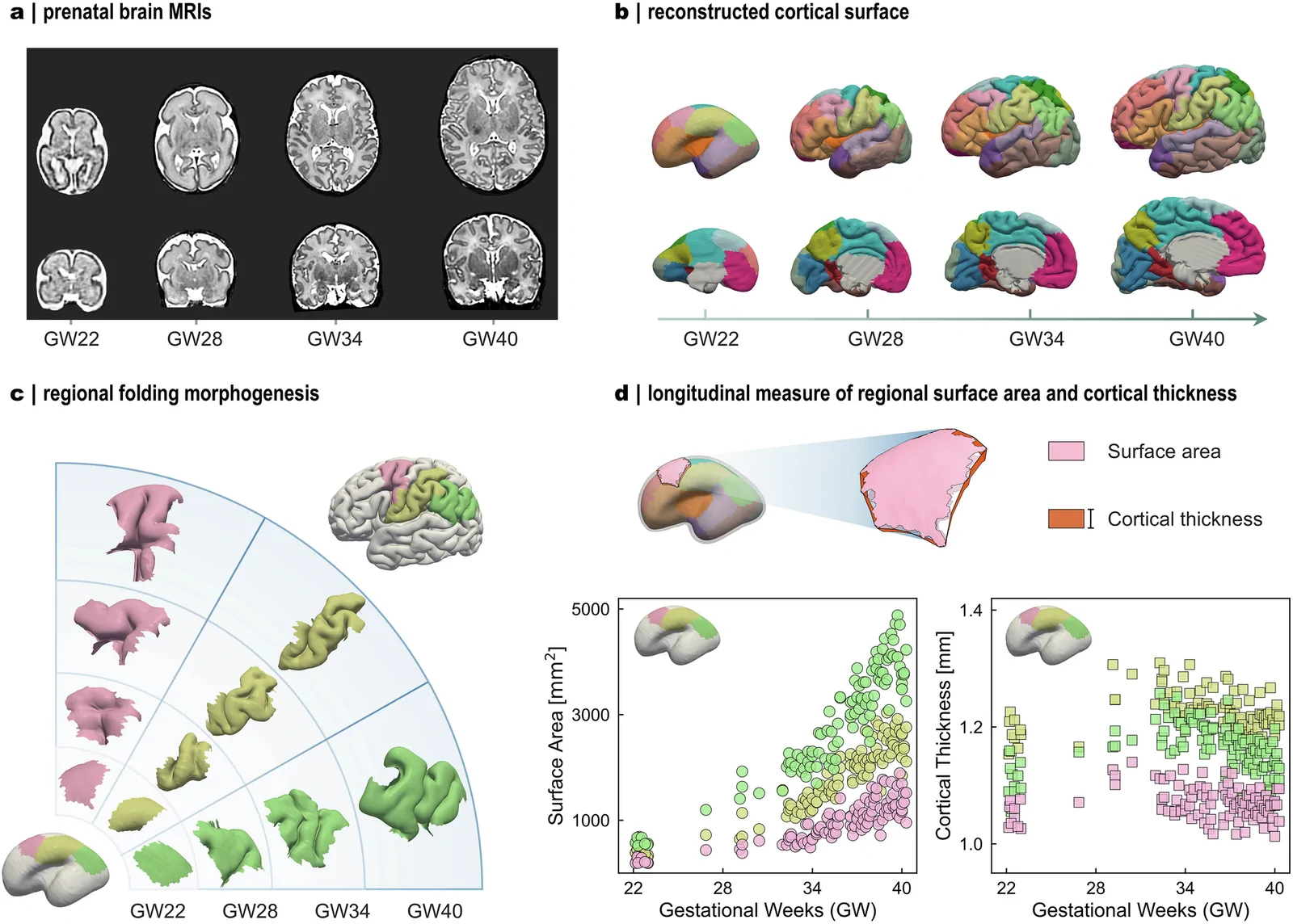
Heterogeneity of cortical development during the prenatal period. **a** Prenatal MR images from 22 to 40 gestational week (GW) in axial and coronal views. **b** Reconstructed cortical surfaces from MRI data (two views), colors indicate regional parcellation. **c** Regional morphogenesis of cortical folding during development, illustrated with three example regions. **d** Developmental measurements of surface area and cortical thickness in the corresponding regions. Data are collected from 120 subjects.

**Fig. 2:**
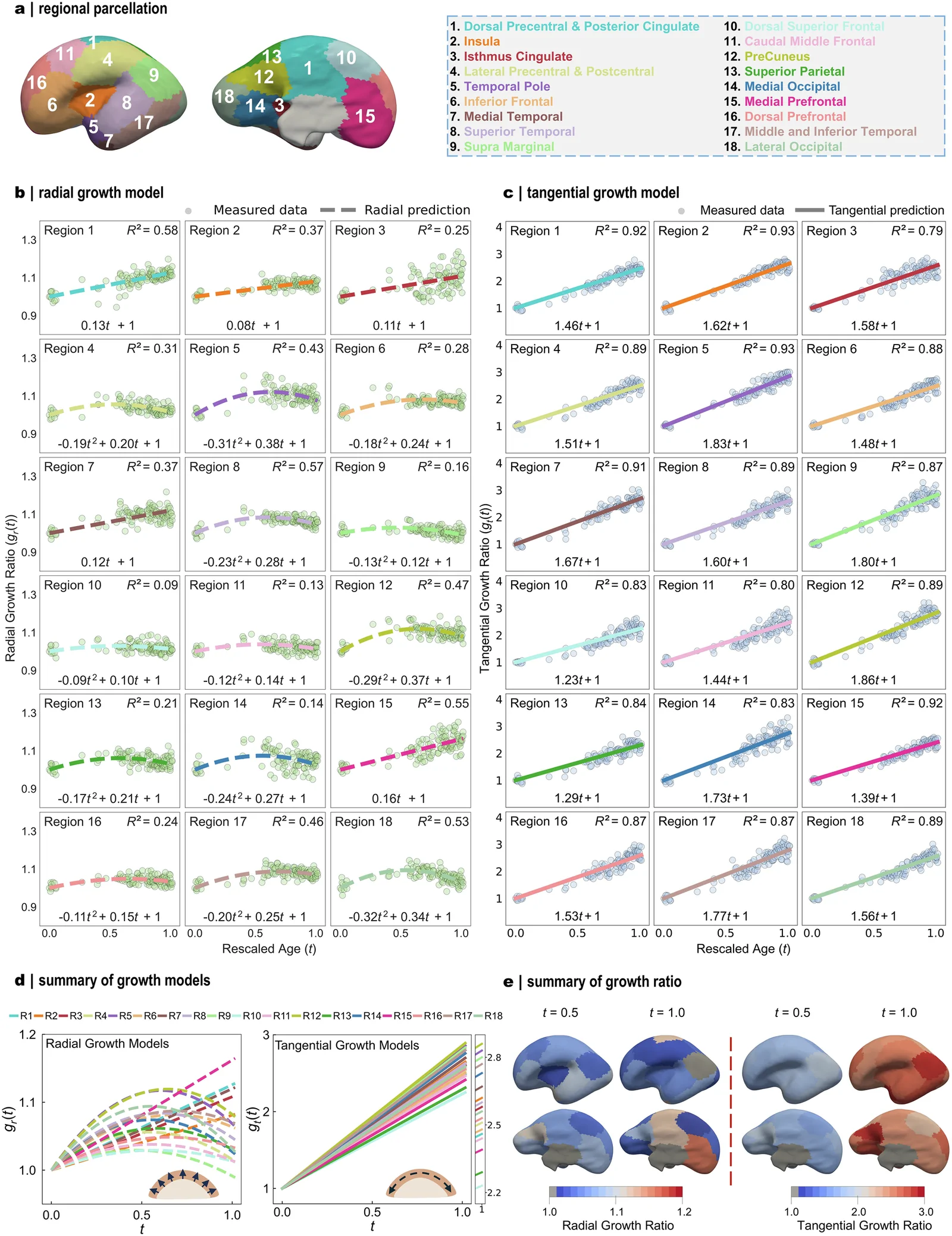
Characterization of region-specific growth models in the left hemisphere using symbolic regression. **a** Developmental parcellation maps with corresponding anatomical regions. Distinct colors indicate different regions, with corresponding label numbers provided; the associated anatomical regions are listed in the right panel. **b**,** c** Growth models identified in radial and tangential directions. Dots denote MRI data from 22 to 40 gestational weeks; lines indicate fitted growth models, with mathematic formulas shown at the bottom of each subfigure. *R*^2^ indicates fitting goodness. **d** Summary of identified regional growth model in both directions. Insets illustrate schematic representations of radial (left) and tangential (right) growth; the right inset provides a magnified view of the tangential growth ratio near t = 1. **e** Growth ratio values visualized on the brain surface at 22 GWs for two developmental stages. Source data are provided as a Source Data file.

We first quantified regional cortical thickness and surface area in 120 fetal brains spanning 22-40 GWs from the developing Human Connectome Project (dHCP) dataset. Using a developmental atlas designed to parcellate the prenatal and neonatal cortex, we characterized region-specific morphogenetic patterns across this period (Figs. 1a–d, 2a). These cortical regions exhibited distinct developmental trajectories, as reflected by divergent changes in cortical thickness and surface area from 22 to 40 GWs (Fig. 1c, d).

To enable cross-regional comparisons, we normalized all measurements to the mean value at the earliest developmental stage, 22 GWs, to obtain relative growth ratios. Using a data-driven symbolic regression approach (Supplementary Fig. 1), we derived mathematical models to describe region-specific growth dynamics. These models separately characterized radial growth, defined by changes in cortical thickness (Fig. 2b), and tangential growth, defined by changes in cortical surface area (Fig. 2c).

Both radial and tangential growth show pronounced regional heterogeneity during development. Cortical thickness followes several distinct trajectories: a steady increase, as observed in region 1, covering the dorsal precentral and posterior cingulate areas; an early plateau, as seen in region 10, corresponding to the dorsal superior frontal region; or a biphasic pattern. In the biphasic pattern, cortical thickness peaks around mid-gestation ( ~ 31 GWs) and subsequently declines modestly (region 18: lateral occipital) or stabilizes at a plateau (region 17: middle and inferior temporal). These characteristic trends are consistent with previous fetal MRI observations^28–30^. The non-monotonic decline in thickness likely reflects localized microstructural remodeling, including synaptic pruning and dendritic compaction, that reduce apparent cortical thickness^31^. In contrast, tangential growth consistently exhibits linear trajectory, with rates substantially exceeding those of radial growth. This rapid prenatal tangential expansion drives cortical enlargement and promotes the emergence of folds. The associated increase in cortical surface area provides space for proliferating neurons and helps establish the structural foundation for nascent brain function^5^.

These regional distinctions demonstrate that cortical development varies across both space and time, resulting in spatiotemporal heterogeneity across brain regions (Fig. 2d). To further characterize these patterns, we summarized the regional growth ratios at mid-gestation (31 GWs) and at term (40 GWs) based on the identified growth models (Fig. 2e). Tangential growth ratios are consistently higher than their radial counterparts, and their near-linear trajectories preserve the overall spatial distribution from mid-gestation to term. Notably, temporal, parietal, and peri-Sylvian regions exhibit relatively higher tangential growth ratios, consistent with neuroimaging evidence of accelerated prenatal development in cortical regions associated with auditory and language functions^6,32,33^. In contrast, radial growth displays a different spatial pattern. Cortical thickness increases more rapidly around the central sulcus regions during late gestation, in line with the earlier maturation of primary sensorimotor cortices that support motor control and somatosensory processing^34^. Growth patterns are not fully symmetrical between the left hemisphere (Fig. 2) and right hemisphere (Supplementary Fig. 2). Although the exact regional trajectories differed between hemispheres, both showed broadly similar developmental trends, particularly for tangential growth. Collectively, these findings reveal pronounced spatiotemporal heterogeneity in cortical development, reflecting the inherent complexity in growth dynamics that underlie the emergence of human brain folding.

### Heterogeneous growth produces more realistic folding outcomes

**Fig. 3:**
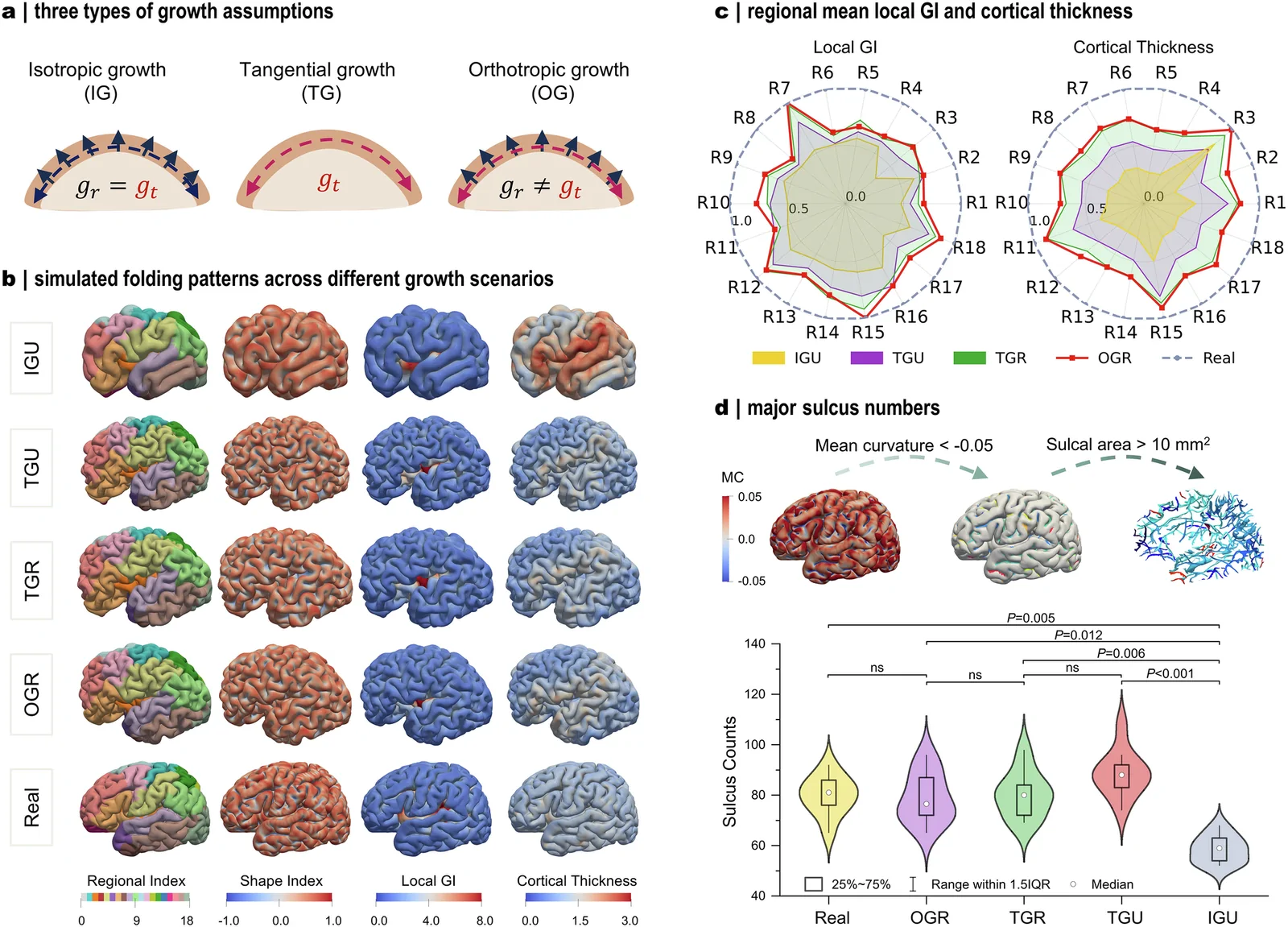
Heterogeneous tangential growth better captures realistic cortical folding patterns. **a** Schematic illustration of three growth assumptions. **b** Simulated cortical folding patterns under different growth scenarios: isotropic growth with a uniform profile (IGU), tangential growth with a uniform profile (TGU), tangential growth with a regional profile (TGR), and orthotropic growth with a regional profile (OGR). From left to right, the cortical surfaces are overlaid with different morphometric metrics. **c** Regional comparison of local gyrification index (GI; left) and cortical thickness (right), where values closer to 1 indicate higher similarity to real brains. **d** Comparison of the numbers of major sulci. Major sulci are defined as sulci patches with mean curvature (MC) < −0.05 and area > 10 mm². Sulcal counts across the cases in panel (**b**) are shown as violin plots (*n* = 50; 10 subjects per case). White dots indicate medians; boxes denote interquartile ranges (25^th^-75^th^ percentiles); whiskers extend to 1.5 × IQR. Statistical significance is assessed using the two-sided Kruskal-Wallis test with Dunn’s post hoc comparisons and Bonferroni correction. ns, not significant. Source data are provided as a Source Data file.

To examine how heterogeneous growth shapes cortical folding, we employed an in silico modeling approach. We developed a whole-brain computational framework with anatomically realistic geometry and region-specific, direction-dependent growth control, allowing growth to mechanically drive brain deformation (Supplementary Fig. 3). The identified regional growth models, including both tangential and radial components (Fig. 2b, c), were incorporated as deformation drivers to simulate cortical folding from early to late gestation (22-40 GWs). This formulation produced orthotropic growth representation. For comparison, we also considered alternative growth assumptions commonly used in classical cortical folding modeling, including isotropic growth (uniform in all directions) and tangential growth (restricted to the in-plane direction; Fig. 3a). To isolate the effect of growth heterogeneity, we further compared two growth profiles across the cortex: a uniform profile, with the same growth applied across all regions, and a regionally varying profile, reflecting the identified heterogeneous growth patterns. Together, these combinations defined four modeling scenarios: isotropic growth with a uniform profile (IGU), tangential growth with a uniform (TGU) or regional (TGR) profiles, and orthotropic growth with a regional profile (OGR). In all scenarios, the subcortex was assigned the same isotropic growth model, characterized from volumetric measurements derived from imaging data (Supplementary Fig. 4).

Varying growth configurations produce distinct folding outcomes (Fig. 3b). Under isotropic growth, the enhanced radial expansion yields less complex morphologies, characterized by bulky gyri and widely spaced sulci. In contrast, tangential and orthotropic growth generate broadly similar folding patterns, with TGR and OGR producing nearly indistinguishable morphologies. TGU, however, shows noticeable deviations, particularly around the central sulcus, where folds are less developed. These differences highlight the critical role of regionalized tangential growth in shaping cortical folding patterns. To quantify these effects, we compared regional averages of the local gyrification index (GI; a measure of folding complexity) and cortical thickness (Fig. 3c). Similarity to real brains was quantified using a similarity score, with values closer to 1 indicating stronger correspondence. Among the tested scenarios, TGR and OGR show the highest agreement across regions, consistent with their visual similarity to real folding patterns. In contrast, uniform growth profiles, particularly isotropic growth, exhibit substantial deviations in both GI and cortical thickness. Similar trends are observed in global GI and sulcal depth trajectories, which further demonstrate closer correspondence to real brains for heterogeneous growth (OGR) compared to uniform growth (IGU) (Supplementary Fig. 5). We further assessed folding complexity by comparing the number of major sulci in the left hemisphere, identified using empirical curvature and patch-area thresholds (Fig. 3d). As expected, IGU produces markedly fewer sulci, consistent with its simplified folding patterns. The other growth scenarios yield sulcal counts comparable to real brains, with TGU showing a slightly higher median, although differences are not statistically significant. Overall, these results demonstrate that heterogeneous tangential growth is indispensable for reproducing realistic cortical folding.

Notably, TGR and OGR differ only in radial growth, with OGR incorporating region-specific thickness regulation. Their similar folding patterns and close agreement in regional metrics measurements suggest that variations in radial growth have limited influence on overall folding geometry under the tested conditions. Despite the inclusion of prescribed thickness growth, simulated cortical thickness deviates from empirical measurements (Fig. 3c; and Supplementary Fig. 6), suggesting that thickness patterns are influenced by tangential-growth-driven folding through geometric and mechanical coupling^35^ (see “Tangential growth-dominated folding modulates cortical thickness variations” in Supplementary Materials). However, comparison between TGR and OGR shows that incorporating region-specific radial growth leads to improved alignment of cortical thickness trajectories with real brain data (Supplementary Fig. 6). This relative improvement indicates that thickness regulation enhances the fidelity of cortical folding simulations. Based on these results, the OGR configuration was selected for subsequent simulations.

### Modeled folding patterns align with those observed in the developing brain

**Fig. 4:**
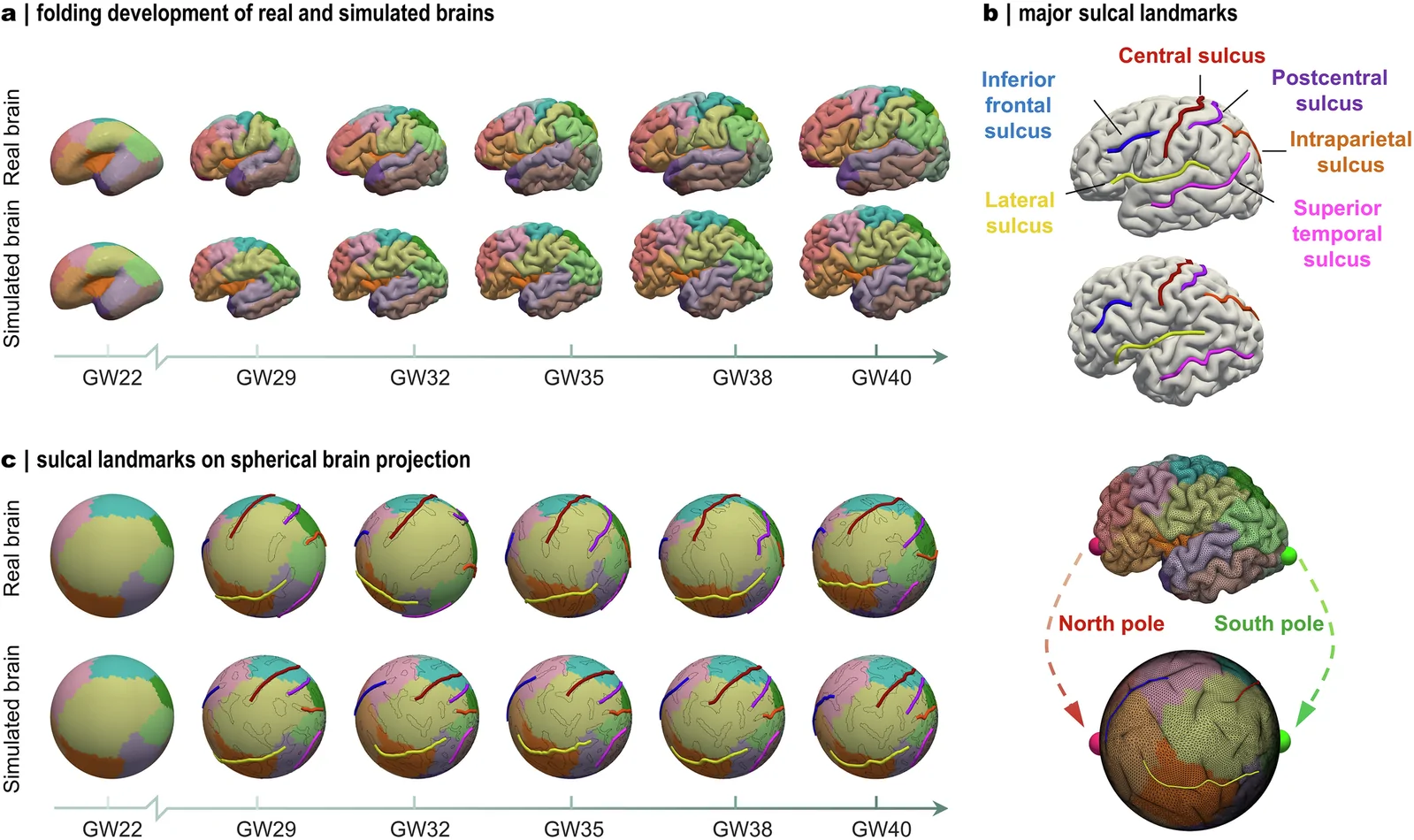
Modeled brains recapitulate realistic developmental folding patterns. **a** Longitudinal folding evolution of one simulated brain from 22 to 40 gestational week (GW), compared with real brains from different individuals. **b** Alignment of six sulcal landmarks between real and simulated brains. **c** Temporal evolution of sulcal landmarks mapped onto the spherical representations of the brains in panel (**a**). Major sulcal contours (black lines) are defined by regions with negative mean curvature. Spatial correspondence in spherical space is shown at right.

In this section, we perform a comprehensive evaluation against real brain data to assess the cortical folding patterns generated by our framework. Starting from an initial smooth geometry, the model progressively generated convoluted folds that conform to realistic brain morphology (Fig. 4). The resulting brains closely resemble developing human brains not only in overall volumetric growth but, more importantly, in the emergence and evolution of major folding patterns across corresponding developmental stages (Fig. 4a). We note that reference brains shown here represent cross-sectional samples from different individuals and serve as developmental benchmarks rather than direct subject-level comparisons. The modeled trajectories indicate that primary folding patterns are largely established by ~32GWs, after which existing sulci mainly deepen and widen in concert with overall brain expansion. This developmental timeline aligns with *in utero* imaging observations showing that primary folds emerge by 32-34 GWs, while subsequent complexity arises through secondary and tertiary fold elaboration^36^. Notably, the framework reproduces key anatomical folding features, particularly the six major sulcal landmarks in both location and relative dimension (Fig. 4b). These sulci are well-recognized markers of cortical organization due to their structural and functional relevance. For example, the central sulcus demarcates the boundary between primary motor and somatosensory cortices, which are essential regions for motor control and sensory perception^37^, whereas the superior temporal sulcus represents a prominent landmark associated with higher-order auditory and language processing, as well as social cognition^38^.

To visualize and compare cortical folding patterns, we projected the brain morphologies onto a unit sphere with overlaid major sulcal contours (Fig. 4c). In the modeled brains, the relative positions of major folds stabilize by ~32 GWs. After this stage, the sulci primarily elongate and widen, while secondary and tertiary branches gradually emerge to further enrich folding complexity. By contrast, sulcal configurations in real brains appear less consistent across gestational ages, reflecting inter-individual variability inherent in cross-sectional datasets. Importantly, the modeling framework allows continuous tracking of folding evolution and can generate brain morphologies at any developmental stage (Supplementary Movie 1). This capability underscores a key advantage of computational modeling: reconstructing the longitudinal progression of cortical folding from a single MRI scan.

**Fig. 5:**
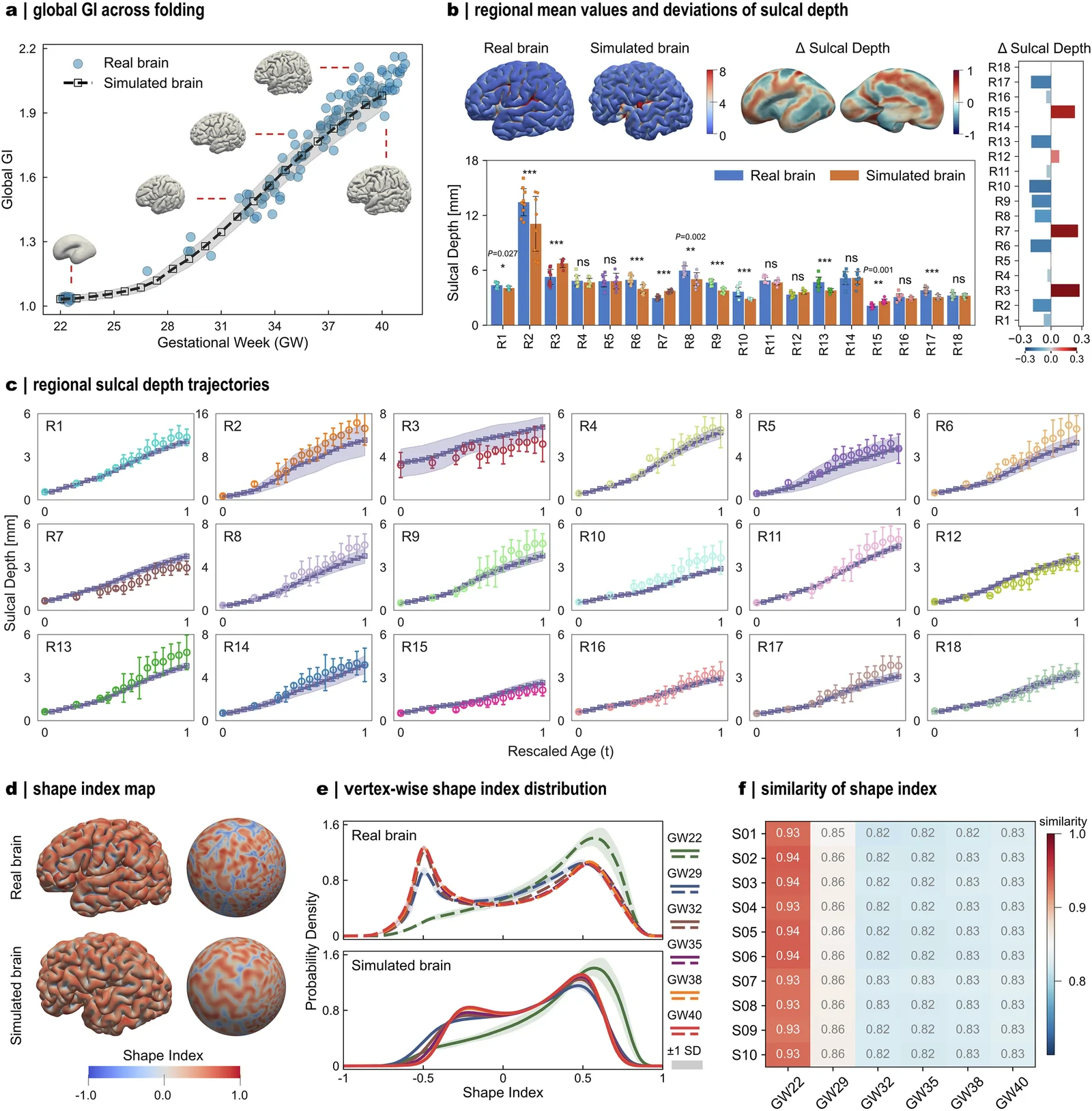
Modeled brains quantitatively recapitulate realistic folding patterns across scales. **a** Developmental trajectory of global gyrification index (GI) in real and simulated brains. Insets show real brain folding patterns for typical data points. Simulation data are shown as mean ± SD (*n *= 10). **b** Distribution of vertical sulcal depth and relative deviation between simulated and real brains at 40 GWs. The top panels show vertical sulcal depth maps for representative brain (left) and the corresponding relative deviation (right). Deviation is computed using the mean sulcal depth from ten simulated brains and the mean sulcal depth from real brains near 40 GWs, and is visualized on the real brain surface at 22 GWs. Region-averaged deviations are shown in the right panel. Regional sulcal depth across 18 cortical regions in simulated and real brains is summarized in the bottom-left panel. Data are presented as mean values ± SD (*n* = 10). Statistical significance is evaluated using a two-sided Wilcoxon signed-rank test. ***P < 0.001; **P < 0.01; *P* < 0.05; ns, not significant; exact *P* values are provided where applicable. **c** Trajectories of regional mean sulcal depth across 18 regions. Simulation data are shown as mean ± SD (*n* = 10). Dots and error bars represent the mean ± SD of measurements from real brains at each gestational week (*n* = 120). **d** Distribution of shape index in real and simulated brains, displayed on folded and spherical surfaces. **e** Shape index probability density distributions computed across all vertices at six gestational ages. Data are shown as mean ± SD. **f** Similarity of shape index between ten simulated brains and real brains, corresponding to panel (**e**). Source data are provided as a Source Data file.

To assess folding fidelity, we computed multiple morphometric and mechanical metrics and compared the results across global, regional, and vertex scales (Fig. 5). At the whole-brain level, folding complexity was characterized using the global GI, which quantify cortical surface convolution relative to its convex hull. During development, global GI shows an accelerated, near-linear increase after ~27 GWs, marking the onset of rapid fold formation. Moreover, the modeled GI trajectories closely mirror those of developing brains, indicating consistent global folding dynamics (Fig. 5a). At the regional level, we evaluated sulcal depth, which directly reflects the depth of individual folds (Fig. 5b). At term, regional mean sulcal depth peakes prominently in the insular regions (region 2: Insula), consistently with their persistent deep concavities throughout development. Across most regions, modeled sulcal depth agrees well with observations in developing brains, with larger deviations in the peri-Sylvian, interhemispheric, and isthmus cingulate regions. These discrepancies likely arise from geometric complexity and intricate fold interactions, which complicate both computational modeling and morphometric quantification. Beyond term-stage comparisons, we further analyzed the temporal evolution of regional sulcal depth by comparing modeled and observed trajectories across gestation (Fig. 5c). Sulcal depth exhibites a biphasic developmental pattern, with an initial slow increase followed by accelerated deepening, transitioning near 29 GWs (t ≈ 0.4). In most regions, the model captures both the trend and magnitude of sulcal deepening observed in developing brains.

We next evaluated folding accuracy at the vertex level using the shape index, a curvature-based measure of local surface geometry. Positive shape index values correspond to gyral features, whereas negative values correspond to sulcal features (Fig. 5d). In both modeled and developing brains, vertex-wise shape index distributions exhibit a characteristic double-peaked profile centered around ±0.5 (Fig. 5e). This reflects the increasing prominence of ridge and rut features, consistent with previous reports^39,40^. After the initial stage at 22 GWs, a pronounced shift in the distribution emerges around 29 GWs, coinciding with the onset of surface buckling that initiates fold formation. As development proceeds, the distributions gradually converge in parallel with the maturation of major folds during late gestation. To quantify vertex-wise similarity, we computed a similarity index between each modeled brain and the average of real brains at the corresponding gestational stage (Fig. 5f). Across all stages, similarity indices exceed 0.8 for all brain subjects, indicating high vertex-level fidelity.

Beyond morphometric analyses, we further examined the mechanical state by evaluating stress distributions during the simulations (Supplementary Fig. 7). Both the spatial organization of stress, characterized by compressive stresses in the cortex and tensile stresses in the subcortex, and the overall stress magnitudes agree with prior computational studies^19,41^ and experimental measurements^42^. Overall, these qualitative and quantitative results demonstrate that the framework faithfully recapitulates realistic cortical folding patterns while enabling continuous reconstruction of developmental trajectories that are difficult to capture from cross-sectional imaging alone.

### Anatomical geometry and regional growth shape cortical folding patterns

In this section, we investigate how anatomical geometry and growth models influence cortical folding outcomes. By systematically examining these factors, we demonstrate the framework’s ability to generate diverse folding morphologies under varying control parameters and to provide insight into the developmental mechanisms underlying cortical folding.

We first examined the role of anatomical configuration, focusing on initial brain geometry and cortical thickness (Supplementary Fig. 8). Although brain morphologies remain largely smooth at 22 GWs, individual variability in geometric features (e.g., surface curvature) results in distinct folding patterns (Supplementary Fig. 8a)^20^. Despite this variability, major features such as primary sulci and gyri are consistently reproduced, underscoring the robustness of the framework. We then investigated the effect of initial cortical thickness by offsetting the cortical surface inward or outward to systematically vary cortical thickness (Supplementary Fig. 8b). Two levels of modification were considered, corresponding to 40% and 80% of the mean initial thickness. Increasing cortical thickness results in fewer folds with bulkier gyri and deeper sulci, whereas decreasing thickness leads to more densely distributed folds with smaller gyri and shallower sulci. These trends are consistent with core-shell buckling theory, which predicts reduced folding complexity with increasing shell thickness^43^. Quantitatively, regional mean sulcal depth increases with thickness across all regions, while the number of major sulci increases as thickness decreases (Supplementary Fig. 8c,d). Together, these results demonstrate that variations in initial geometry and cortical thickness modulate folding morphology while preserving key anatomical features observed during early brain development.

**Fig. 6:**
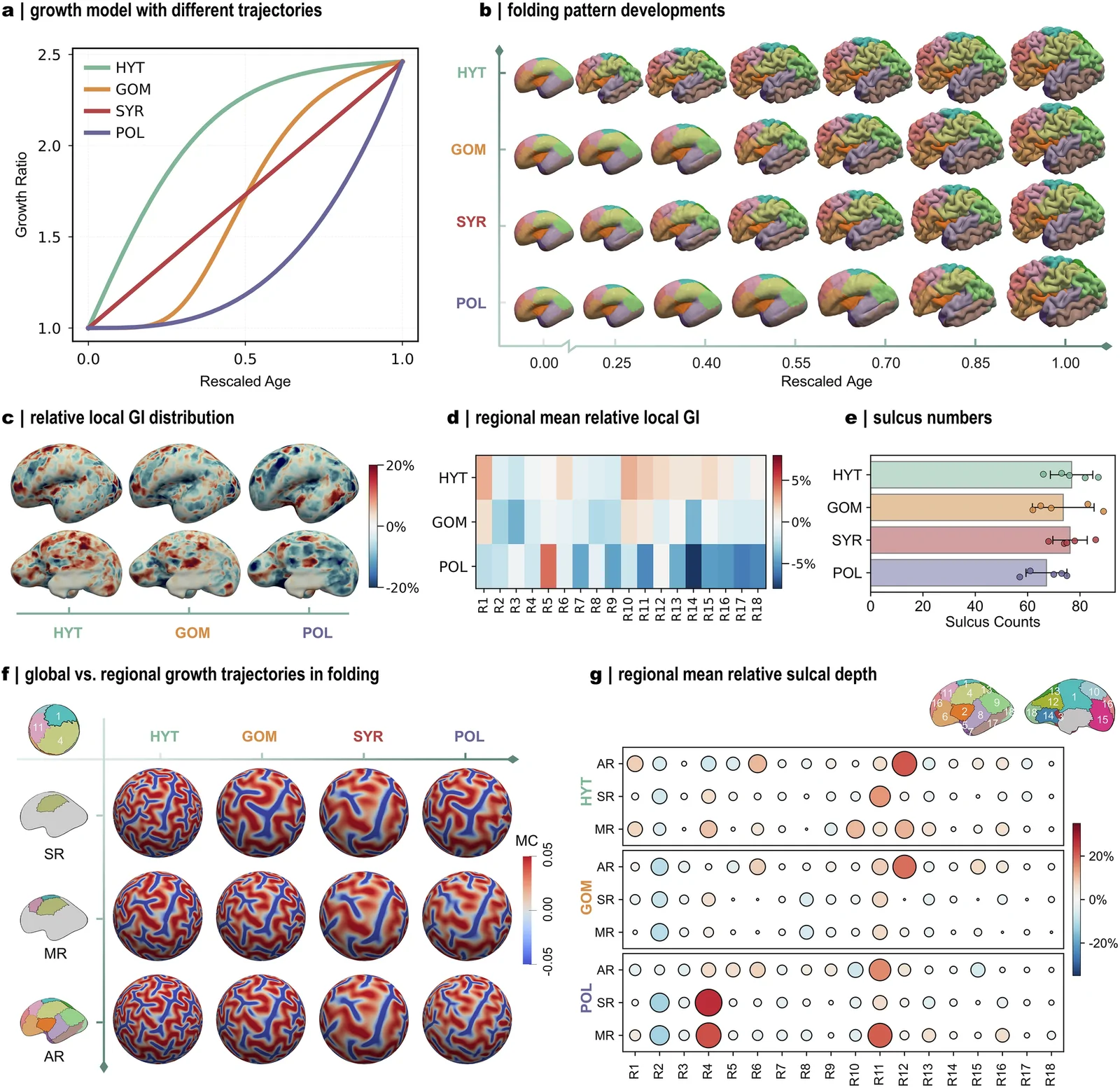
Regional growth trajectory modulates cortical morphology. **a** Growth models with identical final ratios but different temporal trajectories: hyperbolic tangent (HYT), Gompertz (GOM), symbolic regression characterization (SYR), and polynomial (POL). Region 4 is shown as an example. Trajectory variation is applied only to cortex tangential growth, while cortex radial growth and subcortex growth are fixed according to the model predictions shown in Fig. 2b and Supplementary Fig. 4. **b** Simulated folding development under different growth trajectory scenarios, with trajectory variations applied across all cortical regions. **c** Relative local gyrification index (GI) distributions at the final stage. SYR-based folding results are used as references, and all results are projected onto the initial brain geometry. **d** Average relative local GI across regions. **e** Number of sulci across growth models. Data are presented as mean values ± SD (*n* = 20; 5 subjects per case). **f** Regional vs global variation of growth trajectories. Cartoon illustrates perturbed trajectories for single region (SR), multiple adjacent regions (MR), and all cortical regions (AR). For regional variation, only selected regions deviated while others followed SYR. Folding patterns are shown with mean curvature (MC) mapped onto spherical projections. The same SYR result is displayed at three locations to facilitate visual comparison across panels. **g** Region-averaged relative sulcal depth across different trajectory and regional variation cases. Circle size denotes the magnitude of deviation from SYR, quantified as the relative error, and color denotes the direction of the difference. Source data are provided as a Source Data file.

Subsequently, we investigated the role of growth models in directing regional deformation during development (Fig. 6). This analysis is pertinent to developmental delays observed in neurodevelopmental disorders such as autism spectrum disorder^11^ and schizophrenia^12^, both linked to atypical cortical growth trajectories during prenatal stages. We specifically focused on the role of growth functions, considering two attributes: the temporal trajectories and final growth magnitudes. Given that thickness variation plays a relatively minor role in shaping major folding patterns, all modifications in this analysis were applied exclusively to the tangential component of cortical growth, while both radial cortical growth and subcortical growth were held unchanged.

To examine the effect of growth trajectories, we introduced three representative temporal profiles, namely the hyperbolic tangent (HYT), Gompertz distribution (GOM), and polynomials (POL) distributions, while maintaining identical end-point growth ratios (Fig. 6a). These trajectories were firstly applied globally across all cortical regions. Altering the growth trajectory introduces noticeable changes in the timing of fold emergence (Fig. 6b): the onset of folding varies among cases according to the temporal profile, whereas the final folding morphologies remain largely similar (Supplementary Movie 2). This behavior is governed by the time-dependent evolution of the relative cortex-to-subcortex growth ratio. With identical subcortical growth, varying cortical tangential trajectories alter how rapidly this relative ratio increases, thereby shifting the onset of folding, and leading to similar final morphologies due to matched end-point growth ratios. To quantify local differences, we calculated relative percentage errors in local GI with respect to the baseline trajectory (SYR) (Fig. 6c, d). Substantial deviations are observed for HYT and POL in both vertex-wise and regional comparisons, with HYT generally overestimating and POL underestimating local GI values, consistent with their growth divergence from the baseline growth trajectory. In contrast, the number of major sulci is comparable across all cases, though POL tends to yield slightly fewer sulci (Fig. 6e). These findings suggest that growth trajectories primarily modulate localized folding characteristics while having limited influence on major folding organization^25^. We then examined the effects of regionally varying growth trajectories. Two cases were considered: alteration within a single region (SR) and alteration across multiple adjacent regions (MR), with all other regions using the baseline trajectories. Folding morphologies were projected onto a spherical surface to visualize sulcal regions defined by negative mean curvature (Fig. 6f). Regional alterations produce distinct sulcal patterns compared to the global alteration case (AR), particularly under single-region modification. Quantitative analyses reveal that trajectory alterations influenced not only the perturbed regions, but also their immediate neighbors, while distant regions (e.g., R14: medial occipital, R17: middle and inferior temporal, and R18: lateral occipital) exhibit minimal changes in sulcal depth (Fig. 6g).

We further explored how the magnitude of cortical growth influences folding outcomes. To isolate this effect, we maintained identical linear trajectories for tangential growth across all regions while systematically varying the end-point growth ratios. Reducing the growth ratios by half, either regionally or globally, results in less developed folds within the affected regions, whereas unaffected areas remain largely unchanged (Supplementary Fig. 9a). Relative deviations again reveal that regional alterations propagate to neighboring areas (Supplementary Fig. 9b). In summary, these analyses indicate that growth ratio magnitude predominantly governs the overall development of major folding patterns, while growth trajectories modulate localized folding features.

Based on these observations, we investigated hemispherical symmetry in folding patterns, as the two hemispheres can differ in both geometry and growth profiles even within the same subject (Supplementary Fig. 10a). The model produces asymmetric folding patterns while preserving consistent major features across hemispheres (Supplementary Fig. 10b). Although measurable differences are detected in both folding morphology (Supplementary Fig. 10c) and regional measures (Supplementary Fig. 10d), these differences are not statistically significant (Supplementary Fig. 10e). Additionally, we explored the effects of varying subcortical growth, including changes in growth magnitude and regional heterogeneity (Supplementary Figs. 11, 12). These analyses show that variations in subcortical growth magnitude primarily influence brain volume and depth of sulci, while largely preserving the number and overall pattern of primary cortical folds (see “Effect of varying subcortical growth in modeling cortical folding” in Supplementary Materials for details). Together, these results establish the framework as a flexible platform for generating diverse folding patterns under varying parameters. This capability supports its use as a data-generating tool for AI-based prediction, a mechanistic framework for investigating cortical folding, and a translational model for studying abnormal brain development.

### Our modeling framework captures abnormal cortical folding in malformations

**Fig. 7:**
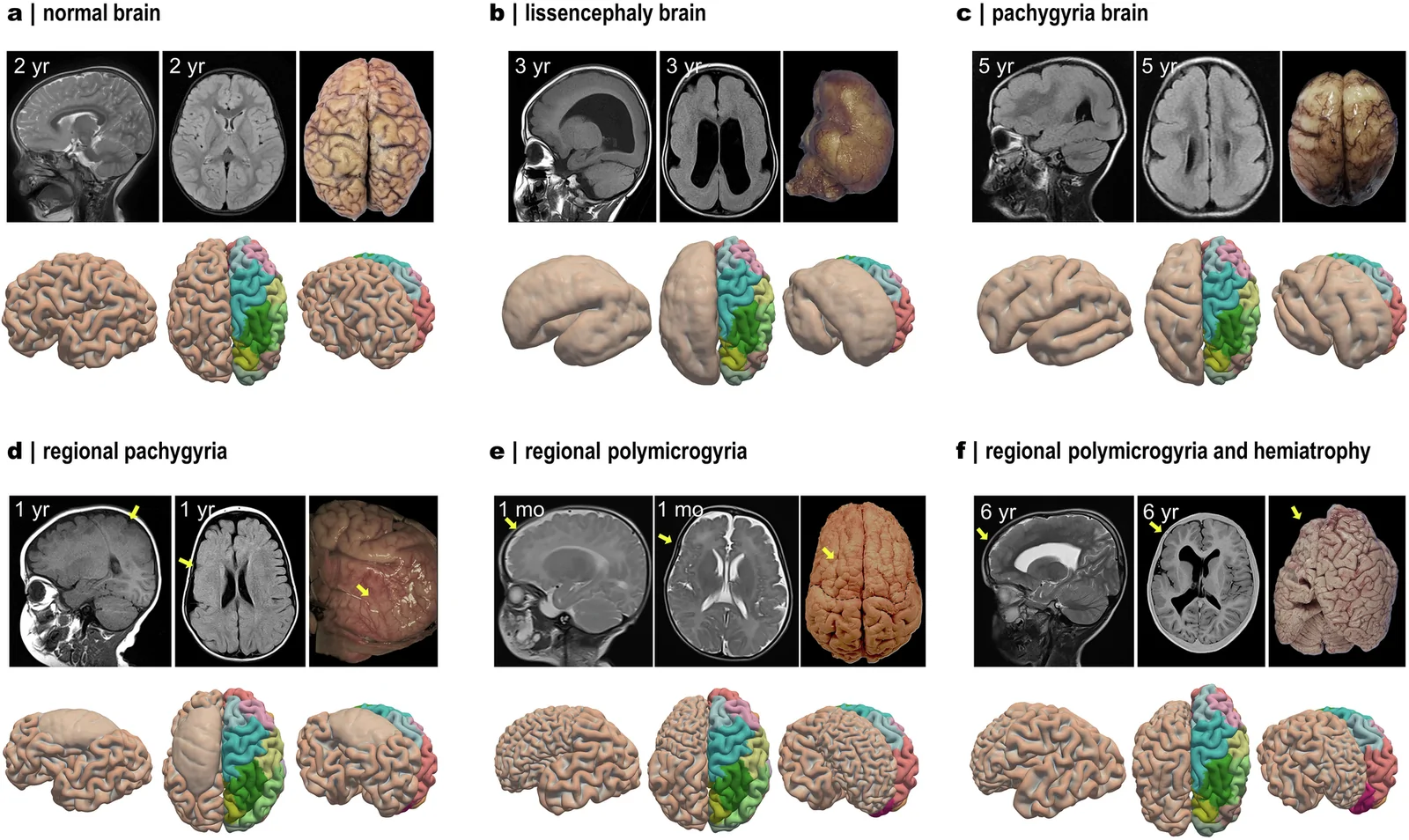
Modeling framework captures abnormal folding patterns in congenital brain malformations. Representative radiological and gross anatomical views of malformation cases are shown in panels **a**–**f** (top), alongside corresponding simulations generated with our whole-brain model (bottom). In each case, sagittal and axial MRI scans are provided together with gross anatomical photographs. **a** Normal developing brain at 2 years of age (MRI case courtesy of Dalia Ibrahim, Radiopaedia.org, rID: 212958; anatomy image adapted from ref.^72^ under the Creative Commons Attribution 4.0 International License (CC BY 4.0)). **b** Extreme lissencephaly brain at 3 years (MRI case courtesy of Ammar Haouimi, Radiopaedia.org, rID: 194766; anatomy image adapted from ref.^73^ under the CC BY 4.0 license). **c** Less severe lissencephaly (pachygyria) brain at 5 years (MRI case courtesy of Senai Goitom Sereke, Radiopaedia.org, rID: 181664; anatomy image adapted from ref.^74^ under the CC BY 4.0 license). **d** Regional pachygyria at 1 year (MRI case courtesy of Ammar Haouimi, Radiopaedia.org, rID: 67632; anatomy image adapted from ref.^75^ under the CC BY 4.0 license). **e** Regional polymicrogyria at 1 month (MRI case courtesy of Huda B. Gharbia, Radiopaedia.org, rID: 88483; anatomy image adapted from ref.^76^ under the CC BY 4.0 license). **f** Regional polymicrogyria with concomitant right hemispheric atrophy at 6 years (MRI case courtesy of Fazel Rahman Faizi, Radiopaedia.org, rID: 67168; anatomy image reproduced from ref.^77^ with permission from John Wiley and Sons; minor formatting adjustments were made for presentation). Simulated brains are shown in three orientations. A normal brain with regional parcellation is included for reference. All pathological regions are located in the right hemisphere, which is horizontally flipped to facilitate direct comparison with anatomical views. Yellow arrows indicate regions of malformation, with corresponding case descriptions provided in Supplementary Table 1.

In this section, we show that, by tuning folding-related parameters based on expert descriptions derived from MRI scans (Supplementary Table 1), the framework can qualitative reproduce abnormal folding phenotypes associated with common cortical malformations (Fig. 7). Representative examples include lissencephaly^44^ and polymicrogyria^45^, characterized by abnormally reduced or excessive cortical folding compared to normal morphology shown in Fig. 7a. For example, Fig. 7b illustrates a severe lissencephaly case with a smooth cortical surface and markedly increased initial cortical thickness. This phenotype is reproduced by doubling initial cortical thickness and reducing regional growth ratios to 30% of their baseline values in the normal case (Supplementary Movie 3). Similarly, pachygyria, a subtype of lissencephaly in which major sulcal landmarks are preserved despite an overall smoother surface, is recapitulated by applying the same increase in initial cortical thickness while maintaining the baseline regional growth model (Fig. 7c) (Supplementary Movie 4).

Beyond global malformations, the framework also captures region-specific developmental abnormalities. In one case, pachygyria localized to the right frontal and parietal lobes presents as a smooth cortical surface with reduced cortical volume (Fig. 7d). Although increasing cortical thickness in these regions, as applied in the global pachygyria case, generates smooth folding patterns, it fails to reproduce the locally reduced volume observed in this condition. Therefore, we selectively reduced the tangential growth ratios by half in the affected regions (R1: dorsal precentral & posterior cingulate, R4: lateral precentral & postcentral, and R11: caudal middle frontal), which effectively captures the reported abnormal folding phenotype (Supplementary Movie 5). Conversely, a regional polymicrogyria case, characterized by abnormally dense but shallow folds in the right frontal lobe (Fig. 7e), is reproduced by reducing initial cortical thickness by 40 % in selected frontal regions while keeping other parameters unchanged (Supplementary Movie 6). A second polymicrogyria case, accompanied by volume atrophy in the right prefrontal cortex (yellow arrow in Fig. 7f), is captured by reducing initial cortical thickness by 40 % in regions R6 (inferior frontal) and R16 (dorsal prefrontal) and halving both cortical and subcortical growth ratios (Supplementary Movie 7). Although these reproductions remain qualitative due to limited MRI data for constructing subject-specific simulation models and characterizing growth patterns, particularly the scarcity of longitudinal scans, the results demonstrate the framework’s ability to reproduce malformation-specific folding phenotypes and provide a physically interpretable link between growth dynamics and abnormal cortical morphology.

## Discussion

Cortical folds encode critical information about brain structural organization and functional specialization, yet their formation mechanisms remain poorly understood. While multiple factors, such as neuronal migration, axonal tension, and cranial constraints, have been implicated in shaping folding, mechanical forces arising from differential growth between the cortex and the subcortex are increasingly recognized as key drivers of cortical morphogenesis^41^. Previous computational efforts have provided valuable insight into this process, but often lacked anatomical fidelity and fell short of reproducing the spectrum of folding patterns observed in the developing human brain^21,27^. In this study, we introduced a whole-brain computational modeling framework that integrates heterogeneous, region-specific growth models with anatomically realistic prenatal brain geometries, thereby enabling faithful simulation of developmental folding trajectories documented in MRI datasets. The framework captures the spatiotemporal complexity of cortical morphogenesis, achieving anatomical fidelity in both the qualitative folding features and the quantitative morphometric measures.

Our findings demonstrate that tangential growth is the dominant driver of cortical folding, in agreement with both neuroimaging evidence and theoretical predictions^46^. Importantly, the inclusion of regional heterogeneity in tangential growth is essential for reproducing realistic folding morphologies. In the developing human brain, such heterogeneity is well documented and primarily arises from regionalized gene expression^47^ and molecular specialization^48^ across cortical areas. For example, spatial transcriptomic profiling has revealed that the prenatal cortex exhibits area- and layer-specific gene expression programs as early as mid-gestation, with frontal enrichment of genes such as CBLN2 and CYP26A1 and sharp transcriptional boundaries emerging between adjacent visual areas^7^. These spatially patterned molecular programs give rise to developmental heterogeneity across cortical regions, reflected in tissue-level organization such as axonal connectivity and vascular architecture, which together shape diverse folding morphologies^49^. Despite its biological relevance, growth heterogeneity has been underexplored in computational modeling of cortical folding. This lack of focus largely stems from the scarcity of high-quality MRI data and the inherent challenges of incorporating growth heterogeneity into anatomically realistic simulations^22^. Previous studies often relied on highly simplified geometries or imposed periodic growth fields on planar or patch-based models, limiting their ability to capture the complexity of cortical morphogenesis^25,50^. Such oversimplifications, while mathematically tractable, yield insights that are difficult to generalize to realistic developmental scenarios. Our framework directly addresses these limitations. By constructing anatomically faithful whole-brain models and MRI-based regional parcellations, we are able to assign growth laws derived directly from actual developmental trajectories into biophysical simulations. This approach provides a means to mechanistically examine how heterogeneous growth shapes cortical folding within a realistic developing brain. Notably, the framework offers a flexible platform that can be extended to incorporate other region-specific properties, such as spatial variations in material or cellular composition, provided reliable anatomical measurements are available. Beyond such extensions, it also enables mechanistic exploration of how intrinsic tissue properties, local growth dynamics, and emergent geometric constraints interact during cortical morphogenesis.

Both anatomical configuration and growth models critically shape cortical folding patterns. By systematically varying these factors, we showed that the framework can generate diverse yet realistic folding morphologies. This capability not only advances our understanding of the fundamental determinants of folding development but also, more importantly, provides a reliable platform for generating large-scale, high-fidelity synthetic brains with continuous developmental trajectories. Such synthetic datasets could serve supportive educational purposes, for instance, by providing intuitive visualizations of cortical morphogenesis and helping learners or practitioners better understand developmental concepts. Moreover, they can effectively supplement the limited availability of longitudinal brain imaging datasets by supplying anatomically faithful inputs for ML models that predict cortical folding development, a particularly valuable contribution given the computational cost of biophysical simulations. Recent advances in ML models, including GAN-based generative frameworks^51,52^, physics-informed neural networks (PINNs)^53^, transfer-learning approaches^54^, and sequence-learning architectures such as PointNet-LSTM^55^, have shown strong promise for capturing and forecasting cortical morphogenesis across gestation. Our framework provides biologically grounded synthetic data to train and validate these approaches. When extended to simulate patient-specific or atypical developmental patterns, it could generate abnormal developmental datasets to train predictive models for early detection and intervention in neurodevelopmental disorders.

Beyond the forward process of predicting folding patterns from initial brain morphologies, our modeling framework also suggests the potential for reverse inference, thus estimating early brain states from mature cortical configurations. Because the simulations are constrained by biologically informed growth dynamics and biophysical principles, they preserve surface topology and generate smooth geometric transitions, thereby maintaining a coherent developmental trajectory from fetal to adult stages. This continuity mirrors realistic longitudinal development and opens the possibility of reconstructing early anatomy from later morphologies. This strategy resonates with the broader concept of “reverse engineering the brain”^56^, here applied to cortical morphogenesis, where mature folding patterns are used to infer plausible early anatomical states. Although still speculative, this direction could yield extensive physically grounded representations of fetal brain morphology. Such synthetic datasets can alleviate the scarcity of fetal imaging and provide valuable context for studies of neonatal development and adult brain disorders rooted in early developmental deficits. Furthermore, this approach also holds promise for advancing evolutionary neuroscience, for example by informing the interpretation of fossil endocasts^57^ and the reconstruction of extinct or ancestral brain states^58^.

Our framework further demonstrates the ability to qualitatively replicate abnormal folding phenotypes, including lissencephaly, pachygyria, and polymicrogyria, by modifying growth laws or geometric parameters according to clinical case descriptions. These reproductions imply how malformation-specific perturbations give rise to distinct phenotypes. Clinically, such models could support early risk stratification and diagnosis by evaluating malformation progression from prenatal scans, offering a complementary tool for obstetric imaging and counseling. Beyond gross shape changes, certain conditions such as autism are also associated with localized variations in folding patterns^59^. While our framework does not directly capture such subtle cases, it provides a potential mechanical interpretation: alterations in growth trajectories, though not significantly affecting major folding architecture, may modulate local folding characteristics within affected regions and their neighbors. Furthermore, by disentangling the relative contributions of geometry and growth models, we found that primary folding patterns are more strongly governed by geometrical factors, including the initial cortical surface configuration and cortical thickness. In contrast, growth functions act more as dynamic modulators, influencing the magnitude and distribution of tissue deformation through their growth ratios.

While the present framework captures key aspects of cortical folding, several limitations also point toward opportunities for refinement. First, axonal fiber growth was not explicitly represented; its potential contribution was approximated through the imposed spatial growth heterogeneity. Yet, developmental processes such as axonal elongation^60^, pathfinding^61^, and fiber organization^62^ have been implicated in the emergence of cortical folds^41^. Second, subcortex was modeled as a homogeneous material with isotropic growth, without accounting for its internal anatomical structures. Although we explored the effects of heterogeneous subcortical growth and observed measurable influences on cortical folding patterns, the adopted parcellation does not represent true subcortical organization. Incorporating anatomically accurate subcortical structures in future models may further improve folding predictions^5^. Third, both the estimation of growth trajectories and subsequent analyses are performed based on parcellated regions; such regional averaging over relatively coarse parcellations may obscure fine-scale spatial heterogeneity present in real brains. In this study, the cortical regionalization map was adopted from a previously established large-scale developmental dataset rather than derived from the current cohort. This is because cortical regionalization requires large datasets with broad and continuous developmental coverage to reliably estimate vertex-wise developmental trajectories. Future studies with larger and more uniformly distributed datasets may enable the derivation of more robust, cohort-specific regionalization maps. Additionally, spatial variations in mechanical properties were not considered, despite their recognized influence on brain biomechanics^63^. Nonetheless, the framework is inherently extensible and can readily integrate such regional heterogeneities in growth and material composition, paving the way for increasingly comprehensive and biologically realistic models of cortical development.

Collectively, this work establishes an imaging-informed, whole-brain modeling framework that mechanistically links regional growth heterogeneity to cortical folding. By uniting population MRI with biophysical modeling, the framework offers a quantitative bridge between developmental imaging and physical theory to capture the spatiotemporal complexity of cortical morphogenesis. Beyond reproducing anatomically faithful folding patterns, it provides a quantitative and extensible platform for probing the developmental origins of neurodevelopmental disorders and for generating high-fidelity synthetic datasets that support data-driven neuroscience.

## Methods

### Brain imaging data

To characterize regional growth models, we assembled longitudinal measurements of brain surface area and cortical thickness from the developing Human Connectome Project (dHCP)^64^, which is a publicly available resource focused on early brain development. To ensure reconstruction and registration fidelity, we selected 120 scans (60 males, 60 females) aged 22-40 GWs to form the finalized study dataset. Sex differences were not explicitly examined, as the primary focus of this study is on broad developmental patterns. All images were processed with the infant dedicated computational pipeline, iBEAT V2^65^, to reconstruct high-quality cortical surfaces, followed by cross-subject surface registration in spherical space to ensure vertex-wise anatomical correspondence. For surface partitioning, we applied our previously developed developmental brain atlas^4^, which divides the cortex into 36 regions (18 per hemisphere). The parcellation was obtained using a data-driven framework based on non-negative matrix factorization (NMF), which groups cortical vertices according to shared developmental trajectories of surface area expansion^4^ (see “Cortical area regionalization” in the Supplementary Materials for details). Regional cortical surface area and thickness were quantified across gestation, and the resulting measurements served as the basis for further characterization of regional growth models.

### Symbolic regression for growth models characterization

We used symbolic regression to characterize regional growth models (see “Symbolic regression for growth models characterization” in the Supplementary Materials). To depict surface area expansion and cortical thickness variation, we decomposed cortical growth into two independent modes: tangential (in-plane) growth and radial (out-of-plane) growth. For computational implementation, the data were converted into unitless growth ratios and a virtual time variable using the following formulas:

1 $${g}_{t}\left(t\right)=\sqrt{S\left(t\right)/{S}_{0}},\,\,\,\,{g}_{r}\left(t\right)=T\left(t\right)/{T}_{0},\,\,\,\,t=\frac{{GA}\left(t\right)-G{A}_{0}}{G{A}_{\max }-G{A}_{0}}$$

where ${g}_{t}(t)$ and ${g}_{r}(t)$ denote tangential and radial growth ratio, respectively; ${S}_{0}$ and ${T}_{0}$ are the initial surface area and cortical thickness measured at 22 GWs ($G{A}_{0}$); and $G{A}_{\max }$ corresponds to the maximum gestational age in the dataset (40 GWs). After conversion, $t\in ({{\mathrm{0,1}}})$ serves as the single input variable for the symbolic regression algorithm to identify the optimal growth function $g\left(t\right)$ for each region (Supplementary Fig. 1).

### Biophysics in modeling brain folding

Cortical folding arises from complex biological processes, but from a mechanical perspective it can be approximated as a buckling and post-buckling problem. To capture growth-driven folding in the developing brain, we adopt the well-established differential growth theory, which attributes folding to deformation mismatch between the rapidly expanding cortical layer and the more slowly growing subcortical layer^66^. The deformation kinematics can be described within the framework of continuum mechanics. Firstly, we consider a one-to-one mapping between the undeformed and deformed continuum body, denoted as ${{{\boldsymbol{x}}}}=\varphi \left(X,t\right)$, which carries a material particle ${{{\boldsymbol{X}}}}$ in the reference configuration ${{{{\mathscr{B}}}}}_{0}$ to its position ${{{\boldsymbol{x}}}}$ in the current configuration ${{{{\mathscr{B}}}}}_{t}$. This transformation is described by the deformation gradient, ${{{\boldsymbol{F}}}}={\nabla }_{{{{\bf{X}}}}}\varphi \left(X,t\right)$. Following the principle of multiplicative decomposition, the deformation process can be interpreted as a combination of stress-free growth and elastic accommodation. Accordingly, both the deformation gradient ${{{\boldsymbol{F}}}}$ and Jacobian determinant J, which quantifies volumetric change, are decomposed into elastic and growth components:

2 $${{{\boldsymbol{F}}}}={{{{\boldsymbol{F}}}}}^{e}\cdot {{{{\boldsymbol{F}}}}}^{g},\,\,\,\,\, J=\det {{{\boldsymbol{F}}}}={J}^{{{{\rm{e}}}}}\cdot {J}^{g}.$$

For the growth component ${{{{\boldsymbol{F}}}}}^{g}$, we modeled cortical growth as orthotropic, comprising both the in-plane surface expansion and out-of-plane thickness variation:

3 $${{{{\boldsymbol{F}}}}}^{g}={g}_{t}{{{\boldsymbol{I}}}}+\left({g}_{r}-{g}_{t}\right)\hat{{{{\boldsymbol{n}}}}}\otimes \hat{{{{\boldsymbol{n}}}}},\,\,{J}^{g}=\det {{{{\boldsymbol{F}}}}}^{g}$$

where ${g}_{t}$ and ${g}_{r}$ are the tangential (in-plane) and radial (out-of-plane) growth ratios, respectively, with their expressions ${g}_{t}={g}_{t}(t)$ and ${g}_{r}={g}_{r}(t)$ derived from symbolic regression. For subcortex, we assume isotropic growth (${g}_{t}={g}_{r}$), implying uniform expansion in all directions, a simplification widely supported in the literature^10,23,25^. The vector $\hat{{{{\boldsymbol{n}}}}}$ denotes the local surface normal, oriented outward in the radial direction.

For the elastic component ${{{{\boldsymbol{F}}}}}^{e}$, we followed previous brain-folding models and adopted a standard neo-Hookean hyperelastic model to describe the constitutive behavior of both the cortex and the subcortex^19^. The strain energy density function is expressed in terms of the elastic deformation gradient ${{{{\boldsymbol{F}}}}}^{e}$ and its Jacobian ${J}^{{{{\rm{e}}}}}$,

4 $$W\left({{{{\boldsymbol{F}}}}}^{e}\right)=\frac{1}{2}K{{{\mathrm{ln}}}}^{2}\left({J}^{e}\right)+\frac{1}{2}\mu \left({{{{\boldsymbol{F}}}}}^{e}:{{{{\boldsymbol{F}}}}}^{e}-2{{\mathrm{ln}}}{J}^{e}-3\right),$$

where K and μ are bulk and shear moduli, respectively. From thermodynamic principles, the first Piola-Kirchhoff stress ${{{\boldsymbol{P}}}}$ is work-conjugate to the deformation gradient ${{{\boldsymbol{F}}}}$, and specially related to ${{{{\boldsymbol{F}}}}}^{e}$:

5 $${{{\boldsymbol{P}}}}=\frac{\partial W}{\partial {{{\boldsymbol{F}}}}}=\frac{\partial W\left({{{{\boldsymbol{F}}}}}^{e}\right)}{\partial {{{{\boldsymbol{F}}}}}^{e}}:\frac{\partial {{{{\boldsymbol{F}}}}}^{e}}{\partial {{{\boldsymbol{F}}}}}={{{{\boldsymbol{P}}}}}^{e}\cdot {{{{\boldsymbol{F}}}}}^{g-T}.$$

The Piola stress enters the standard balance of linear momentum, which governs mechanical equilibrium. In the absence of body forces, this reduces to the vanishing divergence of the first Piola-Kirchhoff stress:

6 $${{{\rm{Div}}}}\,\,{{{\boldsymbol{P}}}}=0.$$

In summary, cortical and subcortical growth defines a stress-free configuration, which must be balanced by the elastic deformation arising from material confinement. The neo-Hookean model provides this elastic response, ensuring mechanical compatibility and preventing unphysical deformations. Although brain tissue is viscoelastic, cortical folding during prenatal development occurs over relatively long timescales and can often be approximated as quasi-static; accordingly, hyperelastic formulations are widely used in computational models^10,23,25,35,50^. To further assess this assumption, we examined the effects of viscoelasticity and found that folding patterns are sensitive to variations in viscoelastic parameters (Supplementary Fig. 13; see “Effect of stress-dependent growth in modeling cortical folding” in the Supplementary Materials).

### Computational model of the developing brain

To realistically model cortical folding in the prenatal brains, we used reconstructed cortical surfaces at 22 GWs as the initial simulation geometry. As shown in Supplementary Fig. 3a, the cortical and subcortical surfaces were registered and parcellated according to the developmental atlas. Solid models were reconstructed, merged, and meshed to produce a high-quality hexahedral volume. A mesh size of 0.4 mm was used, resulting in ~1,600,000 hexahedral elements for a whole-brain model. As seen in Supplementary Fig. 3b, interior elements formed uniform voxels, while boundary elements were split, reprojected, and smoothed to preserve surface conformity. Elements on both sides of the cortex-subcortex interface shared faces and nodes, ensuring geometric and mechanical continuity. This meshing strategy ensured at least five elements across cortical thickness, providing sufficient resolution to capture folding development in finite element simulation^23^. Mesh quality was evaluated using scaled Jacobian and edge ratio metrics, with most elements close to 1, confirming that the mesh satisfied the requirements for reliable simulation (Supplementary Fig. 3c). After meshing, cortical tissue elements were subdivided into 19 regions according to cortical surface parcellation for implementing regional growth in the modeling (Supplementary Fig. 3d). To maintain consistency with anatomical parcellation, the regional convoluted surface was constructed from cortical and subcortical surfaces sharing the same regional index, and enclosed elements were assigned to the corresponding region. Elements near regional interfaces were allocated to the adjacent region with the higher index. Furthermore, orthotropic growth was enforced by assigning each cortical tissue element a material orientation, calculated as the normal vector from the element centroid to the nearest vertex on the cortical surface (Supplementary Fig. 3e).

Brain growth was simulated as thermal expansion in ABAQUS 2023 (Dassault Systems, Paris, France) with the dynamic-explicit solver, considering the analogy between the volumetric growth and thermal expansion^67^. Both the cortex and the subcortex were modeled as nearly incompressible neo-Hookean materials, with comparable shear moduli of 0.65 kPa and 0.68 kPa^68^. Regional growth models ${g}_{r}\left(t\right)$ and ${g}_{t}\left(t\right)$, derived from symbolic regression, were applied to the cortex through a user-defined subroutine *VUEXPAN*. Following previous studies, we applied a sigmoid shape growth profile across cortical depth to reflect the layered growth patterns observed in cortical development^6,7^ (Supplementary Fig. 3f). In contrast, the subcortex was modeled using an isotropic growth model that captures its volumetric changes during normal brain development (Supplementary Fig. 4). Free boundary conditions were imposed on the cortical surface, together with a self-contact constraint to prevent self-penetration. A uniform pressure was applied to represent the mechanical confinement of the meninges and developing skull^23^. The total simulation time was set to the maximal rescale gestational time (t=1). To avoid computational instabilities, the maximum time step was set as $\triangle t=0.05a\sqrt{\rho /K}$, where a is the average mesh size, ρ is the mass density, K is bulk modulus^25^. Detailed mechanical parameters are provided in Supplementary Table. 2. All simulations were performed on a Dell Alienware desktop equipped with an Intel® Core™ Ultra 9 285 K processor (24 cores) @ 3.7 GHz, and 64 GB of RAM. For a single subject, the simulation of brain development from 22 to 40 GWs required ~12 h of computation.

### Postprocessing analyses and quantitative metrics

In total, we simulated folding development for 10 brains at 22 GWs. Simulation output files were exported and analyzed with in-house postprocessing codes. At selected simulation stages, node coordinates were extracted to reconstruct the cortical surfaces. For regional analysis, alignment between simulated and real brain parcellations was achieved by matching vertex positions using multimodal surface matching^2^ between the undeformed simulated surface and the real brain geometry at 22 GWs. The assigned regional indices were directly propagated to subsequent deformed states, as the simulation preserved the original cortical surface connectivity and maintained geometry without distortion. Notably, the framework allows cortical surfaces to be reconstructed at any simulation moment, allowing continuous tracking of folding progression that cannot be fully captured by longitudinal imaging. From the reconstructed cortical surfaces, multiple quantitative metrics were calculated to evaluate the accuracy of folding simulations. These include global and local gyrification index, major sulcus count, sulcal depth, curvature measures, enabling systematic comparisons at whole-brain, regional, and vertex levels. For their definitions, please see “Quantitative metrics definition” in the Supplementary Materials for details.

The sulcal landmarks in Fig. 4 were manually extracted and visualized using ParaView 5.13.0-RC2. We focused on six major sulci, including central sulcus, postcentral sulcus, intraparietal sulcus, superior temporal sulcus, lateral sulcus, and inferior frontal sulcus, which were selected for their anatomical prominence and accessibility of extraction. These sulci also carry important functional relevance, as they delineate boundaries of primary sensorimotor cortices (central and postcentral sulcus), visuospatial and attentional networks (intraparietal sulcus), auditory and language-related regions (superior temporal and lateral sulcus), and higher-order executive areas (inferior frontal sulcus). Although the alignment is not restricted to these six sulci, a more detailed landmark alignment analysis lies beyond the scope of this study. Here, we highlight these six representative sulci to demonstrate the capability of our whole-brain framework to model brain morphology with anatomical fidelity.

After surface registration, the similarity s between distributions of a quantitative metric d in real brains (${S}_{{{{\rm{real}}}}}$) and simulated brains (${S}_{{{{\rm{simu}}}}}$) was evaluated using the following formula^40^:

7 $$s\left({S}_{{{{\rm{real}}}}},{S}_{{{{\rm{simu}}}}}\right)=1-\frac{1}{2\sqrt{N}}{{||}{d}_{{{{\rm{real}}}}}-{d}_{{{{\rm{simu}}}}}\circ {f}_{{{{\rm{sr}}}}}{||}}_{2},$$

where N is the total number of vertices; ${f}_{{{{\rm{sr}}}}}$ is the mapping from the simulated brain to the real brain, accommodating the higher vertex density of the real mesh; ${{||}\cdot {||}}_{2}$ denotes Euclidean norm, i.e., ${{||}{d}_{{{{\rm{real}}}}}-{d}_{{{{\rm{simu}}}}}\circ {f}_{{{{\rm{sr}}}}}{||}}_{2}={\left({\sum }_{i=1}^{N}{\left({d}_{{{{\rm{real}}}}}-{d}_{{{{\rm{simu}}}}}\circ {f}_{{{{\rm{sr}}}}}\right)}^{2}\right)}^{1/2}$. Noted, to ensure the similarity measure always falls within the range of [0,1], the quantitative metric d must be normalized to [-1,1], with a higher value of s indicating greater similarity^40^. In this study, we calculate the similarity based on the distribution of the shape index, which inherently lies within the required range. By contrast, the similarity score used in Fig. 3c was defined as $\exp (-|{{\mathrm{ln}}}({d}_{{{{\rm{simu}}}}}/{d}_{{{{\rm{real}}}}})|),$ where ${d}_{{{{\rm{simu}}}}}$ and ${d}_{{{{\rm{real}}}}}$ denotes the metric measure of simulated and real brain cases, respectively. This similarity score has values between 0 and 1, with 1 indicating perfect agreement. The metric deviation in Fig. 5b is defined as $({d}_{{{{\rm{stu}}}}}-{d}_{{{{\rm{ref}}}}})/{d}_{{{{\rm{ref}}}}}$, whereas the relative percentage errors in Fig. 6c-d is defined as $({d}_{{{{\rm{stu}}}}}-{d}_{{{{\rm{ref}}}}})/{d}_{{{{\rm{ref}}}}} * 100\%$, where ${d}_{{{{\rm{stu}}}}}$ and ${d}_{{{{\rm{ref}}}}}$ denotes the metric values for the studied and reference brain case, respectively.

To investigate the influence of growth trajectories on cortical folding simulation, we introduced three additional growth models based on a high-order polynomial (POL), hyperbolic tangent (HYT), and Gompertz distribution (GOM), respectively (Fig. 6a). These functional forms have been used in modeling tissue growth processes such as tumor progression^69^ and the development of myelinated white matter in the human brain^70^. The growth functions are defined as:

8 $${g}_{{{{\rm{POL}}}}}\left(t\right)={c}_{1} * {t}^{{c}_{2}},$$

9 $${g}_{{{{\rm{HYT}}}}}\left(t\right)={c}_{3} * \tanh \left({c}_{4} * t\right)+1,$$

10 $${g}_{{{{\rm{GOM}}}}}\left(t\right)={c}_{5} * \exp \left(-\exp \left(-{c}_{6} * \left(t-{c}_{7}\right)\right)\right)+1,$$

where ${c}_{i}$ are constants. As an illustrative case, we considered the tangential growth model of region 1 (Fig. 6a), for which the symbolic regression (SYR) predicted model is given by ${g}_{{{{\rm{SYR}}}}}\left(t\right)=1.46 * t+1$. Accordingly, the parameter values were set as: POL, ${c}_{1}=1.46,{c}_{2}=3$, HYT, ${c}_{3}=1.46 * 1.01,{c}_{4}=2.6$, and GOM, ${c}_{5}=1.46 * 1.04,{c}_{6}=6,{c}_{7}=0.45$. The parameter values were empirically assigned to ensure that all models converge to a consistent growth ratio at the final stage, thereby eliminating differences in absolute growth magnitude and allowing the analysis to focus exclusively on the effect of growth trajectories.

For the growth scenarios shown in Fig. 3, the isotropic growth with a uniform profile (IGU) case was defined by linear growth functions ${g}_{r}\left(t\right)={g}_{t}\left(t\right)=\sqrt{8} * t+1$, whereas the tangential growth with a uniform profile (TGU) case used ${g}_{r}\left(t\right)=1,{g}_{t}\left(t\right)=\sqrt{8} * t+1$. The chosen growth ratio values follow those commonly adopted in previous studies for modeling simplification^10,25^.

### Statistical analysis

Statistical analyses were performed using non-parametric tests given the small sample size (*n* = 10 per group), although data satisfied normality as assessed by the Shapiro-Wilk test. In Fig. 3d, difference in the sulcus counts among five growth conditions (Real, OGR, TGR, TGU, and IGU; *n* = 50, 10 per group) were first evaluated using the Kruskal–Wallis test to evaluate the null hypothesis that group medians were equal. When the overall test was significant (*P* < 0.05), post hoc pairwise comparisons were carried out using Dunn’s test with Bonferroni correction for multiple testing. In Fig. 5b, paired comparisons of regional mean sulcal depth between real and simulated brains were evaluated using the two-sided Wilcoxon signed-rank test (*n* = 20, 10 per case). In Supplementary Fig. 10e, paired comparisons of sulcal counts between left and right hemispheres (*n* = 20, 10 per hemisphere) were also assessed using the two-sided Wilcoxon signed-rank test. The null hypothesis was that the median differences between pairs was zero. All tests were two-tailed, with *P* < 0.05 considered statistically significant. Statistical analyses were performed using Origin 2025.

### Reporting summary

Further information on research design is available in the Nature Portfolio Reporting Summary linked to this article.

## Supplementary information

Supplementary Information

Description of Additional Supplementary Files

Supplementary Movie 1

Supplementary Movie 2

Supplementary Movie 3

Supplementary Movie 4

Supplementary Movie 5

Supplementary Movie 6

Supplementary Movie 7

Reporting Summary

Transparent Peer Review file

## Source data

Source Data

## Data Availability

The data supporting these findings, including model files and the regional surface area and cortical thickness data used for growth model characterization have been deposited in the Figshare repository^71^ and are available at (10.6084/m9.figshare.31866178). The MRI data obtained from the dHCP dataset are publicly available through the Developing Human Connectome Project repository at (https://www.developingconnectome.org). Source data are provided with this paper.

## Code availability

The code supporting this study, including scripts for growth model characterization, computational model construction, simulation postprocessing, data analysis and figure creation, is available on GitHub at (https://github.com/BioDMX-UGA/BiophysicalCorticalMorphogenesis).
