# Supplementary material for: Biophysical modeling of anatomically realistic prenatal cortical folding development
Source: Nat Commun. 2026 Jul 16;17:8711. doi: 10.1038/s41467-026-75582-9 (PMC13490475; doi:10.1038/s41467-026-75582-9)
Supplement: Supplementary file 1 [file 41467_2026_75582_MOESM10_ESM.pdf]

nature portfolio

Corresponding author(s): Xianqiao Wang

Last updated by author(s): Mar 26, 2026

Reporting Summary

Nature Portfolio wishes to improve the reproducibility of the work that we publish. This form provides structure for consistency and transparency in reporting. For further information on Nature Portfolio policies, see our Editorial Policies and the Editorial Policy Checklist.

Please do not complete any field with "not applicable" or n/a. Refer to the help text for what text to use if an item is not relevant to your study.
For final submission: please carefully check your responses for accuracy; you will not be able to make changes later.

Statistics

For all statistical analyses, confirm that the following items are present in the figure legend, table legend, main text, or Methods section.

n/a	Confirmed	
☐	☒	The exact sample size (*n*) for each experimental group/condition, given as a discrete number and unit of measurement
☐	☒	A statement on whether measurements were taken from distinct samples or whether the same sample was measured repeatedly
☐	☒	The statistical test(s) used AND whether they are one- or two-sided<br>*Only common tests should be described solely by name; describe more complex techniques in the Methods section.*
☒	☐	A description of all covariates tested
☐	☒	A description of any assumptions or corrections, such as tests of normality and adjustment for multiple comparisons
☐	☒	A full description of the statistical parameters including central tendency (e.g. means) or other basic estimates (e.g. regression coefficient) AND variation (e.g. standard deviation) or associated estimates of uncertainty (e.g. confidence intervals)
☐	☒	For null hypothesis testing, the test statistic (e.g. *F*, *t*, *r*) with confidence intervals, effect sizes, degrees of freedom and *P* value noted<br>*Give P values as exact values whenever suitable.*
☒	☐	For Bayesian analysis, information on the choice of priors and Markov chain Monte Carlo settings
☒	☐	For hierarchical and complex designs, identification of the appropriate level for tests and full reporting of outcomes
☒	☐	Estimates of effect sizes (e.g. Cohen's *d*, Pearson's *r*), indicating how they were calculated

Our web collection on statistics for biologists contains articles on many of the points above.

Software and code

Policy information about availability of computer code

Data collection	Abaqus 2023, User-defined subroutine code, ParaView 5.13.0-RC2, Matlab R2024a
Data analysis	Matlab R2024a, Python, Origin 2025

For manuscripts utilizing custom algorithms or software that are central to the research but not yet described in published literature, software must be made available to editors and reviewers. We strongly encourage code deposition in a community repository (e.g. GitHub). See the Nature Portfolio guidelines for submitting code & software for further information.

Data

Policy information about availability of data

All manuscripts must include a data availability statement. This statement should provide the following information, where applicable:
- Accession codes, unique identifiers, or web links for publicly available datasets
- A description of any restrictions on data availability
- For clinical datasets or third party data, please ensure that the statement adheres to our policy

https://github.com/BioDMX-UGA/BiophysicalCorticalMorphogenesis

Data Availability Statement

The data supporting these findings, including model files and the regional surface area and cortical thickness data used for growth model characterization have been deposited in the Figshare repository and are available at https://doi.org/10.6084/m9.figshare.31866178. The MRI data obtained from the dHCP dataset are publicly available through the Developing Human Connectome Project repository at https://www.developingconnectome.org. Source data are provided with this paper.

Code Availability Statement

The code supporting this study, including scripts for growth model characterization, computational model construction, simulation postprocessing, data analysis and figure creation, is available on GitHub at https://github.com/BioDMX-UGA/BiophysicalCorticalMorphogenesis.

Research involving human participants, their data, or biological material

Policy information about studies with human participants or human data. See also policy information about sex, gender (identity/presentation), and sexual orientation and race, ethnicity and racism.

Reporting on sex and gender	A subset of 120 scans spanning 22-40 GWs, comprising 60 males and 60 females, was selected for analysis. Sex differences were not explicitly examined, as the primary focus of this study is on broad developmental patterns.
Reporting on race, ethnicity, or other socially relevant groupings	The dHCP dataset comprises participants from diverse backgrounds. In this study, race, ethnicity, and other socially relevant groupings were not explicitly considered, as they were not the focus of the analysis.
Population characteristics	In this study, the structural MRI data were obtained from the developing Human Connectome Project (dHCP). The cohort included 120 scans spanning 22–40 gestational weeks (60 males, 60 females).
Recruitment	Participant were recruited as part of the developing Human connectome Project (dHCP)
Ethics oversight	This study used publicly available data from the developing Human Connectome Project (dHCP). Ethical approval and informed consent were obtained by the original study in accordance with institutional guidelines. No additional ethical approval was required for this secondary analysis

Note that full information on the approval of the study protocol must also be provided in the manuscript.

Field-specific reporting

Please select the one below that is the best fit for your research. If you are not sure, read the appropriate sections before making your selection.

☒ Life sciences ☐ Behavioural & social sciences ☐ Ecological, evolutionary & environmental sciences

For a reference copy of the document with all sections, see nature.com/documents/nr-reporting-summary-flat.pdf

Life sciences study design

All studies must disclose on these points even when the disclosure is negative.

Sample size	The sample size (n = 120) was determined based on data availability and quality criteria from the developing Human Connectome Project (dHCP). Scans were selected to ensure high-quality reconstruction and registration across the developmental window of 22-40 GWs.
Data exclusions	Data exclusion was based on quality control criteria. Scans with incomplete data or insufficient reconstruction or registration quality were excluded. No additional exclusion criteria were applied
Replication	Analyses were performed on a single cohort (n = 120) from the dHCP. No independent replication cohort was used
Randomization	No randomization was performed, as this was an observational study based on pre-existing data from the dHCP.
Blinding	No blinding was performed, as analyses were conducted on pre-existing, de-identified data from the dHCP using automated processing pipelines.

Behavioural & social sciences study design

All studies must disclose on these points even when the disclosure is negative.

Study description	
Research sample	
Sampling strategy	
Data collection	
Timing	
Data exclusions	
Non-participation	
Randomization	

Ecological, evolutionary & environmental sciences study design

All studies must disclose on these points even when the disclosure is negative.

Study description

Research sample

Sampling strategy

Data collection

Timing and spatial scale

Data exclusions

Reproducibility

Randomization

Blinding

Did the study involve field work? ☐ Yes ☒ No

Field work, collection and transport

Field conditions

Location

Access & import/export

Disturbance

Reporting for specific materials, systems and methods

We require information from authors about some types of materials, experimental systems and methods used in many studies. Here, indicate whether each material, system or method listed is relevant to your study. If you are not sure if a list item applies to your research, read the appropriate section before selecting a response.

Materials & experimental systems

n/a	Involved in the study
☒	☐ Antibodies
☒	☐ Eukaryotic cell lines
☒	☐ Palaeontology and archaeology
☒	☐ Animals and other organisms
☒	☐ Clinical data
☒	☐ Dual use research of concern
☒	☐ Plants

Methods

n/a	Involved in the study
☒	☐ ChIP-seq
☒	☐ Flow cytometry
☐	☒ MRI-based neuroimaging

Antibodies

Antibodies used

Validation

Eukaryotic cell lines

Policy information about cell lines and Sex and Gender in Research

Cell line source(s)

Authentication

Mycoplasma contamination

Commonly misidentified lines
(See ICLAC register)

Palaeontology and Archaeology

Specimen provenance

Specimen deposition

Dating methods

☐ Tick this box to confirm that the raw and calibrated dates are available in the paper or in Supplementary Information.

Ethics oversight

Note that full information on the approval of the study protocol must also be provided in the manuscript.

Animals and other research organisms

Policy information about studies involving animals; ARRIVE guidelines recommended for reporting animal research, and Sex and Gender in Research

Laboratory animals

Wild animals

Reporting on sex

Field-collected samples

Ethics oversight

Note that full information on the approval of the study protocol must also be provided in the manuscript.

Clinical data

Policy information about clinical studies

All manuscripts should comply with the ICMJE guidelines for publication of clinical research and a completed CONSORT checklist must be included with all submissions.

Clinical trial registration

Study protocol

Data collection

Outcomes

Dual use research of concern

Policy information about dual use research of concern

Hazards

Could the accidental, deliberate or reckless misuse of agents or technologies generated in the work, or the application of information presented in the manuscript, pose a threat to:

No | Yes
- [ ] [ ] Public health
- [ ] [ ] National security
- [ ] [ ] Crops and/or livestock
- [ ] [ ] Ecosystems
- [ ] [ ] Any other significant area

Experiments of concern

Does the work involve any of these experiments of concern:

No | Yes
- [ ] [ ] Demonstrate how to render a vaccine ineffective
- [ ] [ ] Confer resistance to therapeutically useful antibiotics or antiviral agents
- [ ] [ ] Enhance the virulence of a pathogen or render a nonpathogen virulent
- [ ] [ ] Increase transmissibility of a pathogen
- [ ] [ ] Alter the host range of a pathogen
- [ ] [ ] Enable evasion of diagnostic/detection modalities
- [ ] [ ] Enable the weaponization of a biological agent or toxin
- [ ] [ ] Any other potentially harmful combination of experiments and agents

Plants

Seed stocks

Novel plant genotypes

Authentication

ChIP-seq

Data deposition

- [ ] Confirm that both raw and final processed data have been deposited in a public database such as GEO.
- [ ] Confirm that you have deposited or provided access to graph files (e.g. BED files) for the called peaks.

Data access links
May remain private before publication.

Files in database submission

Genome browser session
(e.g. UCSC)

Methodology

Replicates

Sequencing depth

Antibodies

Peak calling parameters

Data quality

Software

Flow Cytometry

Plots

Confirm that:

☐ The axis labels state the marker and fluorochrome used (e.g. CD4-FITC).

☐ The axis scales are clearly visible. Include numbers along axes only for bottom left plot of group (a 'group' is an analysis of identical markers).

☐ All plots are contour plots with outliers or pseudocolor plots.

☐ A numerical value for number of cells or percentage (with statistics) is provided.

Methodology

Sample preparation

Instrument

Software

Cell population abundance

Gating strategy

☐ Tick this box to confirm that a figure exemplifying the gating strategy is provided in the Supplementary Information.

Magnetic resonance imaging

Experimental design

Design type	Observational study using secondary analysis of imaging data
Design specifications	Cross-sectional analysis of fetal/neonatal brain MRI data across 22–40 gestational weeks
Behavioral performance measures	Not applicable
Imaging type(s)	Structural MRI (T1-weighted and T2-weighted)
Field strength	3 Tesla
Sequence & imaging parameters	High-resolution T1w and T2w structural images acquired using neonatal-optimized protocols (see developing Human Connectome Project for details)
Area of acquisition	Whole brain

Diffusion MRI ☐ Used ☒ Not used

Preprocessing

Preprocessing software	iBEAT V2.0 for cortical surface reconstruction, followed by in-house MATLAB-based processing
Normalization	Surface-based normalization via spherical registration to establish vertex-wise correspondence across subjects.
Normalization template	UNC Neonate Cortical Surface Atlases
Noise and artifact removal	Performed within the iBEAT pipeline, including bias field correction and image quality control during reconstruction
Volume censoring	Not applicable

Statistical modeling & inference

Model type and settings	Non-parametric statistical analysis. Group differences were assessed using Kruskal–Wallis tests with Dunn's post hoc comparisons (Bonferroni correction). Paired comparisons were evaluated using Wilcoxon signed-rank tests. All tests were two-tailed with significance set at $P < 0.05$.
Effect(s) tested	Differences in sulcal counts across growth conditions, paired differences in regional sulcal depth between real and simulated brains, and hemispheric differences in sulcal counts

Specify type of analysis: ☐ Whole brain ☐ ROI-based ☒ Both

Statistic type for inference (See Eklund et al. 2016): Non-parametric tests (Kruskal–Wallis and Wilcoxon signed-rank), two-tailed

Correction: Bonferroni correction for multiple comparisons

Models & analysis

n/a	Involved in the study
☒	☐ Functional and/or effective connectivity
☒	☐ Graph analysis
☒	☐ Multivariate modeling or predictive analysis

Functional and/or effective connectivity

Graph analysis

Multivariate modeling and predictive analysis

This checklist template is licensed under a Creative Commons Attribution 4.0 International License, which permits use, sharing, adaptation, distribution and reproduction in any medium or format, as long as you give appropriate credit to the original author(s) and the source, provide a link to the Creative Commons license, and indicate if changes were made. The images or other third party material in this article are included in the article's Creative Commons license, unless indicated otherwise in a credit line to the material. If material is not included in the article's Creative Commons license and your intended use is not permitted by statutory regulation or exceeds the permitted use, you will need to obtain permission directly from the copyright holder. To view a copy of this license, visit http://creativecommons.org/licenses/by/4.0/
