# Supplementary material for: Biophysical modeling of anatomically realistic prenatal cortical folding development
Source: Nat Commun. 2026 Jul 16;17:8711. doi: 10.1038/s41467-026-75582-9 (PMC13490475; doi:10.1038/s41467-026-75582-9)
Supplement: Supplementary file 2 [file 41467_2026_75582_MOESM11_ESM.pdf]

nature portfolio

Peer Review File

Biophysical modeling of anatomically realistic prenatal cortical folding development

Corresponding Author: Professor Xianqiao Wang

This file contains all reviewer reports in order by version, followed by all author rebuttals in order by version.

Version 0:

Reviewer comments:

Reviewer #1

(Remarks to the Author)
This manuscript describes the use of computational modeling to explore the role of regional surface growth variations in generating cortical folding patterns during human fetal brain development.

Notable innovations include (i) the analysis of 120 fetal MR image sets to infer regional growth patterns and (ii) incorporation of those patterns into computational models of growth and elastic deformation of the brain. The key finding is that incorporation of actual spatially varying patterns of tangential cortical surface area expansion improves the accuracy of predictions of cortical folding.

Other strengths include the clear definition of the mechanical model, combining hyperelastic constitutive behavior and growth. The assumed growth behavior was also clearly described in terms of radial and tangential components in gray matter and isotropic volumetric grown in white matter, on an appropriately normalized time scale. Varying the type of growth (purely tangential vs other types), the cortical thickness and the temporal profile provided insights into their respective roles in determining the folding pattern.

Weaknesses are relatively minor. The procedures to estimate regional growth, a key step in the analysis, are only vaguely and superficially described. The effects of the assumed growth behavior in white matter (all sub-cortical tissue) are not investigated. The approximation of brain as purely elastic over the time scales of weeks ignores the well-documented viscoelastic properties of brain tissue, though it has the considerable merit of simplicity.

Specific comments and questions
1. Do the regionally varying surface growth patterns estimated from cross-sectional analysis in the fetal brain resemble previously reported growth patterns estimated from longitudinal image sets in premature infants (Garcia et al, PNAS, 2018)?
2. The volumetric growth in white matter seems likely to be an important parameter, but the current paper does not explore its effects on folding. How would increased or decreased white matter growth, or regional changes in WM growth affect folding patterns?
3. A related point is that it appears that everything that is not the outer cortical layer is described as “white matter” although much of it does not and will not consist of myelinated axonal fibers. Clarification of the terminology is warranted.
4. The similarity between predicted and observed folding patterns is evidence in support of the proposed model and mechanism. However, in principle, different mechanisms can produce similar shapes (kinematics). What do the stress fields look like in these models (i.e., in cortical gray matter, in white matter, in gyri, in sulci, ..)? Do the magnitudes and distributions of stress resemble those reported in the literature (Balouchzadeh et al., 2025, e.g.)?
5. The partitioning of the different regions, which then are analyzed to determine regional growth rates, is only superficially described in lines 554-557, with reference to a prior paper. Since this is such a key step in the analysis it would be good to have a more complete explanation in the methods or supplementary information in the current paper.
6. Brain tissue displays viscoelastic behavior and possibly stress-dependent growth, rather than the purely (hyper)elastic behavior assumed in the current model. While there is value in using the simplest possible model that recapitulates the observed folding pattern, how might incorporating viscoelasticity or stress-dependent growth affect predictions?

References

Balouchzadeh R, Kroenke CD, Garcia KE, Bayly PV. Measurement of Residual Stress in the Brain. J Biomech Eng. 2025 Sep 1;147(9):091001. doi: 10.1115/1.4068879.

Reviewer: Philip Bayly

(Remarks on code availability)

Reviewer #2

(Remarks to the Author)
The present study provides a rigorous, MRI data-driven approach to modeling the emergence of anatomical folds in the human brain. The authors present a finite element model of brain folding that incorporates regionally defined cortical growth trajectories on a realistic, complex initial brain geometry. Analyses comparing regional to uniform growth demonstrate that the data-driven regional growth model outperforms uniform growth models in reproducing features of normal brain folding, as well as examples of abnormal folding. Moreover, the authors use this model to illustrate how growth differences (and other factors such as initial cortical thickness) may translate to clinically observed folding abnormalities.

Altogether, this manuscript provides an important technical and conceptual contribution for the field that could lay the foundation for future studies expanding on this model. However, claims that the current model “accurately reproduces” human brain morphology are not consistent with the data shown. While this study has the potential for meaningful contribution to the field, several major and minor concerns should be addressed before this manuscript is suitable for publication.

Major Concerns:

1. Most crucially, simulated fold morphologies are not convincingly similar to real human brains. Statements to the effect that the models “accurately reproduce” realistic brain folding should be tempered throughout the manuscript. However, later comparisons of uniform vs regional growth (Fig. 5) provide convincing evidence that the regional growth models are superior to uniform growth models. Thus, restructuring the manuscript to begin with the different model options, then compare human data to both regional AND uniform growth models, would provide a more balanced and compelling presentation of results. In all comparisons, it would be interesting and informative to highlight remaining differences between the regional growth model and real brains, which are currently underreported. Where possible, statistics should be used to support statements of similarity and difference. (For example, local gyrification and sulcal depth comparisons between human data and models.)

2. The cortical growth model selected for primary analysis requires further justification. Based on the analysis shown, it is unclear that orthogonal growth with regional profile (OGR) is superior to tangential growth with regional profile (TGR). While the fold morphology for these two cases appears similar, one would expect differences in the measured regional area and cortical thickness trajectories, which can be quantified. In light of the inverted U-shape trajectory, I also wonder whether mechanics (radial strain resulting from compression) could influence or account for part of the observed thickness changes. (See, for example, Wang et al. BMMB 2021, Garcia et al. Dev Neurosci 2025). In determining the best model, it would be useful to compare measured model thickness and area trajectories (outputs) to the measured thickness and area trajectories from real brains, rather than only comparing the applied growth components. On minor but related note, it is not obvious that tangential growth is linear rather than exponential or some other form. This choice should also be justified.

3. While authors address several limitations in the discussion section, they should also recognize limitations inherent in the relatively coarse regions selected for this analysis. What if the true regional differences lie outside these predefined boundaries? The selected regions, if too coarse, may dilute the magnitude of spatial differences and obscure the true pattern. How well do the current patterns correspond to reported biological gradients? ROI-derived growth values also depend heavily on segmentation/labeling algorithms, which may or may not accurately capture evolving boundaries over time. These factors can drastically influence the estimated growth rates.

4. The trajectory comparisons in Fig. 7 are difficult to interpret. Was subcortical growth also held linear, or just the radial component of cortical growth? For the global comparisons (Fig. 7a–e), this seems crucial for understanding what drives the differences between models. (If all growth rates were assigned to any one model, there should be no difference at t=1.) In the figure caption and text, authors should explicitly describe if/how the ratio of cortical to subcortical growth rate is being altered over time and what they perceive to be the main driver of differences between models.

5. The current manuscript is quite long, owing in part to extensive technical analyses that may be of limited interest to the broader Nature Communications readership. For the sake of space, certain comparisons could be omitted or moved to supplement. For example, since local gyrification index, local sulcal depth, and other curvature metrics generally show the same trends, authors may choose one and optionally provide the rest in supplement. Similarly, less impactful / biologically relevant comparisons - especially of factors that have been reported in previous studies - could be shortened, omitted or moved to supplement.

Minor Concerns / Edits

1. For easier interpretation, consider using the same plot style (bar vs radial, etc.) for the same metric across figures and/or similar metrics within a figure.

2. In Fig. 2 and Fig. S3, caption for panel d: I think t = 1.0 should be t = 0.1.

3. Where Fig. S3 is first referenced, it would be better to say: "Growth patterns are not entirely symmetrical between the left hemisphere (Fig. 2) and right hemisphere (Fig. S3)." This clarifies what Fig. S3 actually is.

4. In panel e of Fig. 4, I cannot see "real brain" results for G32, 35, 38, but the legend indicates they should be there.

5. Why halve tangential growth ratio for regional pachygyria when "full pachygyria" was based on doubling cortical thickness? This seems inconsistent and needs explanation.

6. In Fig. S1, labels/arrows on the left schematic are confusing. It looks like proliferation only occurs in frontal cortex (etc.) which I assume is not what you mean. If these areas are linked to specific genes, it may be better to list genes explicitly on the relevant regions. Meaning of arrows is not apparent.

(Remarks on code availability)
I reviewed the Github site and it appears to have the necessary files for reproduction of their results. However, I did not review the codes in detail.

Version 1:

Reviewer comments:

Reviewer #1

(Remarks to the Author)
The revisions to the manuscript have addressed my concerns

(Remarks on code availability)

Reviewer #2

(Remarks to the Author)
The authors have made substantial improvements and performed valuable supplementary analysis in response to prior concerns. I feel the manuscript is now suitable for publication.

(Remarks on code availability)

Open Access This Peer Review File is licensed under a Creative Commons Attribution 4.0 International License, which permits use, sharing, adaptation, distribution and reproduction in any medium or format, as long as you give appropriate credit to the original author(s) and the source, provide a link to the Creative Commons license, and indicate if changes were made.
In cases where reviewers are anonymous, credit should be given to 'Anonymous Referee' and the source.
The images or other third party material in this Peer Review File are included in the article's Creative Commons license, unless indicated otherwise in a credit line to the material. If material is not included in the article's Creative Commons license and your intended use is not permitted by statutory regulation or exceeds the permitted use, you will need to obtain permission directly from the copyright holder.
To view a copy of this license, visit https://creativecommons.org/licenses/by/4.0/

RESPONSE TO REVIEWERS

We sincerely thank the Editor and the Reviewers for their thorough evaluation of our manuscript and for the constructive and insightful comments provided. We are grateful for the reviewers' positive assessment of the novelty and significance of our work, as well as their valuable suggestions to further strengthen the manuscript's quality. In response to these comments and suggestions, we have diligently revised the manuscript to improve clarity, expand methodological descriptions, provide additional quantitative analyses, and refine the organization and presentation of results. In this document, we provided detailed, point-by-point responses to each comment raised by the reviewers, with our responses highlighted in blue and all actions taken, including revisions and additions to the manuscript marked in red.

Reviewer #1:

This manuscript describes the use of computational modeling to explore the role of regional surface growth variations in generating cortical folding patterns during human fetal brain development.

Notable innovations include (i) the analysis of 120 fetal MR image sets to infer regional growth patterns and (ii) incorporation of those patterns into computational models of growth and elastic deformation of the brain. The key finding is that incorporation of actual spatially varying patterns of tangential cortical surface area expansion improves the accuracy of predictions of cortical folding.

Other strengths include the clear definition of the mechanical model, combining hyperelastic constitutive behavior and growth. The assumed growth behavior was also clearly described in terms of radial and tangential components in gray matter and isotropic volumetric grown in white matter, on an appropriately normalized time scale. Varying the type of growth (purely tangential vs other types), the cortical thickness and the temporal profile provided insights into their respective roles in determining the folding pattern.

Thank you for the concise summary and positive evaluation of our work. We greatly appreciate your recognition of the novelty of our data-driven regional growth modeling framework, and we are encouraged that your summary clearly captures the central contribution of our work, namely demonstrating the importance of spatially varying tangential growth in improving cortical folding predictions.

Weaknesses are relatively minor. The procedures to estimate regional growth, a key step in the analysis, are only vaguely and superficially described. The effects of the assumed growth behavior in white matter (all sub-cortical tissue) are not investigated. The approximation of brain as purely elastic over the time scales of weeks ignores the well-documented viscoelastic properties of brain tissue, though it has the considerable merit of simplicity.

Thank you for highlighting these important points. Although these are described as relatively minor weaknesses, we view them as valuable suggestions that can help improve the rigor and clarity of the manuscript. In response to these concerns, we have expanded the methodological description of growth regionalization and provided a more detailed discussion of the implications of white matter growth and viscoelastic effects in the point-by-point responses below.

Specific comments and questions

1. Do the regionally varying surface growth patterns estimated from cross-sectional analysis in the fetal brain resemble previously reported growth patterns estimated from longitudinal image sets in premature infants (Garcia et al, PNAS, 2018)?

Response: We thank the reviewer for this insightful question. Overall, our regionally varying tangential growth patterns show strong qualitative agreement with the longitudinal findings reported by Garcia et al. (PNAS, 2018), despite differences in subject cohorts and analysis methodology.

Garcia et al. used longitudinal MRI with accurate surface registration (aMSM) to quantify vertex-wise cortical expansion in preterm infants and reported pronounced spatiotemporal growth

heterogeneity[1]. In particular, between 30-38 gestational weeks, they observed relatively higher cortical expansion in lateral parietal, occipital, and temporal regions. In our study, although growth laws were derived from cross-sectional fetal data and summarized over 18 atlas-based regions per hemisphere, we similarly observed relatively higher tangential growth ratios in temporal (Region 5: Temporal Pole, Region 8: Superior Temporal, and Region 17: Middle and Inferior Temporal), parietal (Region 9: Supra Marginal), and occipital regions (Regions 14: Medial Occipital and Region 18: Lateral Occipital), especially from mid-gestation to term (see Fig. 2e, rightmost panel). Moreover, Garcia et al. reported comparatively lower expansion near medial and central regions in their median brain. This is consistent with our findings that regions such as the dorsal precentral and cingulate (Region 1) exhibit relatively lower tangential growth ratios.

Some differences are also present. For example, our analysis indicates relatively elevated growth in certain prefrontal and insula regions (e.g., Region 16: Dorsal Prefrontal and Region 2: Insula), whereas this trend is less pronounced in Garcia et al. These discrepancies are likely attributable to methodological differences: our growth trajectories are derived from region-level averages based on a developmental atlas, whereas Garcia et al. quantified expansion at the vertex level with longitudinal alignment. Regional averaging may attenuate or redistribute localized gradients. In addition, differences in subject cohorts (fetal cross-sectional vs. preterm longitudinal) and registration strategies may contribute to further regional variation.

In addition, Garcia et al. reported that weekly cortical expansion rates are approximately constant in many regions over the studied gestational window. This observation aligns with the near-linear tangential growth trajectories identified in our symbolic regression models (Fig. 2c). At the same time, they also reported the dynamic spatial shifts in growth patterns over development, suggesting nonlinear redistribution of expansion patterns across regions. Such migration of growth dominance is not fully captured by our current region-based framework. This represents an opportunity for future refinement using finer parcellations or vertex-level modeling.

2. The volumetric growth in white matter seems likely to be an important parameter, but the current paper does not explore its effects on folding. How would increased or decreased white matter

growth, or regional changes in WM growth affect folding patterns?

Response and Action: We appreciate the reviewer's insightful comment. White matter (WM) growth is indeed an important parameter in modeling brain folding development. In the present study, WM growth was characterized by measuring the volumetric variation of brain subjects across gestational ages in our dataset. Under the assumption of isotropic growth, we derived the growth function $g_w(t) = 0.41t + 1$ (Fig. S4), indicating that WM volume expands by approximately 2.5-fold between 22 and 40 gestational weeks (GWs).

To examine the effect of WM growth magnitude on cortical folding, we performed additional simulations by modifying the WM growth profile while keeping the heterogeneous gray matter (GM) growth model unchanged. Specifically, we considered two additional scenarios: (1) decreasing WM growth (Dec, $g_w(t) = 1$), in which WM active growth is suppressed, and (2) increasing WM growth by doubling the growth rate (Inc, $g_w(t) = 0.82t + 1$). In all cases, GM growth remained identical to that used in the manuscript, while WM growth remained isotropic.

As shown in Fig. S11a, increasing WM growth leads to a noticeable enlargement of the overall brain volume. However, the simulated primary folding patterns remains largely preserved, particularly when comparing the Dec and normal growth (Nor) cases. Although some local variations are observed, for example in Region 11 of the left hemisphere (caudal middle frontal), the Dec case exhibits fewer sulci compared with the Nor case; however, the majority of the primary sulci remain preserved. In the Inc case, the primary gyri become thicker while the sulci become relatively shallow, often accompanied by small superficial folds. Similar shallowing trends can also be observed when comparing the Nor case with the Dec case. These observations are confirmed by quantitative analysis of regional sulcal depth, defined as $\Delta\text{sulc} = (\text{sulc}_{\text{Dec/Inc}} - \text{sulc}_{\text{Nor}})/\text{sulc}_{\text{Nor}}$. As seen in Fig. S11b, the regional mean sulcal depth increases in all regions in the Dec case, whereas it decreases in the Inc case, consistent with the morphological patterns observed in Fig. S11a.

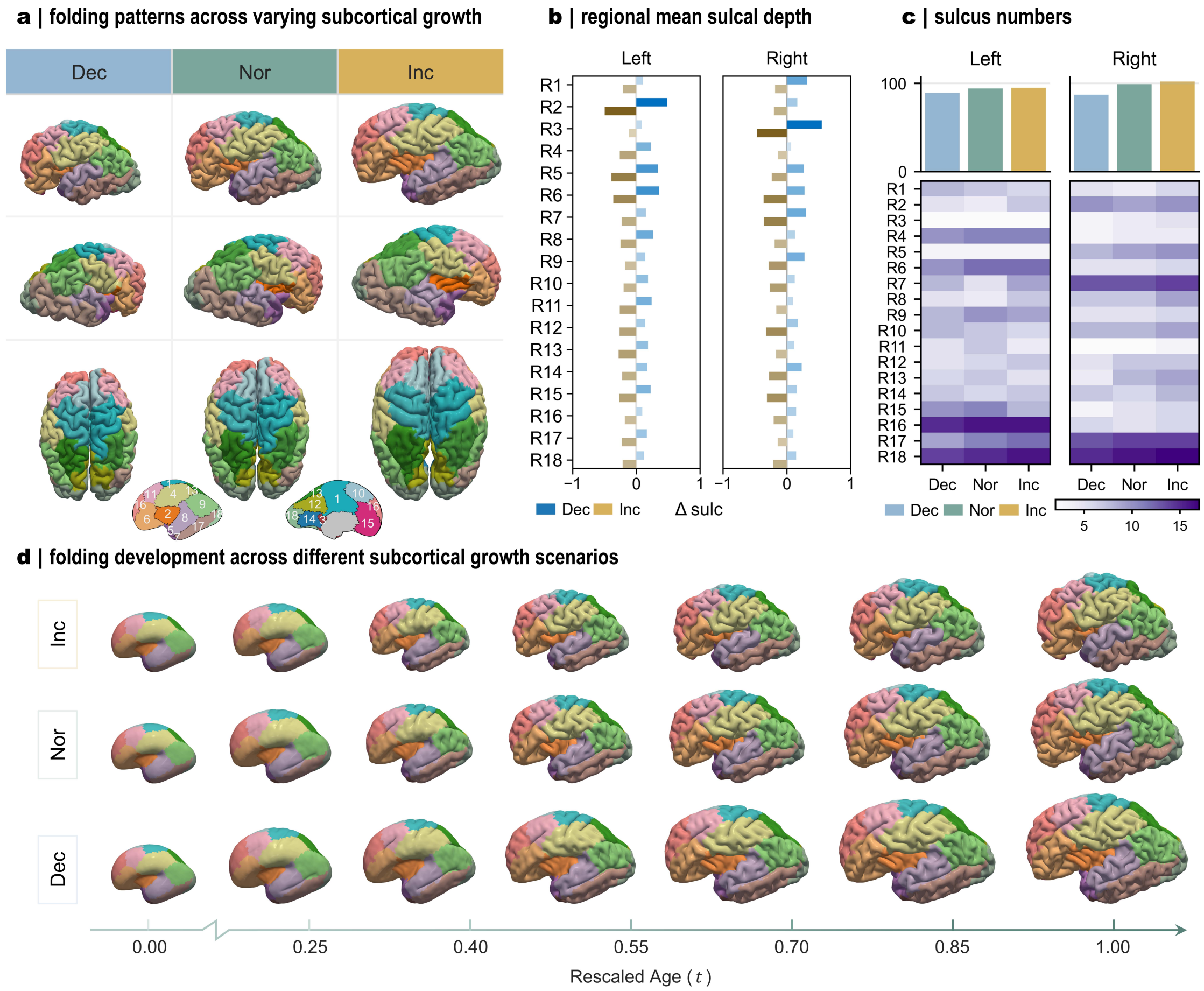

Fig. S11 | Effect of subcortical growth magnitude on cortical folding. **a**: Simulated cortical folding patterns across various subcortical growth conditions: normal growth (Nor, $g_w = 0.41t + 1$), decreased growth (Dec, $g_w = 1$), and increase growth (Inc, $g_w = 0.82t + 1$). Sagittal views of left and right hemispheres and axial views of whole brain are shown. Regional parcellation is provided at the bottom for reference. **b**: Regional difference in mean sulcal depth for the increased and decreased subcortical growth cases relative to the normal condition ($\Delta\mathrm{sulc} = (\mathrm{sulc}_{\mathrm{Dec/Inc}} - \mathrm{sulc}_{\mathrm{Nor}})/\mathrm{sulc}_{\mathrm{Nor}}$). **c**: Global (top) and regional (bottom) number of primary sulci across the three subcortical growth conditions. **d**: Representative cortical folding patterns under the three subcortical growth scenarios. In all simulations shown here, subcortical growth was treated as isotropic. Modifying subcortical growth magnitude does not substantially alter the spatial locations or wavelengths of primary folds, but it significantly affects sulcal depth and folding maturation dynamics.

From a mechanical perspective, when WM growth is suppressed (Dec case), WM deformation is primarily driven by GM expansion. Consequently, the WM imposes stronger deformation constraints on the cortex, promoting deeper sulcal folding. This effect is particularly evident in the downward bending deformation observed in the inferior frontal region (Region 6). In contrast, when WM growth is increased (Inc case), the WM expands isotopically with a larger magnitude,

which pushes the cortex outward and leads to thicker gyri and shallower sulci.

Despite these differences in sulcal depth, the number of primary folds remains nearly identical across the three cases. As seen in Fig. S11c, both global and regional sulcal counts are comparable, with the Dec case showing slightly fewer folds due to a longer folding maturation range (Fig. S11d). Notably, primary sulci were identified using the same procedure described in the manuscript, based on a mean-curvature–based detection combined with an area-based filtering criterion (minimum area of 10 mm^2 and at least 60 vertices per patch). This filtering procedure removes small and underdeveloped shallow folds, particularly those observed in the Inc case. Furthermore, analysis of the folding development history (Fig. S11d) shows that noticeable folds emerge relatively later when WM growth is increased, resulting in a shorter maturation window for sulcal deepening and merging. Overall, these results suggest that modifying WM growth magnitude does not substantially alter the spatial locations or wavelengths of primary folds, but it significantly affects sulcal depth and folding maturation dynamics.

In addition to isotropic WM growth, we further investigated the effects of regional heterogeneity in WM growth. As shown in Fig. S12a, we partitioned the WM into 12 regions (six per hemisphere) by roughly combining the 18 cortical regions used in our study into larger regional patches. The resulting regions approximately correspond to the temporal lobe, occipital lobe, parietal lobe, central frontal lobe, frontal lobe, and insula (Regions 1-6). We note that this regionalization was introduced solely to explore potential effects of regional WM growth and does not represent a precise anatomical parcellation. Using this regionalization, we implemented three heterogeneous WM growth scenarios by modifying regional growth ratios relative to the baseline WM growth ($g = g_w$), as illustrated in Fig. S12b. To further explore possible effects of regional WM variation, we also included regional shrinkage by assigning negative growth ratios. Although such shrinkage does not represent typical developmental processes, it may serve as a simplified proxy for abnormal growth conditions associated with brain malformations. In all cases, the GM growth profile remained identical to that used in the manuscript.

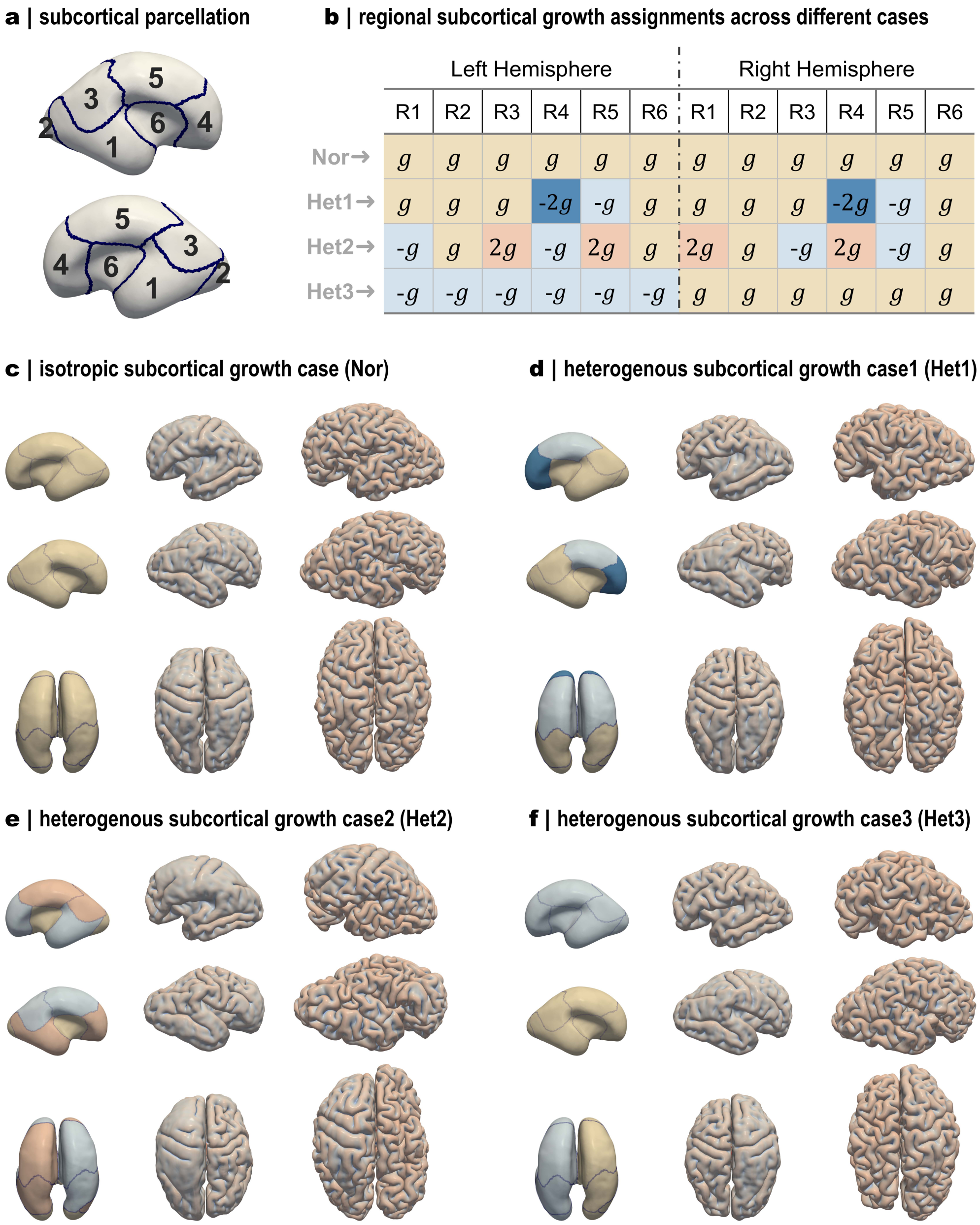

Fig. S12 | Effect of heterogeneous subcortical growth on cortical folding. **a**: Illustration of subcortical parcellation in the left and right hemispheres. Black lines denote boundaries between adjacent regions. **b**: Regional growth assignments for the isotropic growth condition (Nor) and three different heterogenous growth scenarios (Het1, Het2, and Het3). g denotes the baseline subcortical growth ($g = g_w$). **c-f**: Simulated cortical folding patterns under isotropic and heterogeneous subcortical growth scenarios. Sagittal views of left and right hemispheres and axial views of the whole brain are shown. For each case, the left panel shows the regional growth assignment (colored according to the growth ratios in panel b), the middle panel shows the folding patterns at mid-simulation ($t = 0.5$) and the right panel shows the final folding patterns ($t = 1.0$). Regional variations in subcortical growth can locally modulate cortical folding morphology and produce spatially heterogeneous folding patterns.

In heterogeneous case 1, degrowth was applied to the prefrontal regions (R4 and R5) in both hemispheres. Compared with the normal brain case (Fig. S12c), this regional degrowth produces noticeable local volume reduction, leading to wider and deeper sulci and thinner gyri in the affected regions, while folding patterns in the unaffected regions remain largely unchanged (Fig. S12d). In heterogeneous case 2, asymmetric regional growth patterns were assigned between hemispheres. Specifically, we amplified the growth in parietal areas (R3) of left brains by doubling the growth ratio, while applying degrowth to corresponding region in the right hemisphere. Similar asymmetric assignments were applied to the temporal (R1) and frontal regions (R4 and R5). As seen in Fig. S12e, these heterogeneous growth patterns produce highly non-uniform cortical folding, with degrowing regions generating deeper and wider sulci and regions with enhanced growth producing fatty gyri and shallow sulci. In heterogenous case 3, we applied degrowth to the entire left hemisphere while the right hemisphere maintained the baseline growth profile. As shown in Fig. S12f, this produces a noticeable volume reduction and folding patterns similar to those observed in other degrowth scenarios within the left hemisphere.

Taken together, these results indicate that regional variations in WM growth can locally modulate cortical folding morphology and produce spatially heterogeneous folding patterns. This suggests that heterogeneous WM development may also contribute to shaping cortical folding patterns, similar to the role of heterogeneous GM growth. In reality, human WM is likely more heterogeneous than GM due to its complex axonal architecture and functional organization. Incorporating more accurate quantitative descriptions of WM development may therefore further improve the predictive capability of our modeling framework. Future work will focus on integrating such biological constraints to more systematically investigate the individual and coupled effects of WM and GM growth heterogeneity in cortical folding development. In addition, the regional analyses presented here provide a useful framework for studying regional brain malformations. The diverse folding patterns observed in Fig. S12 further demonstrate the robustness and flexibility of our modeling framework.

Based on this additional analysis of WM growth effects, we have added two new figures in the

Supplementary Materials (Figs. S11-S12) and incorporated corresponding discussions in the revised manuscript.

Additionally, we explored the effects of varying subcortical growth, including changes in growth magnitude and regional heterogeneity (Figs. S11-S12). These analyses show that variations in subcortical growth magnitude primarily influence brain volume and depth of sulci, while largely preserving the number and overall pattern of primary cortical folds. (see "Effect of varying subcortical growth in modeling cortical folding" in Supplementary Materials for details).

3. A related point is that it appears that everything that is not the outer cortical layer is described as "white matter" although much of it does not and will not consist of myelinated axonal fibers. Clarification of the terminology is warranted.

Response and Action: Thank you for raising this important point regarding terminology. We agree that in the developing fetal brain, much of the tissue beneath the cortical plate does not yet consist of mature myelinated axonal fibers and therefore does not strictly correspond to "white matter". To avoid potential confusion, we have revised the terminology in the manuscript and now refer to this region as the subcortex (subcortical tissue), noting that it represents the collection of developing tissue layers beneath the cortex. For consistency, "gray matter" has also been renamed as cortex (cortical tissue) throughout the manuscript. Corresponding revisions have been made across the text to improve clarity.

4. The similarity between predicted and observed folding patterns is evidence in support of the proposed model and mechanism. However, in principle, different mechanisms can produce similar shapes (kinematics). What do the stress fields look like in these models (i.e., in cortical gray matter, in white matter, in gyri, in sulci, ..)? Do the magnitudes and distributions of stress resemble those reported in the literature (Balouchzadeh et al., 2025, e.g.)?

Response and Action: We thank the reviewer for this thoughtful comment. Stress distributions provides additional insight into the mechanisms underlying cortical folding and can serve as an important criterion for evaluating the physical plausibility of the proposed modeling framework.

In response, we analyzed the stress fields in our simulations and used the maximum (absolute) principal stress as a representative metric for both qualitative and quantitative evaluation, following the approach used in the study suggested by the reviewer.

As shown in Fig. S7a, the cortical layer consistently experiences compressive stresses, while the underlying subcortex, particularly near the gyri and sulcal fundi, exhibits localized tensile stresses. These spatial patterns are consistent with experimental measurements[2-4] and previous computational studies of cortical folding[5,6] and also align with the differential-growth–driven bilayer instability framework. According to the theory, mechanical instability arises from constrained deformation between two coupled layers induced by differential tangential expansion[7]. In our simulations, compressive stresses primarily develop in the cortex as a result of deformation mismatch between the rapidly growing cortex and the more slowly growing subcortex. Once these compressive stresses exceed a critical threshold, they are relieved through mechanical buckling, leading to the emergence of gyri and sulci[8,9]. Analogous to classical bending mechanics, the subcortex beneath gyri experiences radial tension, whereas regions beneath sulci experience tangential tension[10]. Additionally, consistent with previous biomechanical models of cortical folding, stress magnitudes in our simulation decreased with depth from the cortex-subcortex interface. The deeper subcortical regions experience near-neutral or weak compressive stresses, indicating that these tissues primarily serve as a mechanical substrate supporting cortical folding. Such stress gradients help protect deep subcortical nuclei from large deformations during cortical expansion, while allowing folding to remain localized near the cortical surface[8].

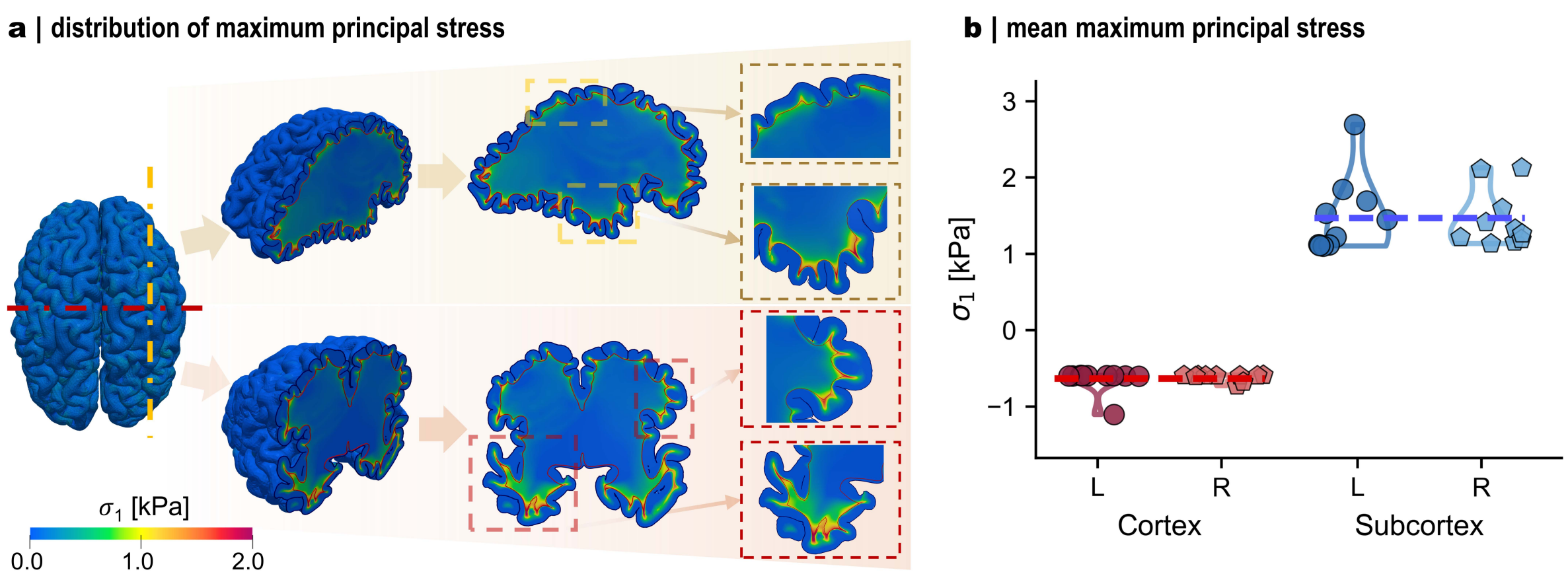

Fig. S7 | Stress distribution in simulated brains. **a**: Distribution of maximum (absolute) principal stress in the cortex and subcortex of simulated brains. Sagittal and coronal sections are shown. Insets on the right display magnified views of the local stress distribution. The cortical boundary and the cortex–subcortex interface are highlighted by black and red lines, respectively. **b**: Averaged values of maximum (absolute) principal stress in the cortex and subcortex across the ten subjects analyzed in this study. Red and blue dash lines indicate the mean values across subjects and hemispheres. Violin plots illustrate the distribution of the measurements. Cortical layer consistently experiences compressive stresses, while the underlying subcortex, particularly close to the gyrus, exhibits localized tensile stresses.

In addition to the qualitative agreement in stress distributions, the magnitudes of stresses predicted by our simulations are also consistent with values reported in the literature. To quantify this, we computed the maximum (absolute) principal stress across the ten subjects analyzed in this study and calculated the mean values for both hemispheres. As seen in Fig. S7b, the cortex exhibits an average maximum stress of approximately 0.7 kPa, while the subcortex shows an average stress of approximately 1.5 kPa. The compressive stress values fall within the range reported for the minipig brain (a gyrencephalic large mammal) in Balouchzadeh, et al. [3], while the tensile stress is of the same order of magnitude but slightly lower than the reported values (~3 kPa). The relatively lower tensile stress observed in our simulations may partly result from the averaging procedure used in our analysis. Specifically, stresses were averaged across the entire subcortical region and across all elements, including deeper regions that experience relatively weak compressive stresses, which reduces the overall mean value. Additional factors, such as species differences and modeling simplifications, also contribute to these quantitative differences.

Overall, both the spatial distribution and the magnitude of stresses predicted by our simulations

are consistent with those reported in the literature. This supports the proposed differential growth mechanism and further demonstrates the physical plausibility of our modeling framework. To clarify these mechanical features, we have added the analysis of stress fields in the revised Supplementary Materials (Fig. S7) and included corresponding statements in revised manuscript to describe the stress states during cortical folding.

Beyond morphometric analyses, we further examined the mechanical state by evaluating stress distributions during the simulations (Fig. S7). Both the spatial distribution, characterized by compressive stresses in the cortex and tensile stresses in the subcortex, and the overall stress magnitudes agree with prior computational studies[19,41] and experimental measurements[42].

5. The partitioning of the different regions, which then are analyzed to determine regional growth rates, is only superficially described in lines 554-557, with reference to a prior paper. Since this is such a key step in the analysis it would be good to have a more complete explanation in the methods or supplementary information in the current paper.

Response and Action: We thank the reviewer for highlighting the need for a clearer description of the cortical regional partitioning used in our analysis. We agree that this step is important because the regional growth models are derived based on this parcellation. In this study, cortical regions were defined using a previously established data-driven developmental regionalization map derived from large-scale prenatal and neonatal neuroimaging datasets[11]. Specifically, the regionalization was obtained using non-negative matrix factorization (NMF) applied to vertex-wise cortical surface area data from nearly 1,000 MRI scans (primarily spanning 29-45 gestational weeks) from the developing Human Connectome Project (dHCP) and the Baby Connectome Project (BCP).

In this approach, a non-negative data matrix $V \in \mathbb{R}^{i \times j}$ was constructed, where i represents the number of cortical vertices and j represents the number of scans. Each column of V corresponds to the vertex-wise cortical surface area of one scan. NMF decomposes this matrix into two non-negative matrices $W \in \mathbb{R}^{i \times k}$ and $H \in \mathbb{R}^{k \times j}$ such that $V \approx WH$, where k denotes the number of

spatial components (regions), typically satisfying $k \ll i$ and $k \ll$ j. The columns of W represent spatial components that group vertices having similar developmental trajectories of surface area expansion, thereby defining coherent cortical subregions, while H represents the corresponding coefficient matrix describing the contribution of each component across scans.

To determine the appropriate number of regions, two complementary criteria were jointly used: the silhouette coefficient, which evaluates intra-region similarity and inter-region separation based on vertex-wise correlations of developmental patterns, and the reconstruction error, defined as $\|V - WH\|_F^2$. The optimal number of regions was determined by identifying solutions with a higher silhouette coefficient and lower reconstruction error. Finally, a hard parcellation map was obtained by assigning each vertex to the component with the largest weight in W, resulting in the final cortical regionalization.

In the present study, we adopted this previously derived regionalization map rather than recomputing the parcellation from our dataset. The primary reason is that NMF-based regionalization requires a large sample size with broad and continuous developmental coverage to reliably estimate vertex-wise developmental trajectories[12-14]. Our current dataset contains 120 subjects with ages ranging from 22-40 gestational weeks, with the majority of scans (109 subjects) concentrated between 29-40 gestational weeks. Such limited and uneven developmental coverage is insufficient to robustly derive a stable NMF-based regionalization from the current dataset alone. Moreover, since most of the data used in the present study (29-40 gestational weeks) originate from the larger dataset on which the regionalization was originally established, we considered the previously derived large-scale regionalization map as a robust and biologically meaningful framework for analyzing regional cortical growth patterns in our study.

Following the reviewer's suggestion, we have expanded the description of the regionalization procedure in the Supplementary Materials. We also clarify in the manuscript that the parcellation was derived from large-scale developmental datasets and subsequently applied to our cohort.

The parcellation was obtained using a data-driven framework based on non-negative matrix factorization (NMF), which groups cortical vertices according to shared developmental trajectories

of surface area expansion[4] (see Supplementary Materials for methodological details).

Finally, we acknowledge that deriving a study-specific regionalization map could further refine the analysis when sufficient data become available. This point has now been explicitly discussed as limitation in the Discussion section.

Third, both the estimation of growth trajectories and subsequent analyses are performed based on parcellated regions; such regional averaging over relatively coarse parcellations may obscure fine-scale spatial heterogeneity present in real brains. In this study, the cortical regionalization map was adopted from a previously established large-scale developmental dataset rather than derived from the current cohort. This is because NMF-based regionalization requires large datasets with broad and continuous developmental coverage to reliably estimate vertex-wise developmental trajectories. Future studies with larger and more uniformly distributed datasets may enable the derivation of more robust, cohort-specific regionalization maps.

6. Brain tissue displays viscoelastic behavior and possibly stress-dependent growth, rather than the purely (hyper)elastic behavior assumed in the current model. While there is value in using the simplest possible model that recapitulates the observed folding pattern, how might incorporating viscoelasticity or stress-dependent growth affect predictions?

Response and Action: Thank you for this insightful comment. To assess the potential impact of the viscoelasticity on folding predictions, we performed an additional set of simulations following the stress-dependent growth framework introduced by Balouchzadeh, et al. [15]. In these simulations, the subcortex was modeled as a viscoelastic material using a Prony-series formulation, whereas the cortex remained the hyperelastic and followed the heterogeneous growth model used in the primary simulations. In this setting, white matter continued to undergo isotropic growth, and shear modulus relaxation was defined as $\mu(t) = \mu_0\left[(1 - g_1) + g_1 e^{-t/\tau_1}\right]$. Here, μ_0 is the instantaneous shear modulus, g_1 represents the fraction of stress that relaxes, and the term $\mu_0(1 - g_1)$ corresponds to the long-term relaxation modulus, τ_1 defines the relaxation timescale and is expressed as a fraction of the step time T, such that $\tau_1 = \alpha T$.

We specially examined the influence of the two key parameters: g_1 and α_1. Specifically, we tested three representative values for each parameter ($g_1 = 0.1,0.5,0.9$; $\alpha = 0.2,0.5,0.8$), where larger g_1 corresponds to a greater fraction of stress relaxation and larger α leads to slower relaxation. As shown in Fig. S13a, the primary folding patterns remain largely consistent with those obtained using the baseline hyperelastic model. However, incorporating stress-dependent effects produces slightly smoother cortical patterns compared with the baseline case. For small parameter values (e.g., $g_1 = 0.1$, $\alpha = 0.2$), the simulated folding patterns are nearly identical to the baseline model. This is expected because only a small fraction of stress relaxes and relaxation occurs rapidly, resulting in minimal influence on the overall growth-driven instability. In contrast, larger relaxation parameters lead to noticeable changes in folding morphology. For example, when the relaxation fraction and timescale are increased, folds become denser with shallower sulci and smaller gyri. Quantitative analysis further supports this observation. Using the baseline model as reference, we computed the percentage deviation in sulcal depth across cortical regions (Fig. S13b). Most regions exhibit reduced sulcal depth relative to the baseline model, with only minor positive deviations observed in a few regions (e.g., R5). These results are consistent with the findings reported by Balouchzadeh, et al. [15], who also observed reduced sulcal depth when stress-dependent growth was incorporated.

Although brain tissue is viscoelastic in nature, cortical folding during prenatal development occurs over relatively long timescales, which can often be approximated as quasi-static. Under such conditions, hyperelastic formulations have been widely adopted in computational models of brain folding[16-19]. Nevertheless, viscoelasticity remains an important factor that may influence stress redistribution and growth dynamics[10]. Additionally, the Prony-series model used here represents a linear viscoelastic approximation, while brain tissue undergoes large deformations during development. Future studies incorporating more advanced nonlinear viscoelastic constitutive models and systematic parameter exploration may therefore provide further insight into the role of viscoelasticity in whole-brain folding simulations.

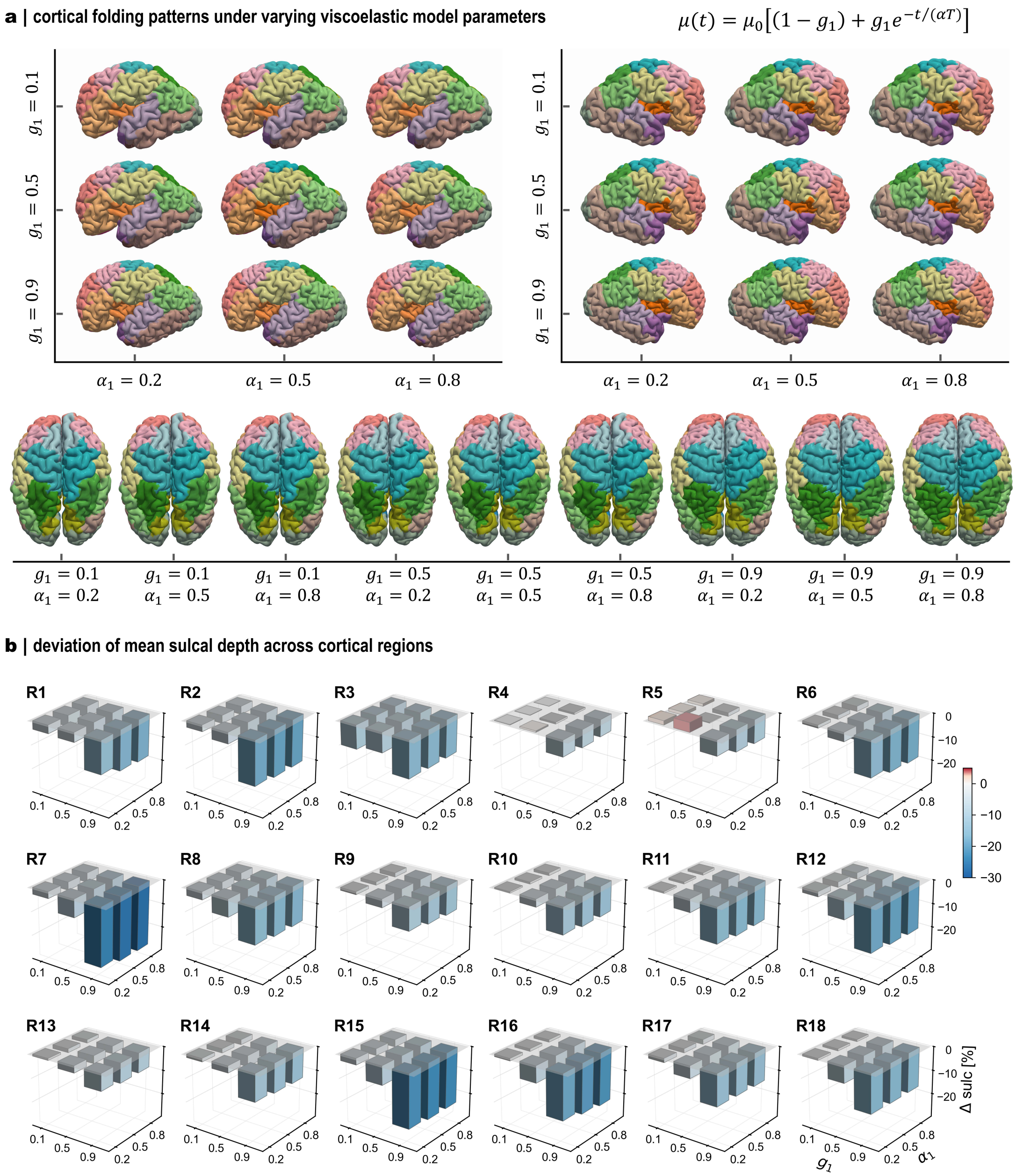

Fig. S13 | Cortical folding patterns incorporating stress-dependent growth. **a**: Cortical folding patterns under varying viscoelastic parameter settings. Sagittal views of the left and right hemispheres and axial views of the whole brain are shown. The viscoelastic model, represented by a single-term Prony series, is illustrated at the top right for reference. **b**: Percentage deviation of regional mean sulcal depth relative to the baseline model. The differential growth model without stress-dependent growth is used as the reference. The zero-difference level is indicated by a transparent gray plane. Incorporating viscoelasticity primarily smooths cortical folding patterns and slightly reduces sulcal depth, while the global organization of the primary folds remains largely unchanged.

Reviewer #2:

The present study provides a rigorous, MRI data-driven approach to modeling the emergence of anatomical folds in the human brain. The authors present a finite element model of brain folding that incorporates regionally defined cortical growth trajectories on a realistic, complex initial brain geometry. Analyses comparing regional to uniform growth demonstrate that the data-driven regional growth model outperforms uniform growth models in reproducing features of normal brain folding, as well as examples of abnormal folding. Moreover, the authors use this model to illustrate how growth differences (and other factors such as initial cortical thickness) may translate to clinically observed folding abnormalities.

Altogether, this manuscript provides an important technical and conceptual contribution for the field that could lay the foundation for future studies expanding on this model. However, claims that the current model "accurately reproduces" human brain morphology are not consistent with the data shown. While this study has the potential for meaningful contribution to the field, several major and minor concerns should be addressed before this manuscript is suitable for publication.

We thank the reviewer for the careful evaluation of our manuscript and for the accurate summary of our work. We appreciate the recognition of the technical contributions and potential translational relevance of our modeling framework. At the same time, we agree that the current model does not fully reproduce the detailed morphology of the human brain due to several simplifying assumptions. Accordingly, we have moderated the previously overstated wording and revised the manuscript following the reviewer's suggestions, as detailed below.

Major Concerns:

1. Most crucially, simulated fold morphologies are not convincingly similar to real human brains. Statements to the effect that the models "accurately reproduce" realistic brain folding should be tempered throughout the manuscript. However, later comparisons of uniform vs regional growth (Fig. 5) provide convincing evidence that the regional growth models are superior to uniform

growth models. Thus, restructuring the manuscript to begin with the different model options, then compare human data to both regional AND uniform growth models, would provide a more balanced and compelling presentation of results. In all comparisons, it would be interesting and informative to highlight remaining differences between the regional growth model and real brains, which are currently underreported. Where possible, statistics should be used to support statements of similarity and difference. (For example, local gyrification and sulcal depth comparisons between human data and models.)

Response and Action: Thank you for this insightful comment. We agree that the previous wording, such as "accurately reproduce," overstated the model performance. Accordingly, we have revised these statements throughout the manuscript and replaced them with more appropriate expressions (e.g., "capture" or "recapitulate") to better reflect that the model reproduces the major characteristics of cortical folding (primarily the primary folding patterns), while not fully replicating the detailed morphology of the human brain.

Following the reviewer's suggestion, we have also restructured the Results section to provide a more balanced presentation. Specifically, the comparison between different modeling strategies (uniform versus regional growth) is now presented earlier in the manuscript. This comparison demonstrates that the regional growth model consistently outperforms the uniform growth model in capturing key folding features. Based on these results, we then adopt the heterogeneous growth model for subsequent analyses, where a comprehensive comparison between simulated and real brains are presented (now shown in Figs. 4 and 5). These evaluations assess model performance from both qualitative and quantitative perspectives. We assessed the similarities and differences at multiple levels, including global metrics, regional averages, and vertex-wise distributions. In particular, we added statistical comparisons of regional sulcal depth between simulated and real brains, as suggested by the reviewer. The original Fig. 4 (now Fig. 5) has been updated to include deviation analyses at both vertex-wise and regional levels, together with statistical tests for regional sulcal depth (Fig. 5b). The corresponding descriptions in the manuscript have been revised to clearly highlight both agreements and remaining differences between the simulations and real

brains. In addition, we included analysis of the stress distribution during folding (Fig. S7) to further demonstrate the mechanical plausibility of the simulated folding patterns.

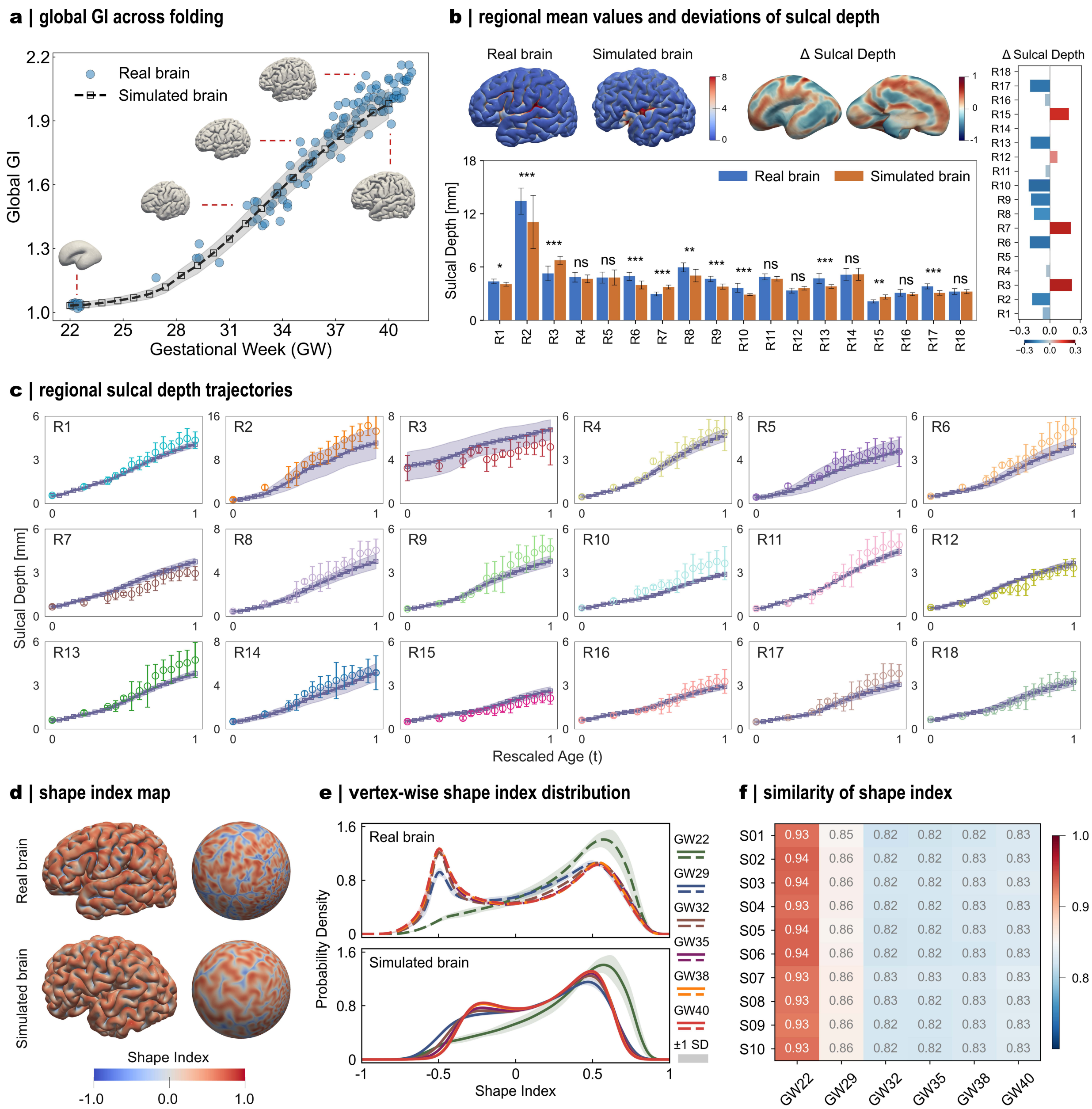

Fig. 5 | Modeled brains quantitatively recapitulate realistic folding patterns across scales. **a**: Developmental trajectory of global gyrification index (GI) in real and simulated brains. Insets show real brain folding patterns for typical data points. Simulation data are shown as mean ± s.d. (n=10). **b**: Distribution of vertical deviation in sulcal depth between simulated and real brains at 40 GWs (top left). Deviation is computed using the mean of ten simulated brains and the mean of real brains near 40 GWs, and is visualized on the 22-GW real brain surface. Regional sulcal depth across 18 cortical regions for simulated and real brains are summarized in the bottom left

panel. Data are shown as mean ± s.d., statistical significance was evaluated using the Wilcoxon signed-rank test (n = 20, 10 per case). ***$P < 0.001$; **$P < 0.01$; $P < 0.05$; ns, not significant. Right panels show regional deviations in sulcal depth relative to the real brains. **c**: Trajectories of regional mean sulcal depth across 18 regions. Simulation data are shown as mean ± s.d. (n=10). Dots and error bar indicate sulcal depth from 120 subjects. **d**: Distribution of shape index in real and simulated brains, displayed on folded and spherical surfaces. **e**: Shape index distribution at all vertices across six gestational ages with data shown as mean ± s.d. **f**: Average similarity of shape index between ten simulated brains and real brains, corresponding to panel e.

We did not include the uniform growth model in the simulated-versus-real brain comparison because earlier results (Fig. 3) already show that the uniform growth model substantially overpredicts cortical thickness and underestimates the number of primary sulci relative to real brains. These results provide clear evidence supporting the superior performance of the heterogeneous growth model. Including the uniform model again in Fig. 5 would substantially increase visual complexity and reduce the clarity of the figure. Instead, we provide an additional comparison of folding metrics between the uniform (IGU) and heterogenous (OGR) growths in the Supplementary Materials (Fig. S5).

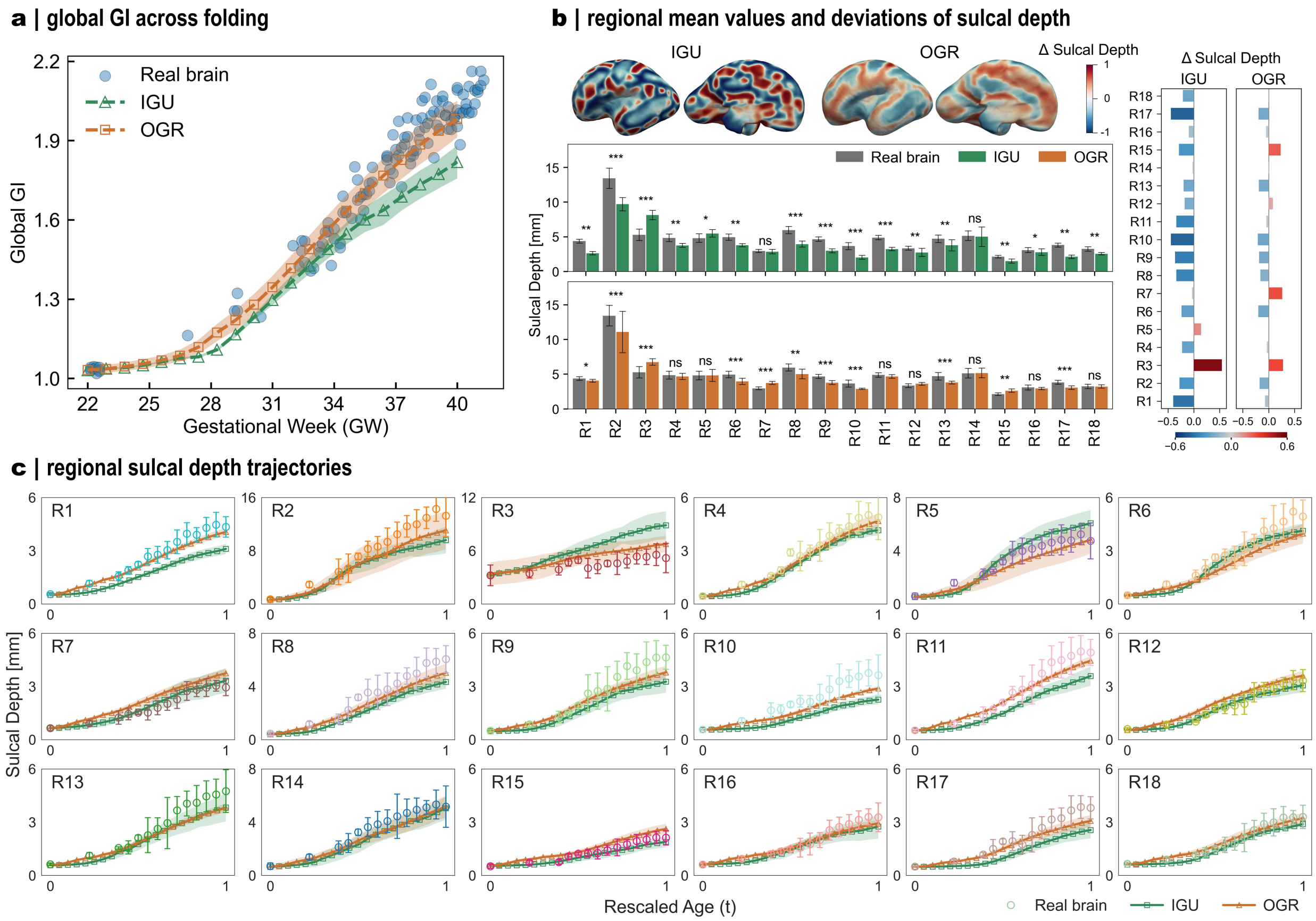

Fig. S5 | Comparison of folding metrics between uniform (IGU) and heterogeneous (OGR) growth models. **a**: Developmental trajectory of global gyrification index (GI) in real and simulated brains. Insets show real brain folding patterns for typical data points. Simulation data are shown as mean ± s.d. (n=10). **b**: Comparison of regional mean sulcal depth. Distribution of sulcal depth deviation (top left). Deviation is computed using the mean of ten simulated brains and the mean of real brains near 40 GWs, and is visualized on the 22-GW real brain surface. Regional sulcal depth across 18 cortical regions for simulated and real brains are summarized in the bottom left panel. Data are shown as mean ± s.d., statistical significance was evaluated using the Wilcoxon signed-rank test (n = 20, 10 per case). ****P* < 0.001; ***P* < 0.01; *P* < 0.05; ns, not significant. Right panels show regional deviations in sulcal depth relative to the real brains. **c**: Trajectories of regional mean sulcal depth across 18 regions. Simulation data are shown as mean ± s.d. (n=10). Dots and error bar indicate sulcal depth from 120 subjects.

We have added the following statements to the revised manuscript to reflect these changes:

Consistent trends are observed in global GI and sulcal depth trajectories, which further demonstrate improved agreement with real brains for heterogeneous growth (OGR) compared to uniform growth (IGU) (Fig. S5).

We appreciate the reviewer's thoughtful suggestion, which helped us improve the organization and clarity of the manuscript.

2. The cortical growth model selected for primary analysis requires further justification. Based on the analysis shown, it is unclear that orthogonal growth with regional profile (OGR) is superior to tangential growth with regional profile (TGR). While the fold morphology for these two cases appears similar, one would expect differences in the measured regional area and cortical thickness trajectories, which can be quantified. In light of the inverted U-shape trajectory, I also wonder whether mechanics (radial strain resulting from compression) could influence or account for part of the observed thickness changes. (See, for example, Wang et al. BMMB 2021, Garcia et al. Dev Neurosci 2025). In determining the best model, it would be useful to compare measured model thickness and area trajectories (outputs) to the measured thickness and area trajectories from real brains, rather than only comparing the applied growth components. On minor but related note, it is not obvious that tangential growth is linear rather than exponential or some other form. This choice should also be justified.

Response and Action: Thank you for this instructive comment. OGR and TGR share the same tangential growth component but differ in their treatment of cortical thickness: TGR does not explicitly prescribe radial (thickness) growth, whereas OGR incorporates a regionally varying thickness profile derived from imaging data. Both models outperform the uniform growth cases (IGU and TGU) in reproducing folding patterns (Fig. 3). However, we agree that folding morphology alone is insufficient to distinguish between OGR and TGR, as their qualitative patterns and key metrics (e.g., regional local GI, sulcus depth, and primary sulcal counts) are similar, and that additional quantitative evaluation is necessary to support model selection.

Following the reviewer's suggestion, we have added a new quantitative comparison of developmental trajectories of cortical surface area and cortical thickness between simulations and real brain data (Fig. S6). The results show that both OGR and TGR reproduce regional surface area trajectories well (Fig. S6a), which is expected given their identical tangential growth formulation. In contrast, cortical thickness trajectories from both models deviate from the

measured data, exhibiting an inverted U-shaped pattern with elevated final thickness values across most regions (Fig. S6b).

These discrepancies can be attributed to several factors. First, radial growth is substantially smaller than tangential growth (typically ~0.1 vs ~1.5), and in some regions follows an inverted-U profile with near-zero net growth. This makes folding primarily driven by tangential expansion, which in turn modulates cortical thickness patterns through geometric and mechanical coupling (e.g., bending-induced deformation and curvature-dependent strain redistribution). As a result, apparent thickness changes can arise even in the absence of explicitly prescribed radial growth, as detailed in the following mechanics discussion. This coupling provides a mechanistic explanation for why both OGR and TGR exhibit similar regional cortical thickness trajectories. Second, the absence of skull confinement in the model allows continued outward expansion of gyri, leading to increased cortical thickness relative to *in vivo* conditions. In addition, the use of a purely hyperelastic material model neglects viscoelastic relaxation, which in real brain tissue would mitigate thickness accumulation. Together, these factors explain why the simulated cortical thickness at the plateau stage is higher than that observed in real brains. Finally, another potential factor is that, in our simulations, cortical thickness and surface area expansion evolve simultaneously, whereas imaging studies suggest that cortical thickening precedes areal expansion during development[20]. This divergence in developmental progression may lead to distinct morphological outcomes.

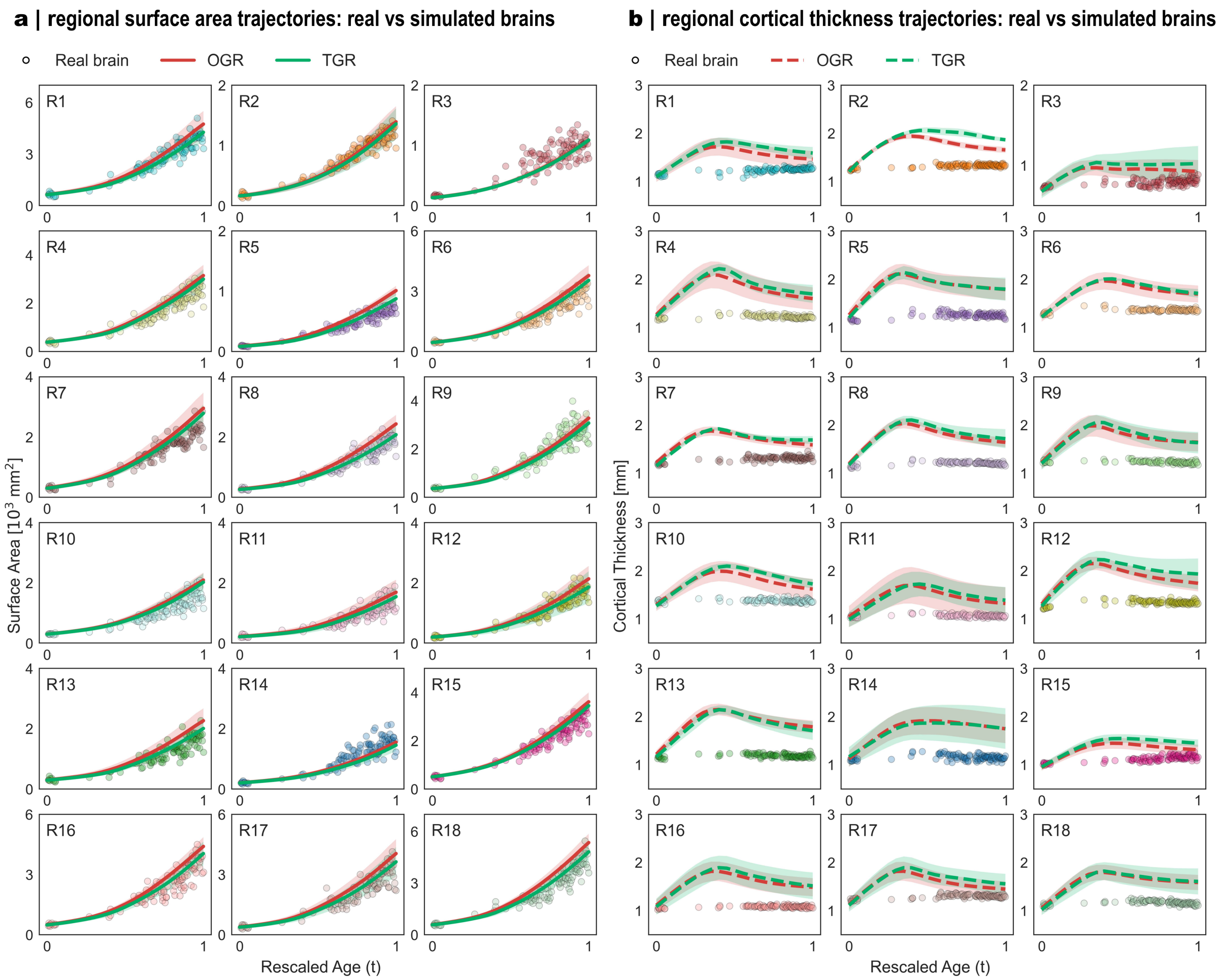

Fig. S6 | Regional trajectories of surface area and cortical thickness in real and simulated brains. **a-b**: Developmental trajectories of surface area (a) and cortical thickness (b) across 18 cortical regions. Simulation results are shown for the orthotropic regional growth (OGR) and tangential regional growth (TGR) conditions (see Fig. 3 in the main manuscript). Simulation curves are shown as mean ± s.d. (n=10). Dots denote measurement from real brains obtained from 120 subjects.

Despite these limitations, both models capture the overall inverted U-shaped thickness trend observed in real data (Fig. 2b). Notably, OGR generally shows improved, or at least comparable, agreement with measured cortical thickness compared to TGR across most regions (e.g., regions 1, 2, 3, 12, 15, and 17). These results suggest that incorporating regionally prescribed thickness contributes to more realistic developmental trajectories. Therefore, the OGR growth treatment is selected for modeling cortical folding patterns. We have added Fig. S6 in the Supplementary Materials and included corresponding discussion in the revised manuscript to clarify this point.

Notably, TGR and OGR differ only in radial growth, with OGR incorporating region-specific thickness regulation. Their similar folding patterns and close agreement in regional metrics measurements suggest that variations in radial growth have limited influence on overall folding geometry under the tested conditions. Despite the inclusion of prescribed thickness growth, simulated cortical thickness deviates from empirical measurements (Fig. 3c; Fig. S6), suggesting that thickness patterns are influenced by tangential-growth-driven folding through geometric and mechanical coupling[21] (see Supplementary Materials). However, comparison between TGR and OGR shows that incorporating region-specific radial growth leads to improved agreement of cortical thickness trajectories with real brain data (Fig. S6). This relative improvement indicates that thickness regulation enhances the fidelity of simulated folding patterns. Based on these results, the OGR configuration was selected for subsequent simulations.

Regarding the reviewer's comment on mechanical effects, we agree that mechanics indeed plays a fundamental role in shaping cortical thickness. In our simulations, early tangential expansion of the cortical layer against the relatively constrained subcortical substrate generates in-plane compressive stress[7]. Due to the near-incompressibility of brain tissue, this tangential compression is partially accommodated by out-of-plane deformation, leading to an apparent increase in cortical thickness prior to the onset of folding. Following the initiation of buckling (approximately GW 28, $t \approx 0.35$), the accumulated compressive stress is progressively relaxed through the formation of folds. At this stage, cortical thickness reaches a peak and subsequently decreases as the system transitions from a pre-buckling compressive regime to a post-buckling bending-dominated regime[8]. This evolution demonstrates that apparent thickness changes can arise purely from mechanical deformation[22], even in the absence of explicitly prescribed radial growth. During the folding process, the cortical sheet undergoes differential bending deformation, resulting in a non-uniform strain distribution across its thickness[10]. Specifically, in gyral regions, local bending and compressive stresses tend to produce apparent thickening, whereas in sulcal regions, tensile stretching and curvature-induced deformation lead to apparent thinning[23]. This spatial redistribution of thickness is therefore an intrinsic consequence of folding mechanics rather than a

direct result of regionally prescribed growth[21]. This discussion has been included in the Supplementary Materials (see section "Tangential growth-dominated folding modulates cortical thickness variations").

Finally, regarding the choice of linear tangential growth, our growth functions were identified using a symbolic regression framework with constrained operator complexity to balance accuracy and interpretability (Fig. S2). Under these constraints, the training consistently selected linear functions for tangential growth from predefined operators (exp, log, polynomial, and tanh). We acknowledge that alternative forms (e.g., exp) are possible, particularly with different operator priors (e.g., defining lower complexity for favored parameters). We chose the linear growth as our final tangential growth model format because linear growth is widely supported by prior imaging studies[1,11,24] and yields quadratic-like surface area expansion trends consistent with observations[25]. In addition, as demonstrated in Fig. 6, folding patterns are more sensitive to the magnitude of growth than to the specific functional form, which further supports the robustness of our modeling choice. We have clarified this justification in the revised manuscript.

3. While authors address several limitations in the discussion section, they should also recognize limitations inherent in the relatively coarse regions selected for this analysis. What if the true regional differences lie outside these predefined boundaries? The selected regions, if too coarse, may dilute the magnitude of spatial differences and obscure the true pattern. How well do the current patterns correspond to reported biological gradients? ROI-derived growth values also depend heavily on segmentation/labeling algorithms, which may or may not accurately capture evolving boundaries over time. These factors can drastically influence the estimated growth rates.

Response and Action: Thank you for this thoughtful comment regarding the potential limitations associated with the use of relatively coarse cortical regions and the dependence on parcellation methodology.

In this study, the cortical regionalization was not arbitrarily predefined but derived from our previously established developmental parcellation framework. Specifically, this regionalization was obtained using non-negative matrix factorization (NMF) applied to vertex-wise cortical

surface area data from nearly 1,000 MRI scans spanning prenatal and neonatal development. This approach partitions the cortical surface into distinct regions by grouping vertices that share similar developmental trajectories of surface area expansion. The optimal number of regions was determined by jointly optimizing the silhouette coefficient and reconstruction error to ensure both intra-region coherence and inter-region separability. Importantly, this method defines regions based on shared developmental dynamics rather than anatomical boundaries, which helps mitigate the concern that coarse predefined regions may obscure underlying spatial patterns. Although the final parcellation includes a limited number of regions (18 regions per hemisphere), each region exhibits internally consistent growth trajectories, providing a biologically meaningful representation of large-scale developmental organization.

We agree, however, that any region-based representation introduces a level of spatial averaging. As the reviewer noted, finer-scale or vertex-wise variations may exist within or across these regions. In this sense, the current framework may attenuate localized gradients or shift boundaries relative to the "true" underlying biological pattern. Consistent with this, prior longitudinal work by Garcia, et al. [1] reported vertex-wise growth gradients and dynamic spatial shifts over development. In comparison to their work, our results, despite distinction in resolution and methodology, show strong qualitative agreement with these reported biological patterns. Specifically, both studies identify relatively higher expansion in temporal, parietal, and occipital regions and comparatively lower expansion in medial and central areas during mid-to-late gestation. Some discrepancies are also observed, particularly in prefrontal and insular regions, where our region-averaged analysis suggests elevated growth compared to vertex-wise studies. These differences likely arise from the smoothing effect of regional averaging; as the reviewer noted, such averaging can obscure fine-scale spatial heterogeneity, which is an inherent limitation of region-based approaches.

Regarding the dependence on segmentation and labeling, we acknowledge that ROI-derived growth estimates are influenced by the underlying parcellation and surface processing pipeline. In this study, we tried to mitigate this effect by adopting a regionalization derived from a large-scale

dataset with consistent preprocessing, together with a fully data-driven NMF-based regionalization method. These choices reduce reliance on artificial constraints and minimize the introduction of artificial biases. However, we agree that evolving cortical boundaries during development and segmentation approach variability remain important considerations that influence regional growth estimates.

We have explicitly noted this limitation in the Discussion section of the revised manuscript.

Third, both the estimation of growth trajectories and subsequent analyses are performed based on parcellated regions; such regional averaging over relatively coarse parcellations may obscure fine-scale spatial heterogeneity present in real brains. In this study, the cortical regionalization map was adopted from a previously established large-scale developmental dataset rather than derived from the current cohort. This is because NMF-based regionalization requires large datasets with broad and continuous developmental coverage to reliably estimate vertex-wise developmental trajectories. Future studies with larger and more uniformly distributed datasets may enable the derivation of more robust, cohort-specific regionalization maps.

4. The trajectory comparisons in Fig. 7 are difficult to interpret. Was subcortical growth also held linear, or just the radial component of cortical growth? For the global comparisons (Fig. 7a–e), this seems crucial for understanding what drives the differences between models. (If all growth rates were assigned to any one model, there should be no difference at t=1.) In the figure caption and text, authors should explicitly describe if/how the ratio of cortical to subcortical growth rate is being altered over time and what they perceive to be the main driver of differences between models.

Response and Action: Thank you for this important comment. In original Fig. 7 (now Fig. 6), we specifically investigated how different tangential cortical growth trajectories influence folding patterns. In all cases, all the radial components of cortical growth and subcortical growth were held fixed, following the predictions shown in Fig. 2b and Fig. S4. Therefore, all variations across models arise solely from modifications to the tangential growth of the cortex.

To isolate the effect of growth trajectory/formula (rather than magnitude), we designed the models such that all growth functions share the same final cortical growth ratio. Consequently, as the reviewer noted, the models converge to similar states at the final stage ($t = 1$), and differences primarily emerge during intermediate developmental stages (Fig. 6a). The key driver of the observed differences is the time-dependent evolution of the relative growth ratio between cortex and subcortex. Since subcortical growth is identical across models, altering the cortical tangential growth trajectory changes how quickly this relative ratio increases. Models with slower increases in relative cortical expansion delay the onset of mechanical instability, leading to later fold emergence, whereas faster trajectories promote earlier folding. This explains the temporal differences observed in Fig. 6b, while the final folding patterns remain comparable due to the matched end-point growth ratios (Fig. 6c-e).

We have revised both the figure caption and the main text to explicitly state that: (i) only tangential cortical growth is varied, (ii) radial cortical growth and subcortical growth are held constant, and (iii) differences between models are primarily driven by the temporal evolution of the cortex-to-subcortex growth ratio.

Caption change:

Fig. 6 | Regional growth trajectory modulates cortical morphology. **a**: Growth models with identical final ratios but different trajectories: hyper tangent (HYT), Gompertz (GOM), symbolic regression characterization (SYR), and polynomial (POL). Growth model of region 4 is shown as an example. Trajectory variation was applied solely to the cortex tangential growth, while cortex radial growth and subcortex growth were held fixed, following the model predictions shown in Fig. 2b and Fig. S4.

Main text change:

This behavior is governed by the time-dependent evolution of the relative cortex-to-subcortex growth ratio. With identical subcortical growth, varying cortical tangential trajectories alter how rapidly this relative ratio increases, thereby shifting the onset of folding, and leading to similar final morphologies due to matched end-point growth ratios.

We specifically focused on the role of growth functions, considering two attributes: the temporal trajectories and final growth magnitudes. Given that thickness variation plays a relatively minor

role in shaping major folding patterns, all modifications in this analysis were applied exclusively to the tangential component of cortical growth, while both radial cortical growth and subcortical growth were held unchanged.

We further explored the effect of growth ratio magnitude on folding outcomes by maintaining identical linear trajectories for cortical tangential growth across all regions, while systematically varying the end-point growth ratios.

5. The current manuscript is quite long, owing in part to extensive technical analyses that may be of limited interest to the broader Nature Communications readership. For the sake of space, certain comparisons could be omitted or moved to supplement. For example, since local gyrification index, local sulcal depth, and other curvature metrics generally show the same trends, authors may choose one and optionally provide the rest in supplement. Similarly, less impactful / biologically relevant comparisons - especially of factors that have been reported in previous studies - could be shortened, omitted or moved to supplement.

Response and Action: We thank the reviewer for this helpful suggestion. Following the reviewer's recommendation, we streamlined the main text by moving less essential or previously established analyses to the Supplementary Materials.

Specifically, original Fig. 6, which examines geometric effects that have been widely reported and uses a similar presentation format, has been moved to the Supplementary Materials to reduce redundancy and improve clarity (Fig. S8). To streamline the presentation of metrics, we retained sulcal depth as the primary measure in the main text (Fig. 5), while selectively including the local gyrification index (GI) in figures where it provides complementary information on folding complexity and enhances clarity of interpretation. For Fig. 6, we moved the analysis and discussion of growth magnitude to the Supplementary Materials to keep the main text concise and focused (Fig. S9). In addition, we shortened the Methods section by transferring detailed technical descriptions to the Supplementary Materials.

These revisions improve the overall readability and focus of the manuscript while preserving all

relevant technical details for interested readers. Please refer to the revised manuscript for the updated details.

Minor Concerns / Edits

1. For easier interpretation, consider using the same plot style (bar vs radial, etc.) for the same metric across figures and/or similar metrics within a figure.

Response and Action: Thank you for this helpful suggestion. We agree that consistent visualization improves clarity and interpretability. Accordingly, we have revised the figures to ensure consistency in plot styles. In Fig. S8c, the heat map of regional mean sulcal depth now shows deviations relative to the normal thickness case, and the radar plot is retained to highlight differences in absolute sulcal depth. In Fig. 6, panels h–i have been moved to the Supplementary Materials (Fig. S9), so that each metric is now presented using a single, consistent plot type. Please refer to the updated figures in the revised manuscript.

2. In Fig. 2 and Fig. S3, caption for panel d: I think t = 1.0 should be t = 0.1.

Response and Action: Thank you for carefully checking this detail. The label t = 1.0 in Fig. 2d and Fig. S3d (now Fig. S2d) is correct. Because the tangential growth ratios are very close at the end-point time (t = 1.0), we used a magnified view to highlight their differences. In response, we have also moved the magnified view to the right side of the figure to improve clarity and avoid confusion. Please refer to the updated figures in the revised manuscript.

3. Where Fig. S3 is first referenced, it would be better to say: "Growth patterns are not entirely symmetrical between the left hemisphere (Fig. 2) and right hemisphere (Fig. S3)." This clarifies what Fig. S3 actually is.

Response and Action: Thank you for this helpful suggestion. We have revised the text to improve clarity as follows: "Growth patterns are not entirely symmetrical between the left hemisphere (Fig. 2) and right hemisphere (Fig. S2)." Please refer to the revised manuscript for the updated wording.

4. In panel e of Fig. 4, I cannot see “real brain” results for G32, 35, 38, but the legend indicates they should be there.

Response and Action: Thank you for pointing this out. We have updated the color scheme to use more distinguishable colors for improved visibility. Please refer to the revised figure for the updated results.

5. Why halve tangential growth ratio for regional pachygyria when “full pachygyria” was based on doubling cortical thickness? This seems inconsistent and needs explanation.

Response and Action: Thank you for this insightful comment. In abnormal folding simulations, we tuned the modeling parameter following guidance from the qualitative case descriptions summarized in Table S1, rather than enforcing a single uniform manipulation across conditions. For global pachygyria, the defining feature is widespread cortical thickening with reduced sulcation. Accordingly, we modeled this condition by increasing cortical thickness while keeping the growth profile unchanged. In contrast, regional pachygyria is characterized by asymmetric hemispheres with reduced white matter and locally underdeveloped gyri. To capture this localized deficiency in cortical expansion, we reduced the tangential growth ratio in the affected regions, which delays folding and produces a smoother surface. Although increasing regional cortical thickness can also produce locally smoother folding patterns, it does not reproduce the reduced local volume observed in regional pachygyria. Therefore, we chose to solely reduce the tangential growth ratio in the affected regions.

We have clarified this rationale in the revised manuscript to avoid confusion.

In one case, pachygyria localized to the right frontal and parietal lobes presents as a smooth cortical surface with reduced cortical volume (Fig. 7d). Although increasing cortical thickness in these regions, as applied in the global pachygyria case, generates smooth folding patterns, it fails to reproduce the locally reduced volume observed in this condition. Therefore, we selectively reduced the tangential growth ratios by half in the affected regions (R1: dorsal precentral & posterior cingulate, R4: lateral precentral & postcentral, and R11: caudal middle frontal), which

effectively captures the reported abnormal folding phenotype (Movie 5).

6. In Fig. S1, labels/arrows on the left schematic are confusing. It looks like proliferation only occurs in frontal cortex (etc.) which I assume is not what you mean. If these areas are linked to specific genes, it may be better to list genes explicitly on the relevant regions. Meaning of arrows is not apparent.

Response and Action: Thank you for this thoughtful comment. The purpose of Fig. S1 was to illustrate that cortical regionalization is supported by transcriptomic and cellular evidence. However, we agree that the current schematic does not clearly convey these relationships and may be misleading, particularly regarding the interpretation of region-specific processes and gene associations. Given the difficulty in accurately representing these complex mechanisms within a simplified illustration, we have decided to remove Fig. S1 from the revised manuscript. As this figure served only as supplementary visual context and is not essential to the main results or interpretations, its removal does not affect the scientific conclusions of the study.

Reviewer #2 (Remarks on code availability):

I reviewed the Github site and it appears to have the necessary files for reproduction of their results. However, I did not review the codes in detail.

Response and Action: Thank you for reviewing the GitHub repository. We have further updated the repository to ensure that all necessary files are complete and organized for reproducibility. Please feel free to refer to the updated version for the latest materials.

References

1 Garcia, K. E. *et al.* Dynamic patterns of cortical expansion during folding of the preterm human brain. *Proceedings of the National Academy of Sciences* **115**, 3156–3161 (2018).
2 Xu, G. *et al.* Axons Pull on the Brain, But Tension Does Not Drive Cortical Folding. *Journal of Biomechanical Engineering* **132** (2010).
3 Balouchzadeh, R., Kroenke, C. D., Garcia, K. E. & Bayly, P. V. Measurement of Residual Stress in the Brain. *Journal of Biomechanical Engineering* **147** (2025).
4 Karzbrun, E., Kshirsagar, A., Cohen, S. R., Hanna, J. H. & Reiner, O. Human brain organoids on a

chip reveal the physics of folding. *Nature Physics* **14**, 515–522 (2018).
5 Bayly, P. V., Okamoto, R. J., Xu, G., Shi, Y. & Taber, L. A. A cortical folding model incorporating stress-dependent growth explains gyral wavelengths and stress patterns in the developing brain. *Physical biology* **10**, 016005 (2013).
6 Tallinen, T., Chung, J. Y., Biggins, J. S. & Mahadevan, L. Gyrification from constrained cortical expansion. *Proceedings of the National Academy of Sciences* **111**, 12667–12672 (2014).
7 Andres, S., Steinmann, P. & Budday, S. The origin of compression influences geometric instabilities in bilayers. *Proceedings of the Royal Society A: Mathematical, Physical and Engineering Sciences* **474** (2018).
8 Garcia, K. E., Kroenke, C. D. & Bayly, P. V. Mechanics of cortical folding: stress, growth and stability. *Philosophical Transactions of the Royal Society B: Biological Sciences* **373** (2018).
9 Kroenke, C. D. & Bayly, P. V. How Forces Fold the Cerebral Cortex. *The Journal of Neuroscience* **38**, 767 (2018).
10 Garcia, K. E., Kroenke, C. D. & Bayly, P. V. Mechanical stress connects cortical folding to fiber organization in the developing brain. *Trends in Neurosciences* **48**, 395–402 (2025).
11 Huang, Y. *et al.* Mapping developmental regionalization and patterns of cortical surface area from 29 post-menstrual weeks to 2 years of age. *Proceedings of the National Academy of Sciences* **119**, e2121748119 (2022).
12 Sotiras, A. *et al.* Patterns of coordinated cortical remodeling during adolescence and their associations with functional specialization and evolutionary expansion. *Proceedings of the National Academy of Sciences* **114**, 3527–3532 (2017).
13 Wang, F. *et al.* Developmental topography of cortical thickness during infancy. *Proceedings of the National Academy of Sciences* **116**, 15855–15860 (2019).
14 Thompson, E. *et al.* Non-negative data-driven mapping of structural connections with application to the neonatal brain. *NeuroImage* **222**, 117273 (2020).
15 Balouchzadeh, R., Bayly, P. V. & Garcia, K. E. Effects of stress-dependent growth on evolution of sulcal direction and curvature in models of cortical folding. *Brain Multiphysics* **4**, 100065 (2023).
16 Tallinen, T. *et al.* On the growth and form of cortical convolutions. *Nature Physics* **12**, 588–593 (2016).
17 Budday, S. & Steinmann, P. On the influence of inhomogeneous stiffness and growth on mechanical instabilities in the developing brain. *International Journal of Solids and Structures* **132**, 31–41 (2018).
18 Wang, X. *et al.* The influence of biophysical parameters in a biomechanical model of cortical folding patterns. *Scientific Reports* **11**, 7686 (2021).
19 Solhtalab, A., Foroughi, A. H., Pierotich, L. & Razavi, M. J. Stress landscape of folding brain serves as a map for axonal pathfinding. *Nature Communications* **16**, 1187 (2025).
20 Garcia, K. E., Basinski, C. & Kroenke, C. D. Quantifying the Timing of Gyral and Sulcal Formation Relative to Growth in the Ferret Cerebral Cortex. *Developmental Neuroscience* **47**, 483–497 (2025).
21 Holland, M. A. *et al.* Folding drives cortical thickness variations. *The European Physical Journal Special Topics* **229**, 2757–2778 (2020).
22 Wang, S., Demirci, N. & Holland, M. A. Numerical investigation of biomechanically coupled growth in cortical folding. *Biomechanics and Modeling in Mechanobiology* **20**, 555–567 (2021).
23 Holland, M., Budday, S., Goriely, A. & Kuhl, E. Symmetry breaking in wrinkling patterns: Gyri are

universally thicker than sulci. *Physical review letters* **121**, 228002 (2018).
24 Xia, J. *et al.* Fetal cortical surface atlas parcellation based on growth patterns. *Human Brain Mapping* **40**, 3881–3899 (2019).
25 Xu, X. *et al.* Spatiotemporal Atlas of the Fetal Brain Depicts Cortical Developmental Gradient. *The Journal of Neuroscience* **42**, 9435 (2022).
