# Supplementary material for: Biophysical modeling of anatomically realistic prenatal cortical folding development
Source: Nat Commun. 2026 Jul 16;17:8711. doi: 10.1038/s41467-026-75582-9 (PMC13490475; doi:10.1038/s41467-026-75582-9)
Supplement: Supplementary file 3 [file 41467_2026_75582_MOESM1_ESM.pdf]

SUPPLEMENTARY MATERIALS

Biophysical modeling of anatomically realistic prenatal cortical folding development

Jixin Hou[1], Zhengwang Wu[2], Kun Jiang[1], Taotao Wu[3], Lu Zhang[4], Dajiang Zhu[5], Wei Gao[6], Mir Jalil Razavi[7], Tianming Liu[8], Ellen Kuhl[9], Gang Li[2], Xianqiao Wang[1]*

*Corresponding Author: xqwang@uga.edu

ORCID of Xianqiao Wang: https://orcid.org/0000-0003-2461-3015

ORCID of Jixin Hou: https://orcid.org/0000-0002-5073-0701

Containing:

Supplementary methodology:

“Symbolic regression for growth models characterization”,

“Cortical area regionalization”,

“Quantitative metrics definition”.

Supplementary discussion:

“Tangential growth-dominated folding modulates cortical thickness variations”,

“Effect of varying subcortical growth in modeling cortical folding”,

“Stress fields in simulated brain”,

“Effect of stress-dependent growth in modeling cortical folding”.

Supplementary tables and figs:

Tables. S1 to S2

Figs. S1 to S13

Movies S1 to S7 are provided separately

Symbolic regression for growth models characterization

Inspired by Darwinian principles of natural selection, symbolic regression automatedly discovers mathematical relationships directly from data without assuming a predefined functional form, thereby offering high flexibility in model discovery. Symbolic regression is implemented through genetic programming, where candidate functions are efficiently represented as binary tree structures composed of variables, mathematical operators (unary or binary), and constants (Fig. S1a). During training, the algorithm emulates evolutionary processes using tree-based operations, such as evaluation, selection, mutation, and crossover. Mutation introduces diversity by randomly modifying nodes within an expression tree, while crossover generates new candidate functions by recombining components from different parent trees. These processes are repeated iteratively until either an optimal function is identified, or the maximum number of generations is reached.

All symbolic regression analyses were performed using PYSR[1], an open-source package built on Julia. Training was restricted to a limited operator set: binary operators included addition (+), multiplication (*), and polynomial terms (t^n), while unary operators were constrained to exponential (exp), hyperbolic tangent (tanh), natural logarithmic (ln) functions (Fig. S1b). Each training run was capped at 30 minutes or 1,000 iterations, whichever occurred first, as most qualified models converged within this range during preliminary testing. The maximum expression tree depth was set to 10, and model complexity was constrained to 100. We used the default convergence criterion ("best") to guide the model selection process, which efficiently balances fitting accuracy and model simplicity. To satisfy growth ratio normalization, the predicted growth function was constrained to $g(t = 0) \equiv 1$, implemented through a penalty term in the customized objective function. For each brain region, training was repeated at least three times, and the best-performing candidate was selected as the final model. All analyses were conducted on a Legion PC equipped with a 12-core Intel Core i9-14900HX 2.2GHz CPU and 32GB of RAM.

Cortical area regionalization

Cortical regions were defined using a previously established data-driven developmental regionalization map derived from large-scale prenatal and neonatal neuroimaging datasets[2]. The map was generated using non-negative matrix factorization (NMF) applied to vertex-wise cortical surface area data from nearly 1,000 MRI scans, including 549 scans spanning 29-45 gestational weeks, from the developing Human Connectome Project (dHCP) and the Baby Connectome Project (BCP). Specifically, a non-negative data matrix $V \in \mathbb{R}^{i \times j}$ was constructed, where i represents the number of cortical vertices and j represents the number of scans. Each column of V corresponds to the vertex-wise cortical surface area of one scan. NMF decomposes this matrix into two non-negative matrices $W \in \mathbb{R}^{i \times k}$ and $H \in \mathbb{R}^{k \times j}$ such that $V \approx WH$, where k denotes the number of spatial components (regions), typically satisfying $k \ll i$ and $k \ll j$. The columns of W represent spatial components grouping vertices that exhibit similar developmental trajectories of surface area expansion, thereby defining coherent cortical subregions, while H describes the contribution of each component across scans.

The appropriate number of regions was determined using two complementary criteria: the silhouette coefficient, which evaluates intra-region similarity and inter-region separation based on vertex-wise correlations of developmental patterns, and the reconstruction error, defined as Frobenius norm $||V - WH||_F^2$. The optimal region number was selected based on solutions with higher silhouette coefficients and lower reconstruction errors. Finally, a hard parcellation map was generated by assigning each vertex to the component with the largest weight in W, yielding the final cortical regionalization.

Quantitative metrics definition

Global gyrification index: To quantify the folding complexity of the brain surface, we employed the global gyrification index (GI), a widely used whole-brain metric. The global GI is defined as the ratio between the total cortical surface area and the area of its enclosing convex hull[3]. In our implementation, the convex hull was approximated using an alpha-shape surface,

which fully encloses the cortex while minimizing the enclosing area to provide a more accurate estimate.

Local gyrification index: In addition to the global GI, we quantified regional folding complexity using the local gyrification index. The local GI complements the global measure by capturing spatial variations in cortical folding and providing a region- or vertex-wise characterization. To enhance accuracy, we applied the anisotropic kernel method[4], which uses wavefront propagation to construct spatially adaptive kernels that conform to cortical geometry, elongating along gyral crowns and sulcal fundi while remaining uniform over flatter regions. Compared with conventional kernels, this approach provides more reproducible local gyrification estimates that better reflect biologically relevant variations. Herein, the local GI values were computed at each vertex of the cortical surface.

Sulcal depth: Sulcal depth is another widely used quantitative descriptor of cortical folding. Several approaches have been proposed for computing sulcal depth, differing in their choice of reference surface and distance metric. In this study, we defined sulcal depth as the Euclidean distance between the cortical surface and its convex hull, consistent with the definition used for calculating the global GI[5]. For each vertex on the cortical mesh, the nearest projection onto the triangular facets of the convex hull was identified, and the minimal Euclidean distance was taken as the sulcal depth at that vertex. Given the convexity of the hull, the shortest distance is uniquely defined, ensuring stable vertex-wise measurements across the surface.

Curvatures: We consider the Weingarten curvature matrix to quantify curvatures in all directions. Its eigenvalues define the principal curvatures, k_1 and k_2, corresponding to the maximum and minimum normal curvatures. From these, the mean curvature is given by $k_H = (k_1 + k_2)/2$, and the Gaussian curvature by $k_G = k_1 \cdot k_2$. Among these descriptors, mean curvature has been widely used in cortical folding studies. However, its magnitude depends on surface size and geometry, making it sensitive to brain scale. To obtain a scale-independent measure, we adopted a dimensionless mean curvature[5], defined by multiplying k_H with a characteristic length l_c:

$$l_c = \sqrt{A_s/4\pi} \quad \Rightarrow \quad k_H^* = k_H \cdot l_c, \tag{1}$$

where A_s is the surface area. Dimensionless mean curvatures were computed at each vertex of the triangular mesh and, for simplicity, are referred as "mean curvature" in the main context. In addition, we defined the shape index (SI) as another scale-independent descriptor:

$$\mathrm{SI} = \frac{2}{\pi}\arctan\left(\frac{k_H}{\sqrt{k_H^2 - k_G}}\right), \tag{2}$$

with values confined to [-1, 1]. Unlike raw curvature values, this normalized metric provides a normalized characterization of local surface types. Specifically, negative values correspond to concave features such as sulci, positive values to convex features such as gyri, and values near zero to saddle-like transitional regions[6].

Sulcus number estimation: To quantify major sulci, we employed a curvature- and size-based filtering approach. Sulcal regions were identified as contiguous patches of the cortical surface with mean curvature value less than -0.05. To ensure robustness and to exclude spurious indentations, we further required a minimal surface area of 10 mm^2 and at least 60 vertices per patch. We note that this threshold-based method may underestimate the number of minor or tertiary sulci, which can be functionally relevant but are easily smoothed out by curvature filtering or mesh resolution. While curvature and size thresholds are empirically defined, this method offers a straightforward means of quantifying gross sulcus counts and supporting consistent comparison between real and simulated brains. Similar curvature-based criteria have been applied in surface-based morphometry studies of cortical folding[7].

Spherical mapping, sulcal landmarks, and similarity assessment: To facilitate comparison of cortical folding patterns, we projected the brain morphology and associated quantitative measures onto a unit sphere using FreeSurfer tools. Surface registration is an important step to ensure meaningful cross-subject comparison. In this study, correspondence between the 22-GW real brain and the undeformed simulated brain was established using multimodal surface matching (MSM), a sphere-based registration method[8, 9]. Mean curvature was used as the guiding metric, and a corresponding spherical warp was generated. Because surface connectivity is preserved throughout

the simulation, this mapping can be propagated across all computational steps, allowing registration of the simulated brain at any time point. For consistent visualization, we defined two element intersections as the north and south poles, respectively: the intersection of regions 6, 15, and 16 as the north pole, and the intersection of regions 9, 17, and 18 as the south pole (Fig. 4c).

Tangential growth-dominated folding modulates cortical thickness variations

Both OGR and TGR reproduce regional surface area trajectories well (Fig. S5a), which is expected given their identical tangential growth formulation. In contrast, cortical thickness trajectories from both models deviate from the measured data, exhibiting an inverted U-shaped pattern with elevated final thickness values across most regions (Fig. S5b). These discrepancies can be attributed to several factors. First, radial growth is substantially smaller than tangential growth (typically ~0.1 vs ~1.5), and in some regions follows an inverted-U profile with near-zero net growth. This makes folding primarily driven by tangential expansion, which in turn modulates cortical thickness patterns through geometric and mechanical coupling (e.g., bending-induced deformation and curvature-dependent strain redistribution). As a result, apparent thickness changes can arise even in the absence of explicitly prescribed radial growth, as detailed in the following mechanics discussion. This coupling provides a mechanistic explanation for why both OGR and TGR exhibit similar regional cortical thickness trajectories. Second, the absence of skull confinement in the model allows continued outward expansion of gyri, leading to increased cortical thickness relative to *in vivo* conditions. In addition, the use of a purely hyperelastic material model neglects viscoelastic relaxation, which in real brain tissue would mitigate thickness accumulation. Together, these factors explain why the simulated cortical thickness at the plateau stage is higher than that observed in real brains. Finally, another potential factor is that, in our simulations, cortical thickness and surface area expansion evolve simultaneously, whereas imaging studies suggest that cortical thickening precedes areal expansion during development[10]. This divergence in developmental progression may lead to distinct morphological outcomes. Despite these limitations, both models capture the overall inverted U-shaped thickness trend observed in real data (Fig. 2b). Notably, OGR generally shows improved, or at least comparable,

agreement with measured cortical thickness compared to TGR across most regions (e.g., regions 1, 2, 3, 12, 15, and 17). These results suggest that incorporating regionally prescribed thickness contributes to more realistic developmental trajectories.

In the simulations, early tangential expansion of the cortical layer against the relatively constrained subcortical substrate generates in-plane compressive stress[11]. Due to the near-incompressibility of brain tissue, this tangential compression is partially accommodated by out-of-plane deformation, leading to an apparent increase in cortical thickness prior to the onset of folding. Following the initiation of buckling (approximately 28 GW, $t \approx 0.35$), the accumulated compressive stress is progressively relaxed through the formation of folds. At this stage, cortical thickness reaches a peak and subsequently decreases as the system transitions from a pre-buckling compressive regime to a post-buckling bending-dominated regime[12]. This evolution demonstrates that apparent thickness changes can arise purely from mechanical deformation[13], even in the absence of explicitly prescribed radial growth. During the folding process, the cortical sheet undergoes differential bending deformation, resulting in a non-uniform strain distribution across its thickness[14]. Specifically, in gyral regions, local bending and compressive stresses tend to produce apparent thickening, whereas in sulcal regions, tensile stretching and curvature-induced deformation lead to apparent thinning[15]. This spatial redistribution of thickness is therefore an intrinsic consequence of folding mechanics rather than a direct result of regionally prescribed growth[16].

Effect of varying subcortical growth in modeling cortical folding

To examine the effect of subcortical growth magnitude on cortical folding, we performed additional simulations by modifying the subcortical growth profile while keeping the heterogeneous cortical growth model unchanged. Specifically, we considered two additional scenarios: (1) decreasing subcortical growth (Dec, $g_w(t) = 1$), in which subcortical active growth is suppressed, and (2) increasing subcortical growth by doubling the growth rate (Inc, $g_w(t) = 0.82t + 1$). In all cases, cortical growth remained identical to that used in the manuscript, while subcortical growth remained isotropic.

As shown in Fig. S11a, increasing subcortical growth leads to a noticeable enlargement of the overall brain volume. However, the simulated primary folding patterns remains largely preserved, particularly when comparing the Dec and normal growth (Nor) cases. Although some local variations are observed, for example in Region 11 (caudal middle frontal) of the left hemisphere, the Dec case exhibits fewer sulci compared with the Nor case; however, the majority of the primary sulci remain preserved. In the Inc case, the primary gyri become thicker while the sulci become relatively shallow, often accompanied by small superficial folds. Similar shallowing trends can also be observed when comparing the Nor case with the Dec case. These observations are confirmed by quantitative analysis of regional sulcal depth, defined as $\Delta\mathrm{sulc} = (\mathrm{sulc}_{\mathrm{Dec/Inc}} - \mathrm{sulc}_{\mathrm{Nor}})/\mathrm{sulc}_{\mathrm{Nor}}$. As seen in Fig. S11b, the regional mean sulcal depth increases in all regions in the Dec case, whereas it decreases in the Inc case, consistent with the morphological patterns observed in Fig. S11a. Despite these differences in sulcal depth, the number of primary folds remains nearly identical across the three cases. As seen in Fig. S11c, both global and regional sulcal counts are comparable, with the Dec case showing slightly fewer folds due to a longer folding maturation range (Fig. S11d). Furthermore, analysis of the folding development history shows that noticeable folds emerge relatively later when subcortical growth is increased, resulting in a shorter maturation window for sulcal deepening and merging.

In addition to isotropic subcortical growth, we further investigated the effects of regional heterogeneity in subcortical growth. As shown in Fig. S12a, we partitioned the subcortex into 12 regions (six per hemisphere) by roughly combining the 18 cortical regions used in our study into larger regional patches. The resulting regions approximately correspond to the temporal lobe, occipital lobe, parietal lobe, central frontal lobe, frontal lobe, and insula (Regions 1-6). We note that this regionalization was introduced solely to explore potential effects of regional subcortical growth and does not represent a precise anatomical parcellation. Using this regionalization, we implemented three heterogeneous subcortical growth scenarios by modifying regional growth ratios relative to the baseline subcortical growth ($g = g_w$), as illustrated in Fig. S12b. To further explore possible effects of regional subcortical variation, we also included regional shrinkage by

assigning negative growth ratios. Although such shrinkage does not represent typical developmental processes, it may serve as a simplified proxy for abnormal growth conditions associated with brain malformations. In all cases, the cortical growth profile remained identical to that used in the manuscript.

In heterogeneous case 1, degrowth was applied to the prefrontal regions (R4 and R5) in both hemispheres. Compared with the normal brain case (Fig. S12c), this regional degrowth produces noticeable local volume reduction, leading to wider and deeper sulci and thinner gyri in the affected regions, while folding patterns in the unaffected regions remain largely unchanged (Fig. S12d). In heterogeneous case 2, asymmetric regional growth patterns were assigned between hemispheres. Specifically, we amplified the growth in parietal areas (R3) of left brains by doubling the growth ratio, while applying degrowth to corresponding region in the right hemisphere. Similar asymmetric assignments were applied to the temporal (R1) and frontal regions (R4 and R5). As seen in Fig. S12e, these heterogeneous growth patterns produce highly non-uniform cortical folding, with degrowing regions generating deeper and wider sulci and regions with enhanced growth producing fatty gyri and shallow sulci. In heterogenous case 3, we applied degrowth to the entire left hemisphere while the right hemisphere maintained the baseline growth profile. As shown in Fig. S12f, this produces a noticeable volume reduction and folding patterns similar to those observed in other degrowth scenarios within the left hemisphere.

Stress fields in simulated brain

We analyzed the stress fields in our simulations and used the maximum (absolute) principal stress as a representative metric for both qualitative and quantitative evaluation. As shown in Fig. S7a, the cortical layer consistently experiences compressive stress, while the underlying subcortex, particularly near the gyri and sulcal fundi, exhibits localized tensile stresses. In our simulations, compressive stresses primarily develop in the cortex as a result of deformation mismatch between the rapidly growing cortex and the more slowly growing subcortex. Once these compressive stresses exceed a critical threshold, they are relieved through mechanical buckling, leading to the emergence of gyri and sulci[12, 17]. Analogous to classical bending mechanics, the subcortex beneath

gyri experiences radial tension, whereas regions beneath sulci experience tangential tension[14]. Additionally, consistent with previous biomechanical models of cortical folding, stress magnitudes in our simulation decreased with depth from the cortex-subcortex interface. The deeper subcortical regions experience near-neutral or weak compressive stresses, indicating that these tissues primarily serve as a mechanical substrate supporting cortical folding. Such stress gradients help protect deep subcortical nuclei from large deformations during cortical expansion, while allowing folding to remain localized near the cortical surface[12].

In addition to the qualitative agreement in stress distributions, the magnitudes of stresses predicted by our simulations are also consistent with values reported in the literature. To quantify this, we computed the maximum (absolute) principal stress across the ten subjects analyzed in this study and calculated the mean values for both hemispheres. As seen in Fig. S7b, the cortex exhibits an average maximum stress of approximately 0.7 kPa, while the subcortex shows an average stress of approximately 1.5 kPa. The compressive stress values fall within the range reported for the minipig brain (a gyrencephalic large mammal) in Balouchzadeh, Kroenke [18], while the tensile stress is of the same order of magnitude but slightly lower than the reported values (~3 kPa). The relatively lower tensile stress observed in our simulations may partly result from the averaging procedure used in our analysis. Specifically, stresses were averaged across the entire subcortical region and across all elements, including deeper regions that experience relatively weak compressive stresses, which reduces the overall mean value. Additional factors, such as species differences and modeling simplifications, also contribute to these quantitative differences.

Effect of stress-dependent growth in modeling cortical folding

To assess the potential impact of the viscoelasticity on folding predictions, we performed an additional set of simulations following the stress-dependent growth framework introduced by Balouchzadeh, Bayly [5]. In these simulations, the subcortex was modeled as a viscoelastic material using a Prony-series formulation, whereas the cortex remained the hyperelastic and followed the heterogeneous growth model used in the primary simulations. In this setting, subcortex continued to undergo isotropic growth, and the shear modulus relaxation was defined as:

$$\mu(t) = \mu_0 \left[(1 - g_1) + g_1 e^{-\frac{t}{\tau_1}} \right] \tag{3}$$

Here, μ_0 is the instantaneous shear modulus, g_1 represents the fraction of stress that relaxes, and the term $\mu_0(1 - g_1)$ corresponds to the long-term relaxation modulus, τ_1 defines the relaxation timescale and is expressed as a fraction of the step time T, such that $\tau_1 = \alpha_1 T$.

We specially examined the influence of the two key parameters: g_1 and α_1. Specifically, we tested three representative values for each parameter ($g_1 = 0.1,0.5,0.9$; $\alpha_1 = 0.2,0.5,0.8$), where larger g_1 corresponds to a greater fraction of stress relaxation and larger α_1 leads to slower relaxation. As shown in Fig. S13a, the primary folding patterns remain largely consistent with those obtained using the baseline hyperelastic model. However, incorporating stress-dependent effects produces slightly smoother cortical patterns compared with the baseline case. For small parameter values (e.g., $g_1 = 0.1$, $\alpha_1 = 0.2$), the simulated folding patterns are nearly identical to the baseline model. This is expected because only a small fraction of stress relaxes and relaxation occurs rapidly, resulting in minimal influence on the overall growth-driven instability. In contrast, larger relaxation parameters lead to noticeable changes in folding morphology. For example, when the relaxation fraction and timescale are increased, folds become denser with shallower sulci and smaller gyri. Quantitative analysis further supports this observation. Using the baseline model as reference, we computed the percentage deviation in sulcal depth across cortical regions (Fig. S13b). Most regions exhibit reduced sulcal depth relative to the baseline model, with only minor positive deviations observed in a few regions (e.g., R5). These results are consistent with the findings reported by Balouchzadeh, Bayly [5], who also observed reduced sulcal depth when stress-dependent growth was incorporated.

Tables and Figures

Table S1: Malformation case descriptions and corresponding modeling treatments. Case descriptions are provided in Radiopaedia.org. R1: dorsal precentral & posterior cingulate, R4: lateral precentral & postcentral, R6: inferior frontal, R11: caudal middle frontal , R16: dorsal prefrontal.

Malformation	MRI	Age	Gender	Case description	Modeling treatments
Lissencephaly	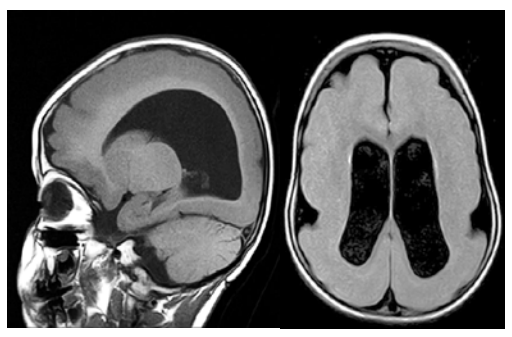	3 yr	F	Diffuse thickening of the cerebral cortex bilaterally (mean thickness = 18 mm) with few poorly formed gyri; smooth outer surface of both cerebral hemispheres with shallow Sylvian fissures.	Double cortical thickness and reduce regional growth ratios to 30%.
Pachygyria	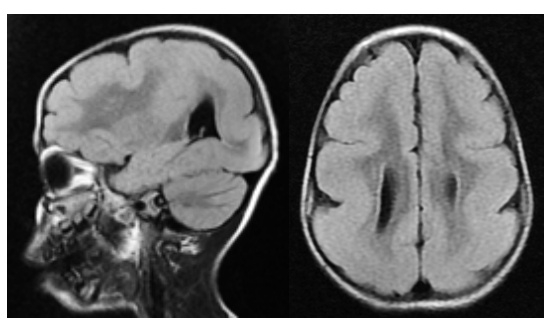	5 yr	F	Cortical thickening of the bilateral cerebral hemisphere with few sulci; grey matter heterotopia is in keeping with lissencephaly type I.	Double cortical thickness and maintain normal regional growth models.
Regional pachygyria	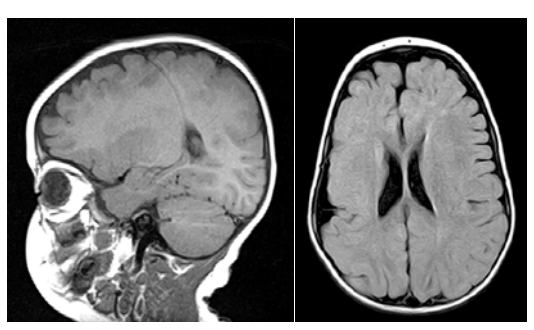	1 yr	F	Asymmetric cerebral hemispheres, the right is smaller than the left with reduced white matter and enlarged CSF spaces; thickening of the right hemispheric cortex with poorly formed gyri and a smooth outer surface.	Half tangential growth ratios in the affected regions (R1, R4, and R11).
Regional polymicrogyria	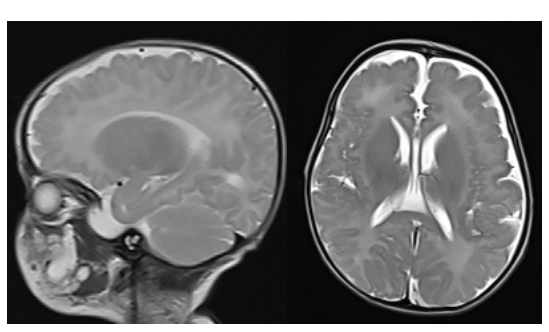	1 mo	M	Abnormal cortex with multiple small gyri, bumpy, disorganized, at superolateral frontal lobes, slightly parietal lobes, and perisylvian; more prominent on right side, with relatively abnormal sulcation.	Reduce cortical thickness by 40% in selected frontal regions (R1, R4, R6, R11, R16).
Regional polymicrogyria and hemiatrophy	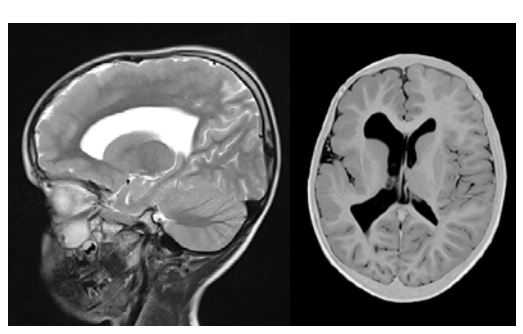	6 yr	M	The right cerebral hemisphere is small compared to the left side, showing abnormal sulcation and dysmorphic cortex; there is relative smaller size of the right caudate nucleus with ex-vacuo dilatation of frontal and occipital horns of the right lateral ventricle.	Reduce cortical thickness by 40% in regions R6 and R16, and half the growth ratios and the white matter subregions beneath.

Table S2: Model parameters used in the cortical folding simulations.

Parameter	Cortex				Subcortex			
	Density	Shear modulus	Bulk modulus	Poisson's ratio	Density	Shear modulus	Bulk modulus	Poisson's ratio
	g/cm^3	kPa	kPa	--	g/cm^3	kPa	kPa	--
Values	1.05	0.65	5.17	0.49	1.02	0.68	7.50	0.49

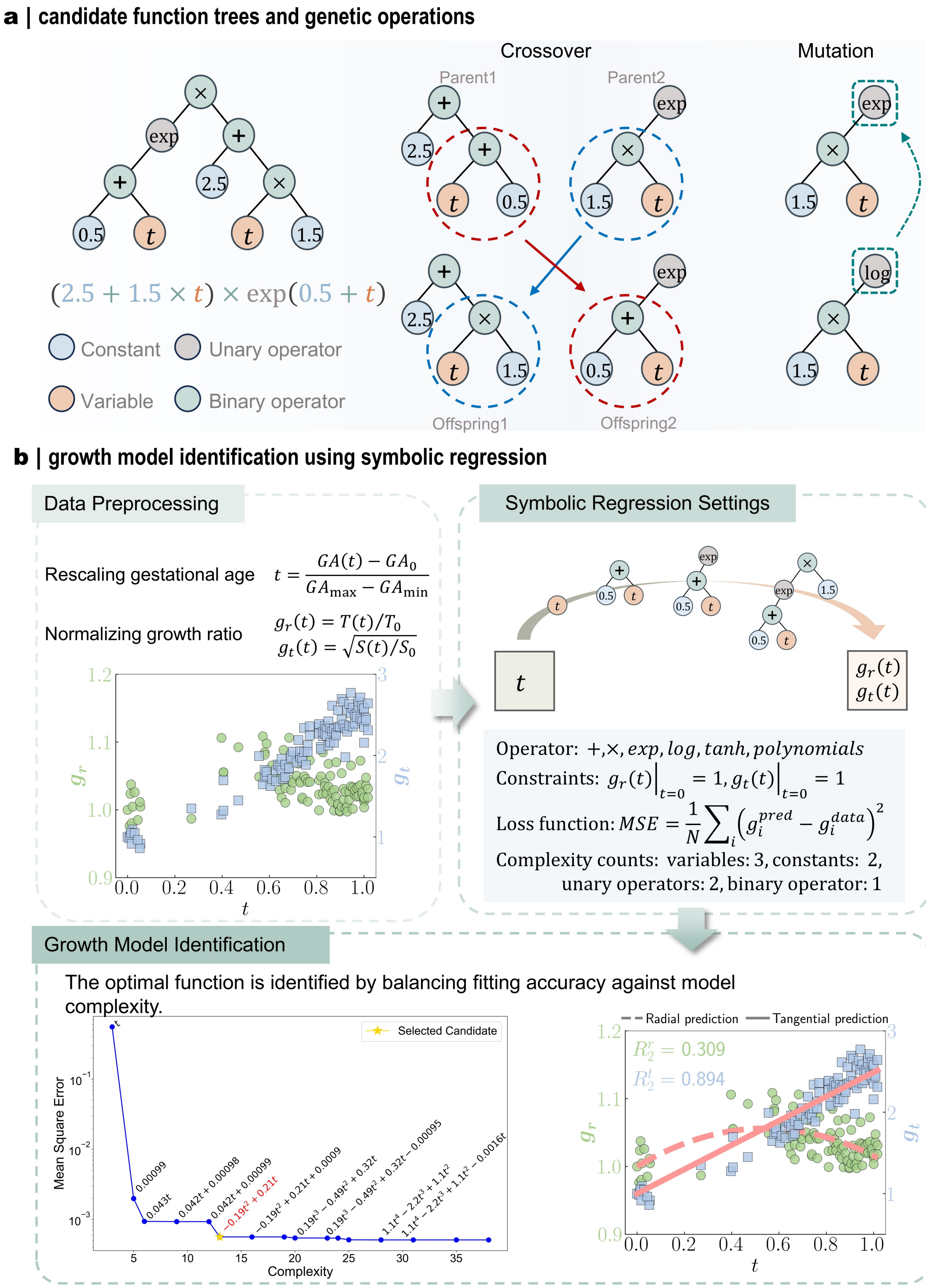

Fig. S1 | Symbolic regression framework for regional growth model characterization. **a**: Tree-structured expression of a candidate function (left) and example of crossover and mutation operations (right). **b**: Data preprocessing for regional surface area and cortical thickness (top left), symbolic regression algorithmic settings (top right), and example of determining an optimized function candidate (bottom).

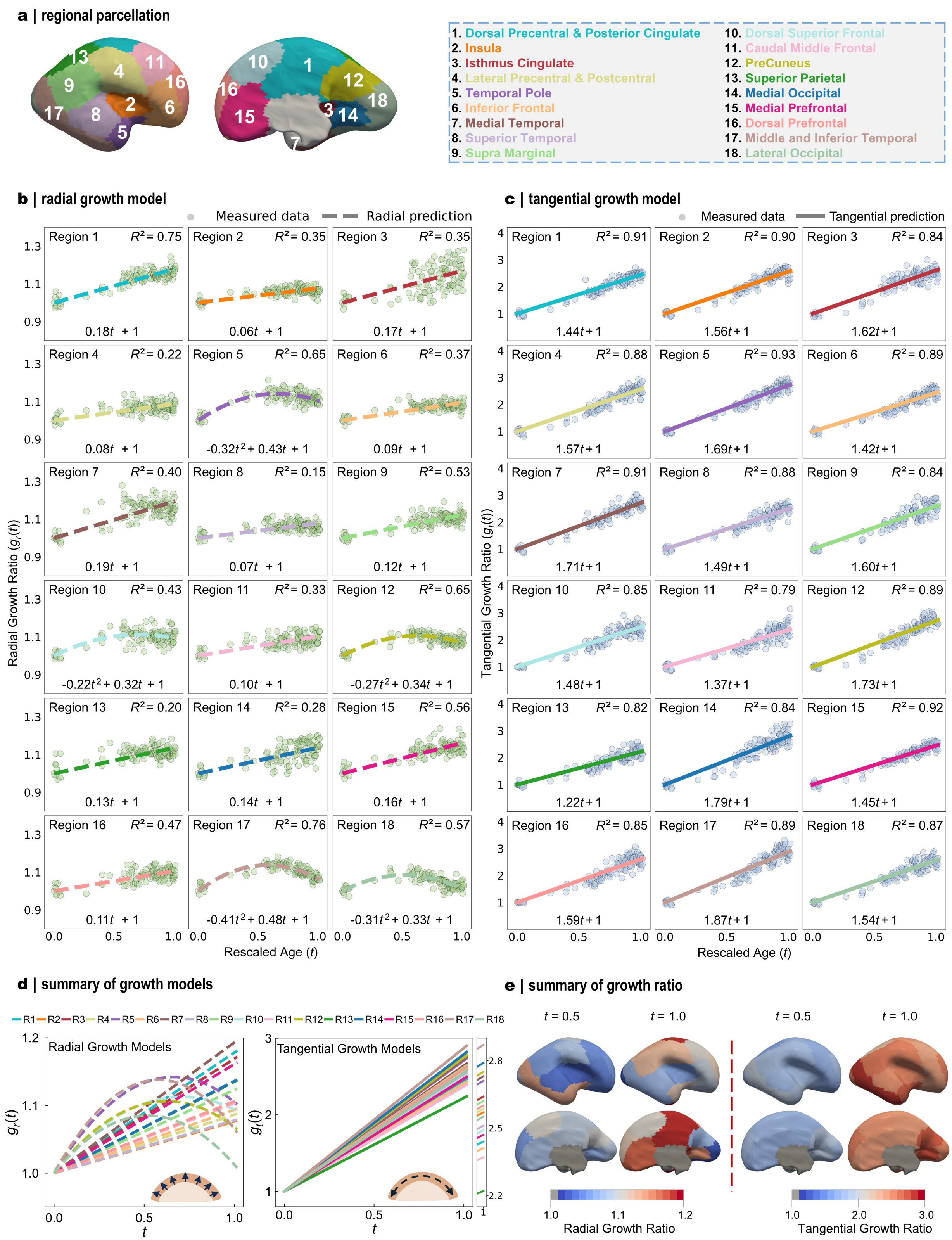

Fig. S2 | Characterization of region-specific growth models in the right hemisphere using symbolic regression. **a**: Developmental parcellation maps with corresponding anatomical regions. Distinct colors indicate

different regions, with corresponding label numbers provided; the associated anatomical regions are listed in the right panel. **b-c**: Growth models identified in radial and tangential directions. Dots denote MRI data from 22 to 40 GWs; lines indicate fitted growth models, with mathematic formulas shown at the bottom of each subfigure. R^2 indicates fitting goodness. **d**: Summary of identified regional growth model in both directions. Insets illustrate schematic representations of radial (left) and tangential (middle) growth; the right inset provides a magnified view of the tangential growth ratio near t = 1. **e**: Growth ratio values visualized on the brain surface at 22 GWs for two developmental stages. Distinct regional growth trajectories were captured in both radial and tangential directions. Source data are provided as a Source Data file.

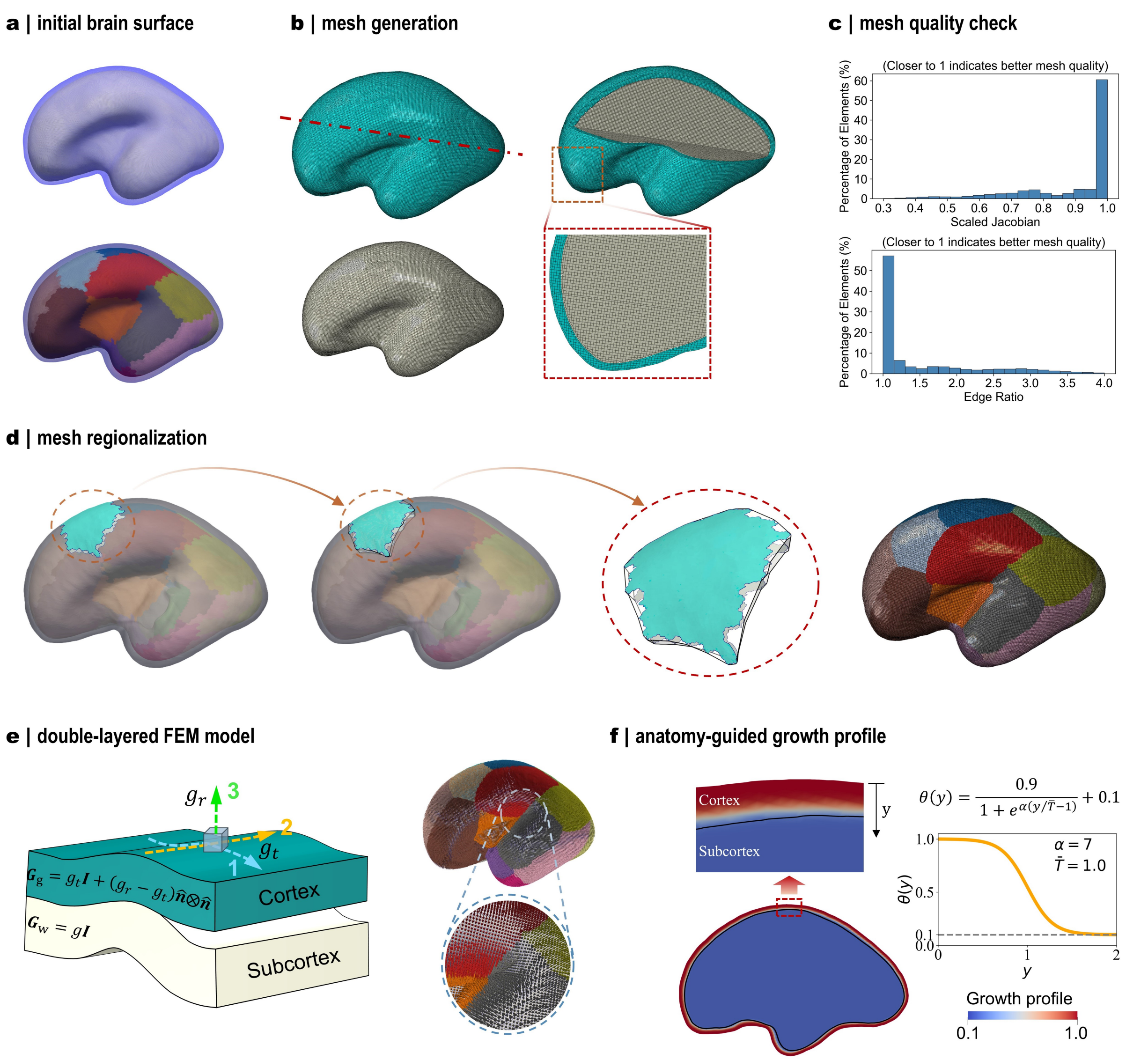

Fig. S3 | Whole-brain modeling framework. **a**: Initial gray and white matter surfaces with regional parcellation. **b**: Hexahedral mesh construction, with magnified view showing smoothed mesh near surfaces. **c**: Mesh quality check confirming good mesh quality. **d**: Mesh regionalization based on parcellation from panel a for regional material property assignment. **e**: Double-layer finite element model with distinct growth treatment for cortex and

subcortex (left), and local material orientation extraction for orthotropic growth assignment in cortex. **f**: Anatomy-guided growth profile.

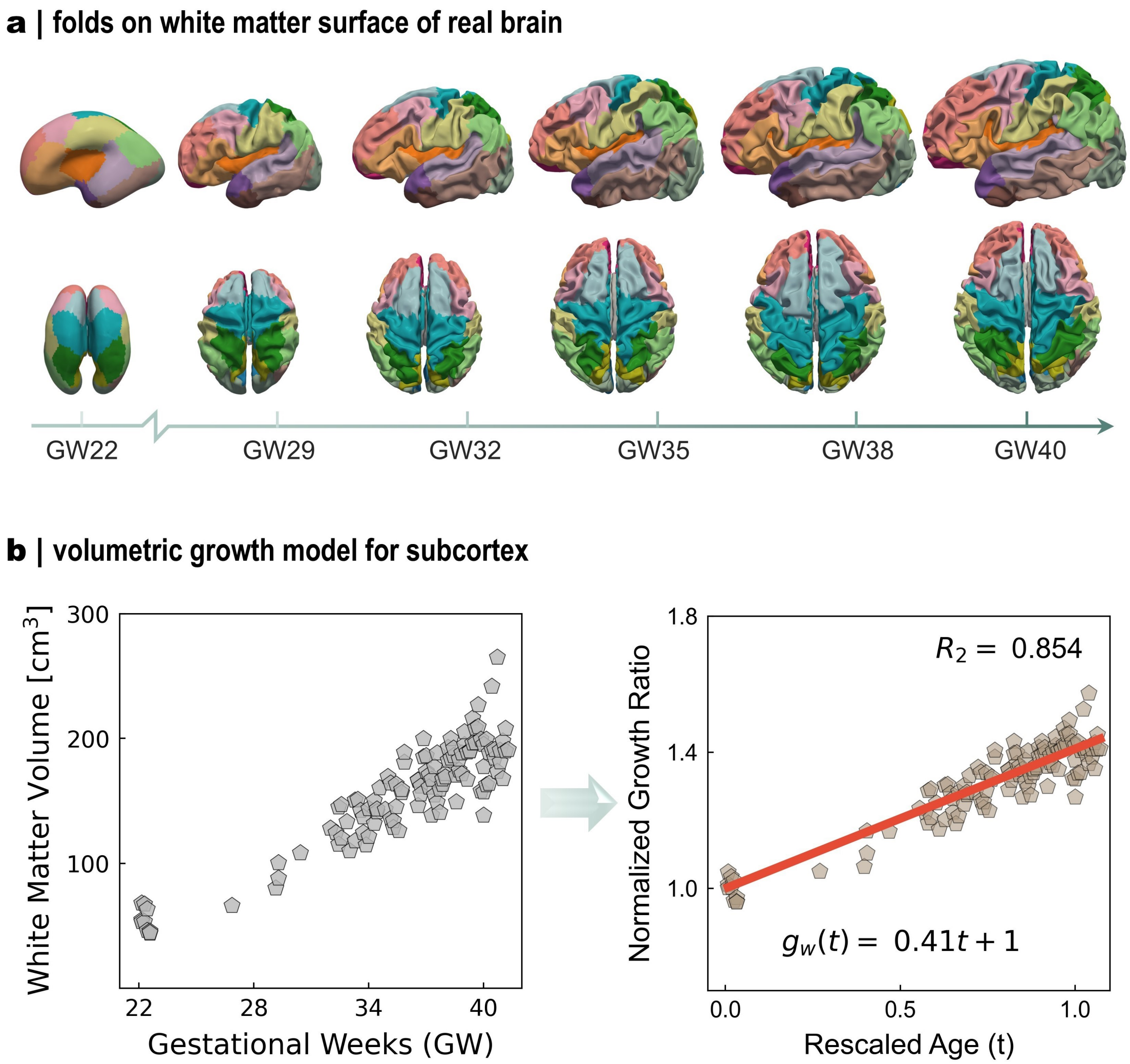

Fig. S4 | Growth model of brain subcortex. **a**: Folding pattern on white matter surface, shown from top to bottom views for the left hemisphere and the whole brain. **b**: Growth model derived for subcortex, with plots showing (from left to right) the raw volume data and the normalized growth ratios. Dots denote MRI data from 22 to 40 GWs; lines indicate fitted growth model, with mathematic formula shown below. R^2 indicates fitting goodness. Source data are provided as a Source Data file.

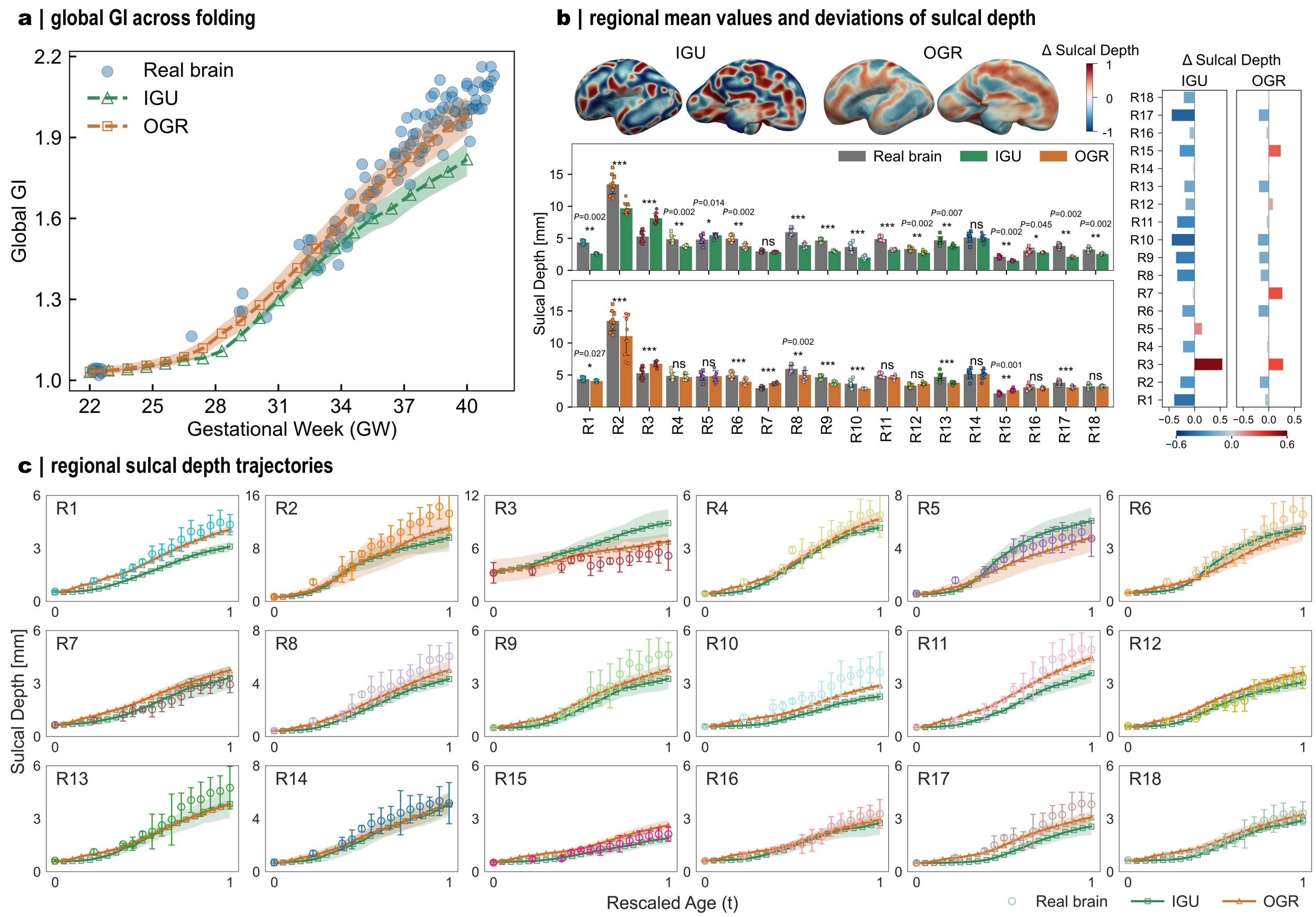

Fig. S5 | Comparison of folding metrics between uniform (IGU) and heterogeneous (OGR) growth models. **a**: Developmental trajectory of global gyrification index (GI) in real and simulated brains. Insets show real brain folding patterns for typical data points. Simulation data are shown as mean ± SD (n=10). **b**: Comparison of regional mean sulcal depth. Distribution of sulcal depth deviation (top left). Deviation is computed using the mean of ten simulated brains and the mean of real brains near 40 GWs, and is visualized on the 22 GW real brain surface. Regional sulcal depth across 18 cortical regions for simulated and real brains are summarized in the bottom left panel. Data are presented as mean values ± SD (n=10). Statistical significance was evaluated using a two-sided Wilcoxon signed-rank test. ****P* < 0.001; ***P* < 0.01; *P* < 0.05; ns, not significant, exact *P* values are provided where applicable. Right panels show regional deviations in sulcal depth relative to the real brains. **c**: Trajectories of regional mean sulcal depth across 18 regions for case of IGU and OGR. Simulation data are shown as mean ± SD (n=10). Dots and error bars represent the mean ± SD of measurements from real brains at each gestational week (n=120). Source data are provided as a Source Data file.

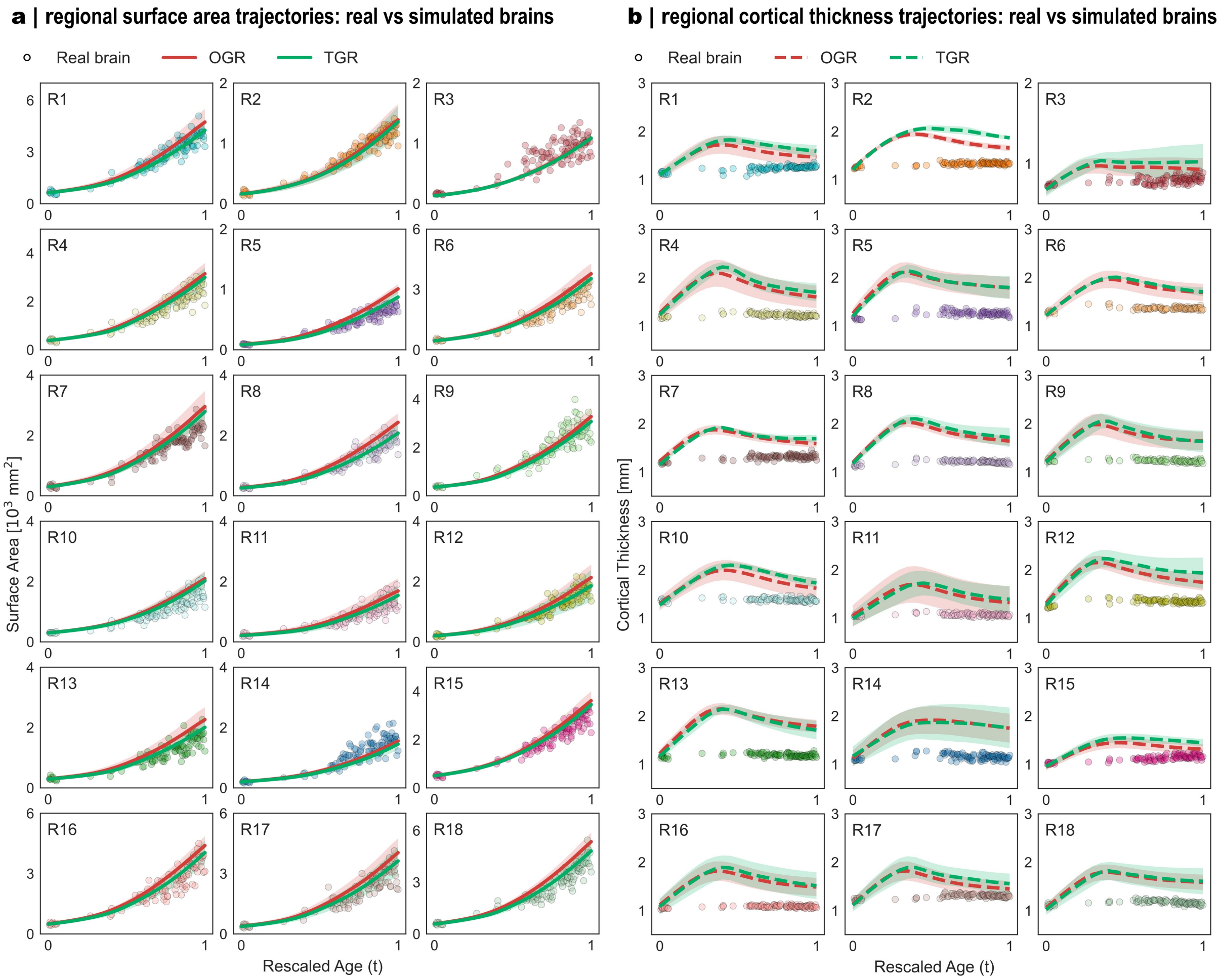

Fig. S6 | Regional trajectories of surface area and cortical thickness in real and simulated brains. **a-b**: Developmental trajectories of surface area (a) and cortical thickness (b) across 18 cortical regions. Simulation results are shown for the orthotropic regional growth (OGR) and tangential regional growth (TGR) scenarios (see Fig. 3 in the main manuscript). Simulation curves are shown as mean ± SD (n=10). Dots denote measurement from real brains obtained from 120 subjects. Source data are provided as a Source Data file.

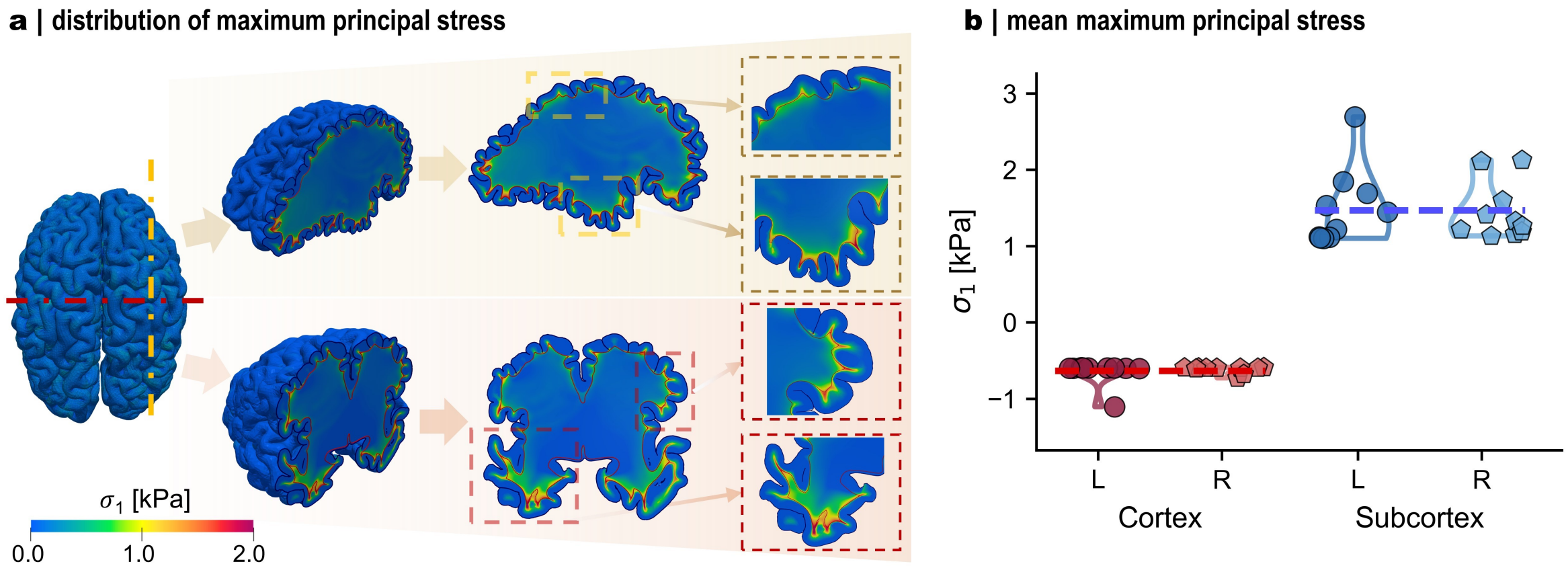

Fig. S7 | Stress distribution in simulated brains. **a**: Distribution of maximum (absolute) principal stress in the cortex and subcortex of simulated brains. Sagittal and coronal sections are shown. Insets on the right display magnified views of the local stress distribution. The cortical boundary and the cortex–subcortex interface are highlighted by black and red lines, respectively. **b**: Averaged values of maximum (absolute) principal stress in the cortex and subcortex across the ten subjects analyzed in this study. Red and blue dash lines indicate the mean values across subjects and hemispheres. Violin plots illustrate the distribution of the measurements. Cortical layer consistently experiences compressive stresses, while the underlying subcortex, particularly close to the gyrus, exhibits localized tensile stresses. Source data are provided as a Source Data file.

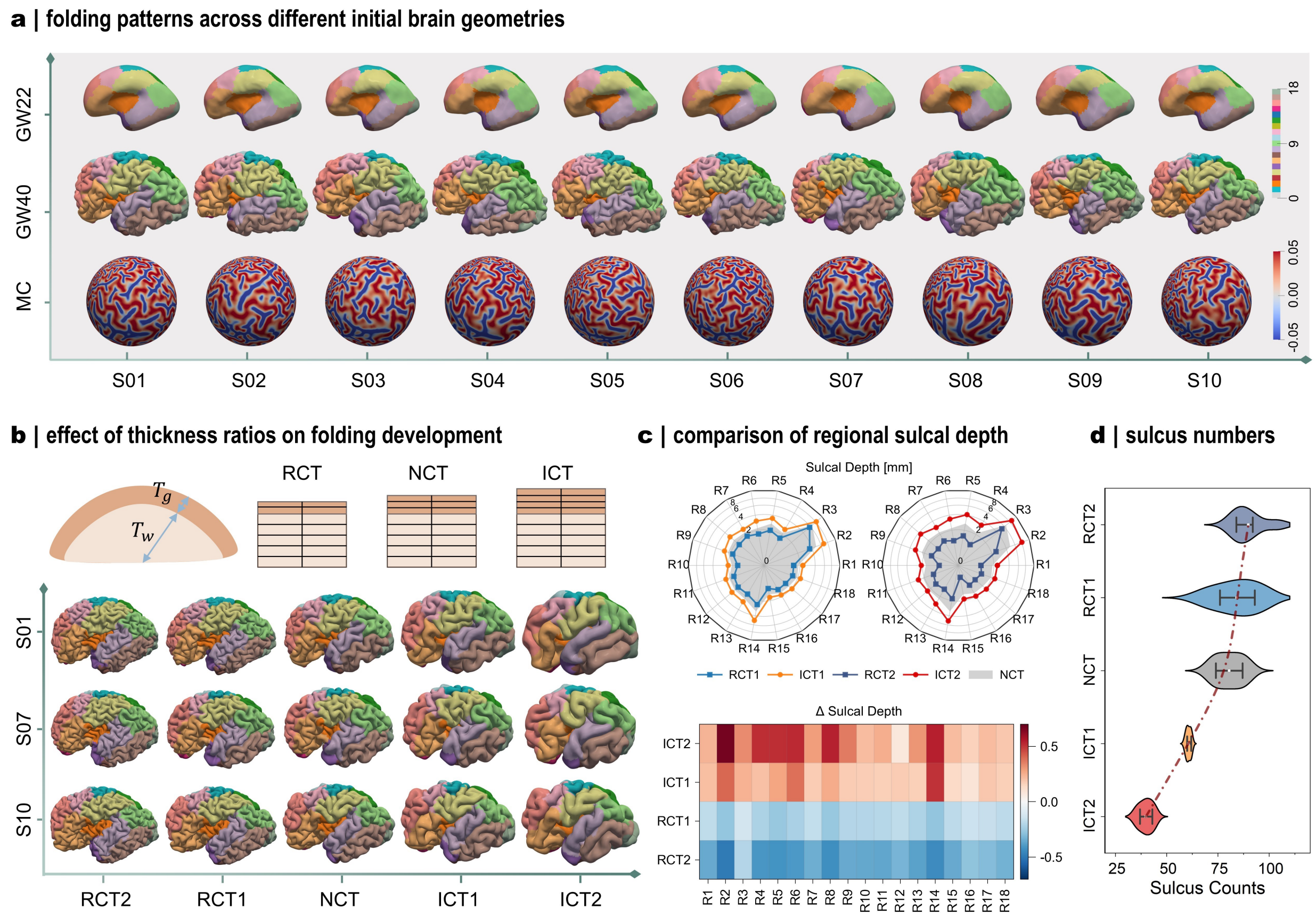

Fig. S8 | Anatomical geometry shapes cortical folding patterns. **a**: Influence of initial brain geometry on cortical folding development. Ten subjects with distinct initial geometry at 22 GWs and their corresponding folding patterns at 40 GWs are shown, with mean curvature (MC) distributions mapped onto spherical projections. Different initial geometries produced divergent folding outcomes. **b**: Influence of initial cortical thickness on folding development. Three schematic scenarios of varying cortical thickness are illustrated: reduced (RCT), normal (NCT), and increased cortical thickness (ICT). The modeled folding patterns from three representative subjects are shown below. For RCT1/ICT1 and RCT2/ICT2, the cortical surface was offset inward or outward by 3 mm and 6 mm, respectively. Altering initial cortical thickness markedly modifies folding patterns. **c**: Comparison of the regional mean sulcal depth across the five thickness cases in panel b (n = 50; 10 subjects per case). Both absolute values and deviations relative to NCT are shown. **d**: Summary of sulcus numbers across the five cases. White dot indicates medians; black box represents interquartile range (IQR), covering the 25th to 75th percentiles; whiskers, 1.5 × IQR. The dash line indicates the increasing trend of sulcus numbers with decreasing initial cortical thickness. Source data are provided as a Source Data file.

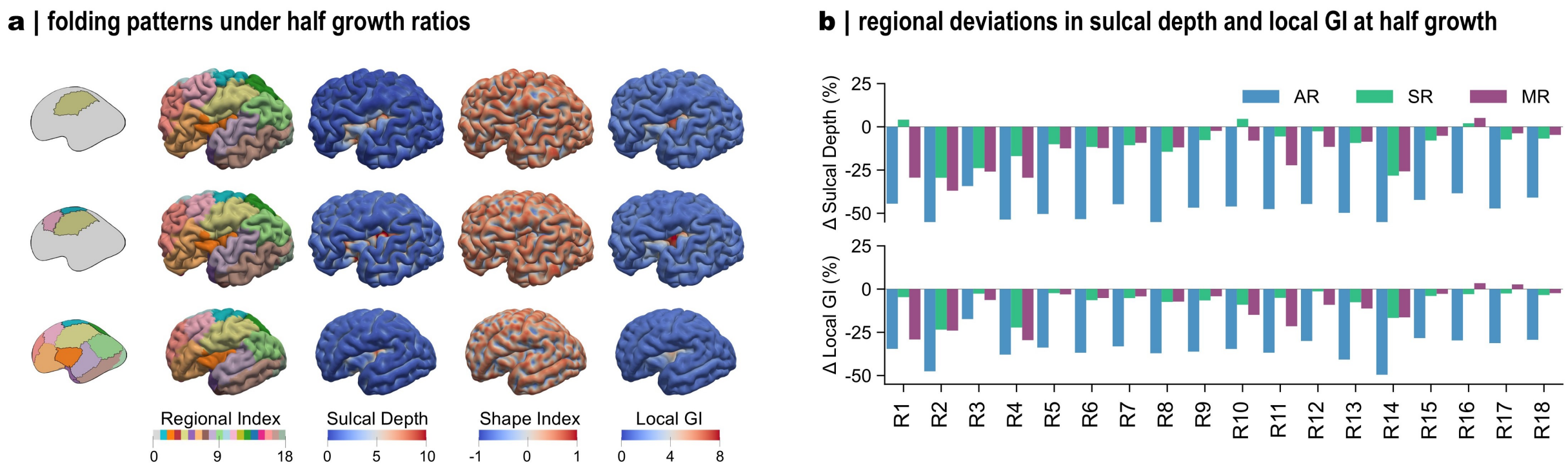

Fig. S9 | Regional growth value influences cortical morphology. **a**: Regional versus global variation of growth ratios. Folding patterns and metric distributions are shown for the same three scenarios as in Fig. 6f. Cartoon illustrates perturbed trajectories for single region (SR), multiple adjacent regions (MR), and all cortical regions (AR). **b**: Deviation of regional mean sulcal depth and local GI relative to SYR reference. Source data are provided as a Source Data file.

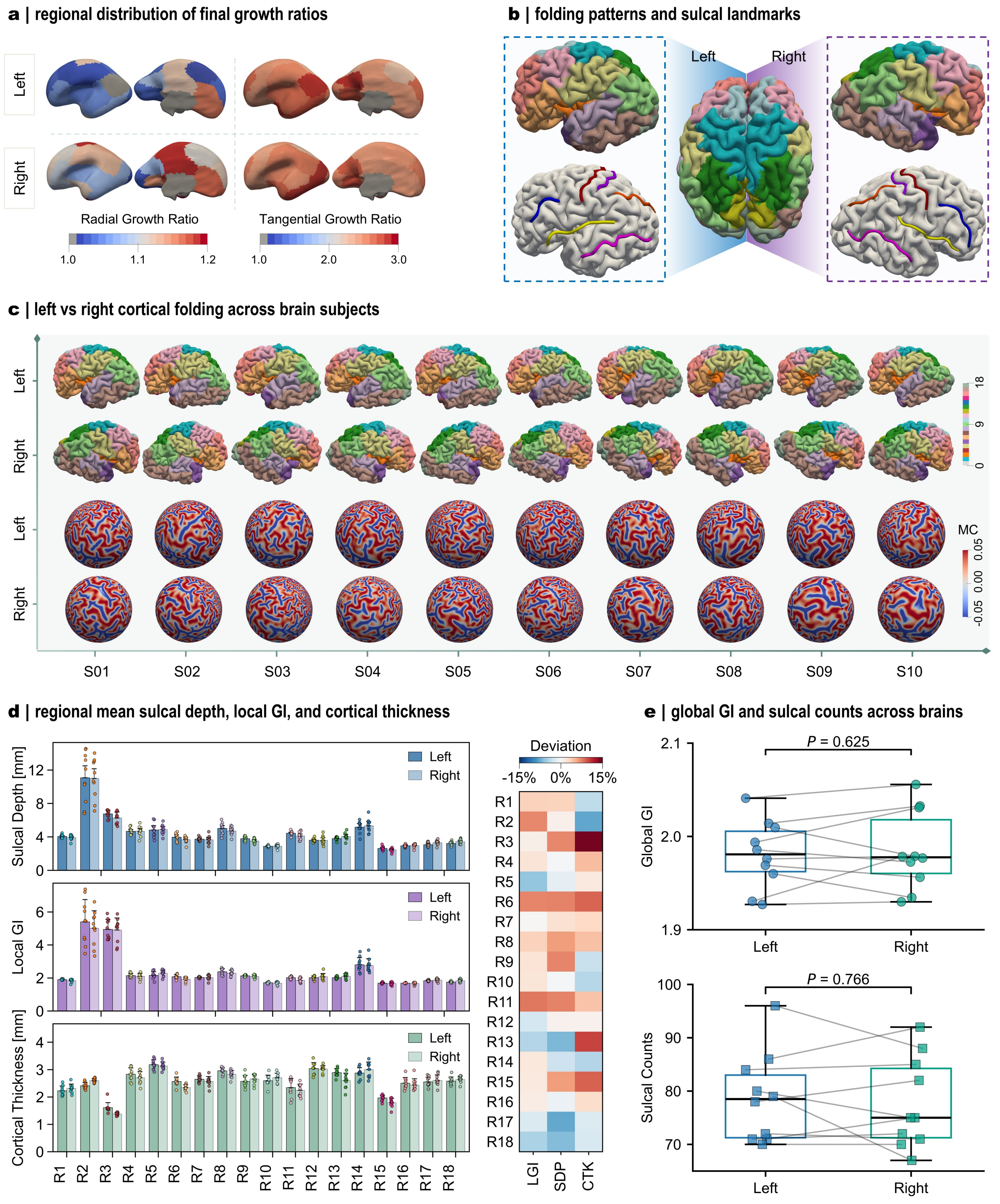

Fig. S10 | Examination of hemispheric symmetry in cortical folding patterns. **a**: Regional growth ratio at 40 GWs in left and right hemispheres (visualized on the brain surface at 22 GWs). Right hemisphere is mirrored for comparison. **b**: Folding patterns and major sulcal landmarks in both hemispheres at 40 GWs. **c**: Folding patterns of ten subjects at 40 GWs, with mean curvature (MC) distribution projected onto spheres to visualize

major sulci. **d**: Left: comparison of regional sulcal depth (SDP), local GI (LGI), and cortical thickness (CTK) between hemispheres. Right: deviations of the three metrics in the right hemisphere relative to the left. **e**: Global GI and sulcal counts in left versus right hemispheres. Boxes represent interquartile ranges (25-75%); whiskers, 1.5× IQR; individual dots represent subject values, with paired dots connected to show within-subject comparisons (n = 20; 10 subjects per case). Statistical significance was determined using the two-sided Wilcoxon signed-rank test. Source data are provided as a Source Data file.

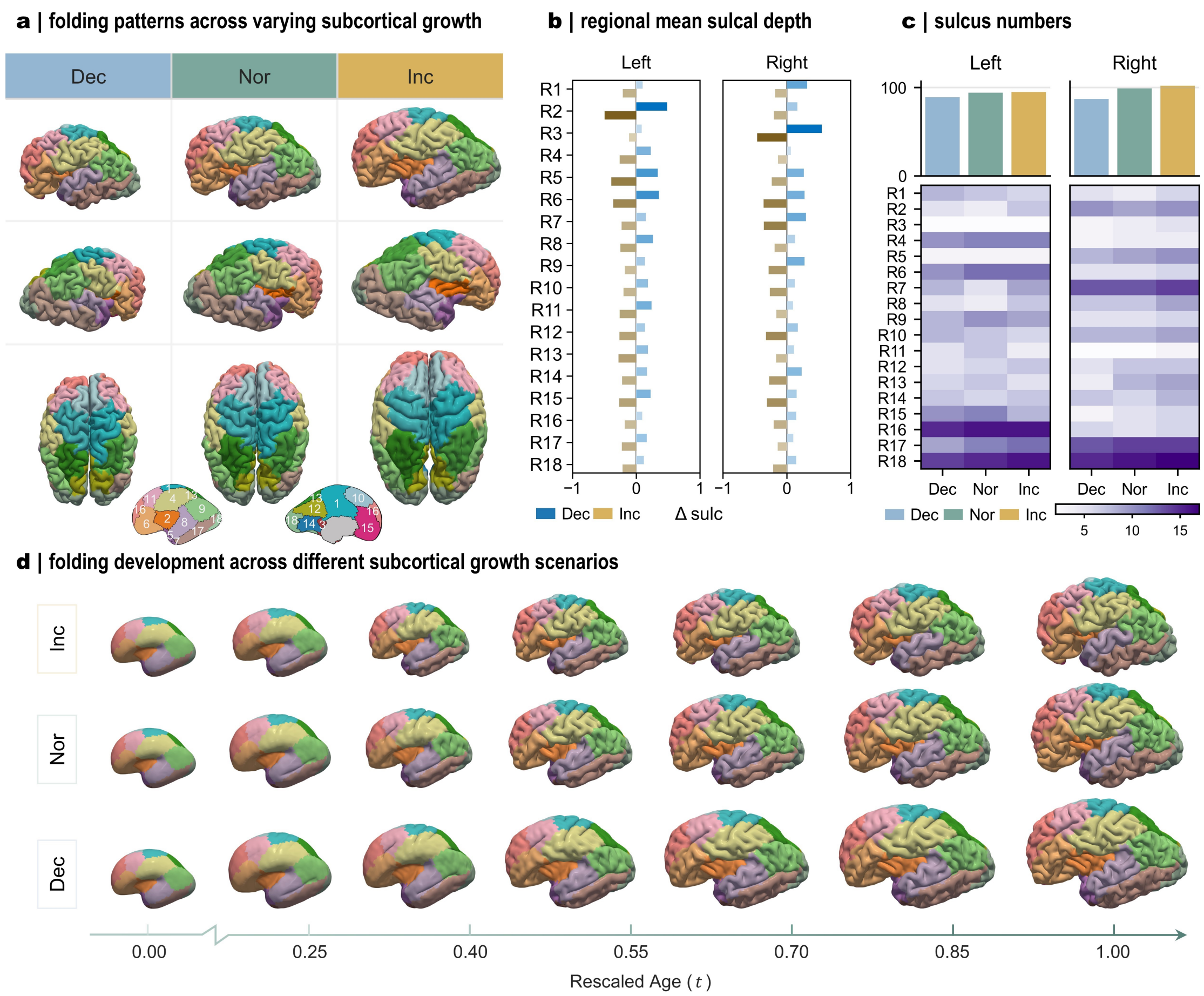

Fig. S11 | Effect of subcortical growth magnitude on cortical folding. **a**: Simulated cortical folding patterns across various subcortical growth conditions: normal growth (Nor, $g_w = 0.41t + 1$), decreased growth (Dec, $g_w = 1$), and increase growth (Inc, $g_w = 0.82t + 1$). Sagittal views of left and right hemispheres and axial views of whole brain are shown. Regional parcellation is provided at the bottom for reference. **b**: Regional difference in mean sulcal depth (sulc) for the increased and decreased subcortical growth cases relative to the normal condition ($\Delta\mathrm{sulc} = (\mathrm{sulc_{Dec/Inc}} - \mathrm{sulc_{Nor}})/\mathrm{sulc_{Nor}}$). **c**: Global (top) and regional (bottom) number of primary sulci across the three subcortical growth conditions. **d**: Representative cortical folding patterns under the three subcortical growth scenarios. In all simulations shown here, subcortical growth was treated as isotropic. Modifying subcortical growth magnitude does not substantially alter the spatial locations or wavelengths of primary folds, but it significantly affects sulcal depth and folding maturation dynamics. Source data are provided as a Source Data file.

a | subcortical parcellation

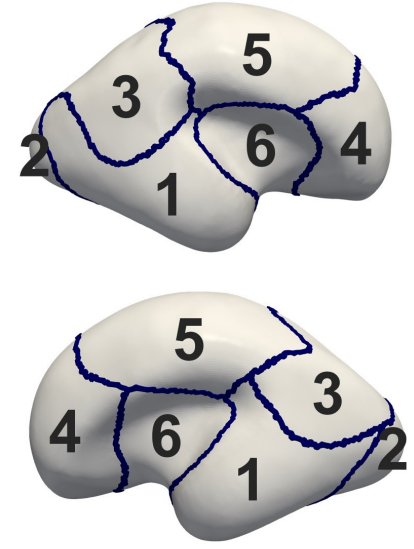

b | regional subcortical growth assignments across different cases

	Left Hemisphere						Right Hemisphere					
	R1	R2	R3	R4	R5	R6	R1	R2	R3	R4	R5	R6
Nor→	g	g	g	g	g	g	g	g	g	g	g	g
Het1→	g	g	g	$-2g$	$-g$	g	g	g	g	$-2g$	$-g$	g
Het2→	$-g$	g	$2g$	$-g$	$2g$	g	$2g$	g	$-g$	$2g$	$-g$	g
Het3→	$-g$	$-g$	$-g$	$-g$	$-g$	$-g$	g	g	g	g	g	g

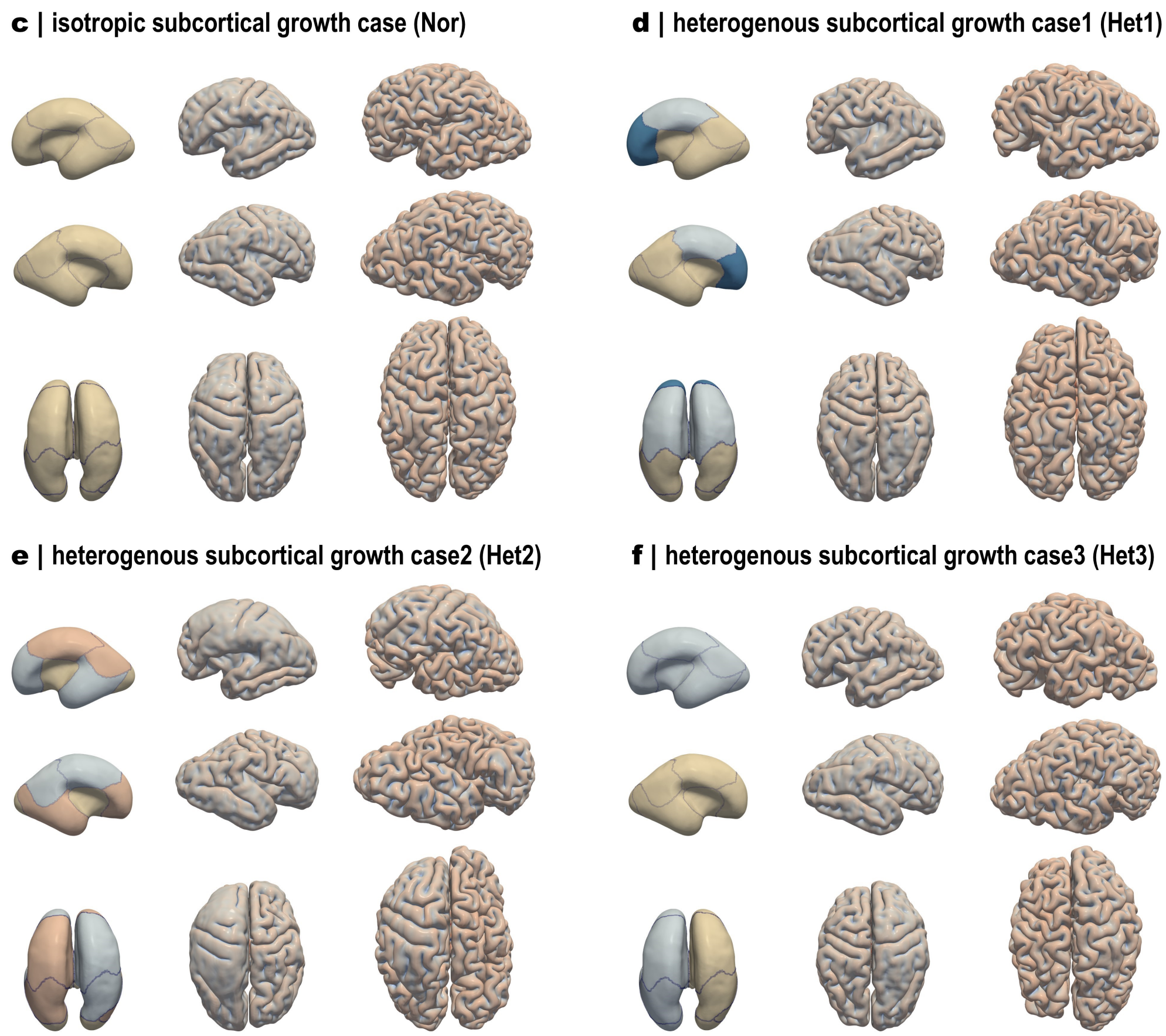

Fig. S12 | Effect of heterogeneous subcortical growth on cortical folding. **a**: Illustration of subcortical parcellation in the left and right hemispheres. Black lines denote boundaries between adjacent regions. **b**: Regional growth assignments for the isotropic growth condition (Nor) and three different heterogenous growth scenarios (Het1, Het2, and Het3). g denotes the baseline subcortical growth ($g = g_w$). **c-f**: Simulated cortical folding patterns under isotropic and heterogeneous subcortical growth scenarios. Sagittal views of left and right hemispheres and axial views of the whole brain are shown. For each case, the left panel shows the regional growth assignment (colored according to the growth ratios in panel b), the middle panel shows the folding patterns at mid-simulation ($t = 0.5$) and the right panel shows the final folding patterns ($t = 1.0$). Regional variations in subcortical growth can locally modulate cortical folding morphology and produce spatially heterogeneous folding patterns.

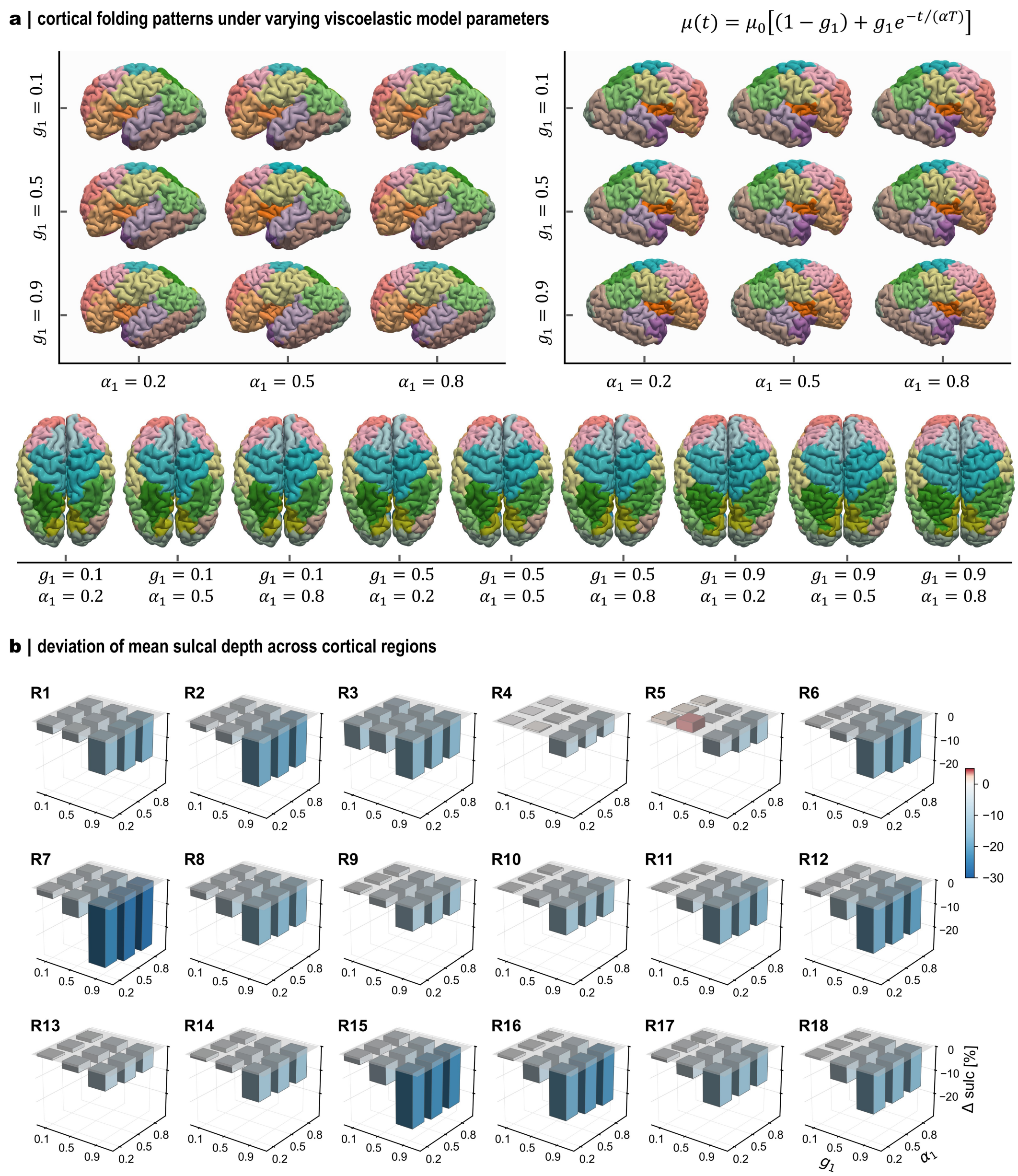

Fig. S13 | Cortical folding patterns incorporating stress-dependent growth. **a**: Cortical folding patterns under varying viscoelastic parameter settings. Sagittal views of the left and right hemispheres and axial views of the whole brain are shown. The viscoelastic model, represented by a single-term Prony series, is illustrated at the top right for reference. **b**: Percentage deviation of regional mean sulcal depth relative to the baseline model. The differential growth model without stress-dependent growth is used as the reference. The zero-difference level is indicated by a transparent gray plane. Incorporating viscoelasticity primarily smooths cortical folding patterns and slightly reduces sulcal depth, while the global organization of the primary folds remains largely unchanged. Source data are provided as a Source Data file.

References

1. Cranmer, M. Interpretable machine learning for science with PySR and SymbolicRegression. jl. *arXiv preprint arXiv:230501582*, (2023).
2. Huang, Y.*, et al.* Mapping developmental regionalization and patterns of cortical surface area from 29 post-menstrual weeks to 2 years of age. *Proceedings of the National Academy of Sciences* **119**, e2121748119 (2022).
3. Tallinen, T., Chung, J. Y., Biggins, J. S. & Mahadevan, L. Gyrification from constrained cortical expansion. *Proceedings of the National Academy of Sciences* **111**, 12667–12672 (2014).
4. Lyu, I., Kim, S. H., Girault, J. B., Gilmore, J. H. & Styner, M. A. A cortical shape-adaptive approach to local gyrification index. *Medical Image Analysis* **48**, 244–258 (2018).
5. Balouchzadeh, R., Bayly, P. V. & Garcia, K. E. Effects of stress-dependent growth on evolution of sulcal direction and curvature in models of cortical folding. *Brain Multiphysics* **4**, 100065 (2023).
6. Demirci, N. & Holland, M. A. Scaling patterns of cortical folding and thickness in early human brain development in comparison with primates. *Cerebral Cortex* **34**, bhad462 (2024).
7. Zhang, S.*, et al.* Gyral peaks: Novel gyral landmarks in developing macaque brains. *Human Brain Mapping* **43**, 4540–4555 (2022).
8. Bozek, J.*, et al.* Construction of a neonatal cortical surface atlas using Multimodal Surface Matching in the Developing Human Connectome Project. *NeuroImage* **179**, 11–29 (2018).
9. Garcia, K. E.*, et al.* Dynamic patterns of cortical expansion during folding of the preterm human brain. *Proceedings of the National Academy of Sciences* **115**, 3156–3161 (2018).
10. Garcia, K. E., Basinski, C. & Kroenke, C. D. Quantifying the Timing of Gyral and Sulcal Formation Relative to Growth in the Ferret Cerebral Cortex. *Developmental Neuroscience* **47**, 483–497 (2025).
11. Andres, S., Steinmann, P. & Budday, S. The origin of compression influences geometric instabilities in bilayers. *Proceedings of the Royal Society A: Mathematical, Physical and Engineering Sciences* **474**, (2018).
12. Garcia, K. E., Kroenke, C. D. & Bayly, P. V. Mechanics of cortical folding: stress, growth and stability. *Philosophical Transactions of the Royal Society B: Biological Sciences* **373**, (2018).
13. Wang, S., Demirci, N. & Holland, M. A. Numerical investigation of biomechanically coupled growth in cortical folding. *Biomechanics and Modeling in Mechanobiology* **20**, 555–567 (2021).
14. Garcia, K. E., Kroenke, C. D. & Bayly, P. V. Mechanical stress connects cortical folding to fiber organization in the developing brain. *Trends in Neurosciences* **48**, 395–402 (2025).
15. Holland, M., Budday, S., Goriely, A. & Kuhl, E. Symmetry breaking in wrinkling patterns: Gyri are universally thicker than sulci. *Physical review letters* **121**, 228002 (2018).
16. Holland, M. A.*, et al.* Folding drives cortical thickness variations. *The European Physical Journal Special Topics* **229**, 2757–2778 (2020).
17. Kroenke, C. D. & Bayly, P. V. How Forces Fold the Cerebral Cortex. *The Journal of Neuroscience* **38**, 767 (2018).
18. Balouchzadeh, R., Kroenke, C. D., Garcia, K. E. & Bayly, P. V. Measurement of Residual Stress in the Brain. *Journal of Biomechanical Engineering* **147**, (2025).
