# Supplementary material for: Biophysical modeling of anatomically realistic prenatal cortical folding development
Source: Nat Commun. 2026 Jul 16;17:8711. doi: 10.1038/s41467-026-75582-9 (PMC13490475; doi:10.1038/s41467-026-75582-9)
Supplement: Supplementary file 4 [file 41467_2026_75582_MOESM2_ESM.pdf]

Description of Additional Supplementary Files

Supplementary Movie 1. Cortical folding development in simulated brains (related to Fig. 3).

Supplementary Movie 2. Cortical folding development under four growth trajectories (related to Fig. 6).

Supplementary Movie 3. Cortical folding development in a simulated lissencephaly brain (related to Fig. 7b).

Supplementary Movie 4. Cortical folding development in a simulated pachygyria brain (related to Fig. 7c).

Supplementary Movie 5. Cortical folding development in a simulated brain with regional pachygyria (related to Fig. 7d).

Supplementary Movie 6. Cortical folding development in a simulated brain with regional polymicrogyria (related to Fig. 7e).

Supplementary Movie 7. Cortical folding development in a simulated brain with regional polymicrogyria and hemiatrophy (related to Fig. 7f)
